# *Toxoplasma gondii* as a Direct Cause of Reproductive Dysfunction: Dual Threats to Male and Female Fertility

**DOI:** 10.3390/vetsci13050430

**Published:** 2026-04-28

**Authors:** Muhammad Farhab, Tariq Sohail, Mohammed Al-Rasheed, Zohaib Saeed, Aftab Shaukat

**Affiliations:** 1 College of Veterinary Medicine, Yangzhou University, Yangzhou 225009, China; farhab.dvm@gmail.com; 2 Key Laboratory for Animal Genetics & Molecular Breeding of Jiangsu Province, College of Animal Science and Technology, Yangzhou University, Yangzhou 225009, China; drtariqsohail34@yahoo.com; 3 Department of Clinical Sciences, College of Veterinary Medicine, King Faisal University, Al-Ahsa 31982, Saudi Arabia; 4 Department of Pathobiology Multan, College of Veterinary Sciences, Multan University of Science and Technology, Multan 60000, Pakistan; zohaibsaeedahmad@gmail.com; 5 College of Life and Environmental Sciences, Hunan University of Arts and Science, Changde 415000, China; 6 College of Veterinary Medicine, South China Agricultural University, Guangzhou 510642, China; 7 College of Veterinary Medicine, Huazhong Agricultural University, Wuhan 430070, China

**Keywords:** congenital infection, immune dysregulation, infertility, parasite persistence, vertical transmission

## Abstract

*Toxoplasma gondii*, an obligate intracellular protozoan infecting approximately one-third of the global population, poses a significant yet underappreciated threat to reproductive health in both sexes. Although this parasite has long been linked to birth defects caused by infection during pregnancy, new research shows that it also reduces fertility in both sexes through different but related mechanisms. This review synthesizes knowledge on *T. gondii*-induced reproductive pathology across females and males, examining shared mechanistic themes while respecting tissue-specific differences, and evaluates emerging therapeutic strategies. In females, the parasite establishes persistent uterine reservoirs, triggers decidual immune dysregulation characterized by NK cell cytotoxicity, M1 macrophage polarization, Treg apoptosis, and inflammasome-mediated pyroptosis, while disrupting estrogen and progesterone signaling through both host receptor modulation and intrinsic parasite steroidogenic enzymes (*TgCYP450mt*, *TgMAPR*, *Tg-HSD*). In males, *T. gondii* breaches the blood–testis barrier, induces germ cell and Leydig cell apoptosis via ER stress and caspase pathways, impairs sperm quality parameters across acute and chronic infection, and disrupts the hypothalamic–pituitary–gonadal axis. Conserved molecular mechanisms—including *NLRP3* inflammasome activation, *PERK*/*eIF2α*/*ATF4*/*CHOP*-mediated ER stress, and oxidative stress—operate in both reproductive tissues. The parasite’s intrinsic steroidogenic capability and bidirectional hormonal manipulation represent a paradigm shift in understanding host–parasite interactions. Conventional antiparasitics face limitations due to poor reproductive sanctuary penetration. Immunomodulatory approaches targeting *Trem2*, *Tim-3*, and the *NLRP3* inflammasome show promise, along with natural products including *Inonotus obliquus* polysaccharide and ginseng polysaccharide. Nanomedicine platforms and mRNA vaccine candidates offer new directions for overcoming tissue barrier limitations. *Toxoplasma gondii* represents a fundamental threat to fertility and pregnancy outcomes rather than merely a risk for congenital infection. Integrated therapeutic strategies addressing direct parasitism, immunopathology, and endocrine disruption are needed. Longitudinal cohort studies, strain-specific mechanistic comparisons, and clinical trials of immunomodulatory adjuncts are urgently required.

## Simple Summary

*Toxoplasma gondii* can seriously affect fertility and pregnancy outcomes in both women and men. In women, the parasite can infect the uterus and placenta, disrupting the delicate immune balance needed for a healthy pregnancy. This can lead to miscarriage, stillbirth, or premature delivery—even when the baby itself is not infected. In men, the parasite invades the testes, damages sperm quality, and disrupts hormone production, potentially causing infertility. The parasite produces its own hormones and manipulates the host’s hormone systems, creating a complex interaction that affects reproduction in both sexes. Current treatments have limitations because they do not reach reproductive tissues well. However, promising new approaches using natural products, immune-modulating therapies, and nanoparticle drug delivery systems show potential. Understanding how this parasite damages reproductive health could lead to better diagnosis and treatment for couples struggling with unexplained infertility or recurrent pregnancy loss.

## 1. Introduction

*Toxoplasma gondii* infection represents a paradox in reproductive biology: an organism whose transmission depends on predation of intermediate hosts by feline definitive hosts has evolved sophisticated mechanisms to manipulate mammalian reproductive systems, potentially enhancing its spread through vertical and possibly sexual transmission routes [1,2,3]. This intracellular parasite infects at least one-third of humanity, establishing lifelong persistence in neural, muscular, and reproductive tissues through the formation of tissue cysts containing bradyzoites [3,4]. The reproductive consequences of toxoplasmosis extend far beyond the well-documented congenital toxoplasmosis syndrome. In females, acute infection during pregnancy can precipitate miscarriage, stillbirth, or severe fetal malformation [3,4], while chronic uterine persistence may compromise fertility even without active fetal infection [5,6,7]. In males, accumulating evidence from animal models and human studies indicates that *T. gondii* directly impairs spermatogenesis, reduces sperm quality, and disrupts endocrine support of reproduction [1,8,9,10]. Although the uterus and testis represent anatomically and functionally distinct reproductive organs, both constitute immunologically privileged sites characterized by tightly regulated immune environments essential for gamete development and embryonic support [11,12].

The cellular biology underlying *T. gondii*’s broad tissue tropism involves the creation of a parasitophorous vacuole (PV) that serves as a protected replicative niche. Recent work demonstrates that the host protein *MOSPD2* is recruited to the PV membrane in a strain-dependent manner, though its functional significance remains under investigation [13]. The parasite’s capacity to manipulate host cell signaling, metabolic pathways, and immune responses creates opportunities for reproductive system disruption that have only recently gained systematic attention [14,15,16,17]. *Toxoplasma gondii* transfers hormone signals through exosomes and extracellular vesicles, which deliver parasite miRNAs and proteins to host cells to modulate hormone receptor expression including *ERα* and *PRLR*. Sex-associated hormones modulate parasitic disease susceptibility and outcomes, with epidemiological and experimental evidence confirming that fluctuations in estrogen, progesterone, and testosterone directly influence both immune responses and pathogen behavior [14,15,16,17,18]. Intriguingly, *T. gondii* possesses intrinsic steroidogenic capabilities, including a mitochondrial cytochrome *P450* enzyme (*TgCYP450mt*) and membrane-associated progesterone receptor (*TgMAPR*), enabling endogenous hormone synthesis that may facilitate host manipulation [19]. Latent toxoplasmosis, previously considered harmless in immunocompetent persons, is now recognized as a potential risk factor for schizophrenia and may contribute to traffic and workplace accidents [2,20]. At least some of these effects, possibly mediated by increased dopamine and decreased tryptophan, are products of manipulation activity by *T. gondii* aiming to increase the probability of transmission from intermediate to definitive host through predation [2]. The relationship between latent toxoplasmosis and sex hormone modulation is complex and appears to differ between humans and animal models, as well as between sexes [1,21,22,23].

Our work seeks to answer three basic questions: (1) How does the parasite infiltrate reproductive tissues and remain there despite immune surveillance? (2) How can reproductive harm occur at the molecular and cellular levels? (3) Which treatment approaches aim to address these harmful processes? Toxoplasmosis is a direct cause of infertility and bad pregnancy outcomes. We want to build a coherent conceptual framework by combining findings from human studies and animal models. This literature review was conducted using PubMed as the primary database. Search terms included combinations of: “*Toxoplasma gondii*”, “reproductive dysfunction”,“fertility”, “infertility”, “pregnancy”, “testis”, “uterus”, “placenta”, “hormonal disruption”,“estrogen”, “progesterone”, “testosterone”,“immune dysregulation”, “decidual NK cells”, “spermatogenesis”, “blood–testis barrier” and “vertical transmission.” The analysis was limited to articles published in English. Additional relevant references were identified by manually screening the reference lists of retrieved articles. This review takes a narrative approach, synthesizing findings from original research, animal models, human studies, and prior reviews.

## 2. Establishing Infection in Female Reproductive Tissues

### 2.1. Female Reproductive Tract: Uterine and Placental Persistence

*Toxoplasma gondii* demonstrates a remarkable capacity to infect and persist within the female reproductive tract, establishing a local reservoir for vertical transmission and chronic inflammatory pathology. Early studies isolated the parasite from human abortus material, placenta, and endometrial biopsies, with recovery in 10% of specimens examined [24,25]. The presence of tachyzoites in cervical smears and menstrual blood confirms active replication within the reproductive tract [5,26]. Critical evidence demonstrated *T. gondii* tachyzoites in endometrial biopsies from women with habitual abortion, with five of six positive cases being serologically negative—highlighting the limitation of serological diagnosis alone. Importantly, anti-*T. gondii* treatment eliminated parasites from the endometrium, establishing a causal relationship between local infection and reproductive pathology [5].

Molecular studies in livestock demonstrate high prevalence of *T. gondii* DNA in uterine tissues of chronically infected cows and ewes, with distribution throughout the genital tract including ovaries, uterine horns, body, and vagina [27,28]. Genotyping in Egyptian small ruminants identified Type II (92.8%) and Type III (7.1%) as predominant strains in reproductive tissues, with important public health implications [29]. Experimental vaginal infection in mice results in uterine and placental parasite detection, though fetal involvement remains inconsistent, suggesting the uterus may serve as a persistent reservoir [30]. The clinical significance of uterine persistence is underscored by findings that chronic infection induces uterine atrophy in mice secondary to hypothalamic-pituitary-ovarian axis dysfunction [6]. Administration of 17β-estradiol to infected mice restored uterotropic responses, ruling out direct uterine refractoriness and implicating central hypogonadism as the primary defect [6,7]. This reproductive failure is characterized by interruption of pregnancy, fetal wastage, and infertility, linked to an acquired hypogonadotropic hypogonadism secondary to hypothalamic dysfunction [31]. Beyond laboratory models, *T. gondii* has been detected in uteri of wild red deer across all trimesters and in Hector’s dolphins with suppurative metritis, demonstrating broad species susceptibility [32,33]. Placental infection presents particularly complex diagnostic challenges. *Toxoplasma gondii B1* gene was detected in 86.7% of placental tissues from women with acute toxoplasmosis, despite the absence of anti-*T. gondii* IgM in umbilical cord and neonatal blood, confirming that placental infection can occur without congenital transmission [34]. Similarly, *T. gondii B1* gene was detected in 60% of placental tissues from IgG seropositive, IgM seronegative women, reiterating that the presence of the parasite in the placenta does not always result in congenital toxoplasmosis [35]. This persistent presence establishes a local reservoir for vertical transmission and creates opportunities for chronic inflammatory pathology that may compromise reproductive success even without active fetal infection.

### 2.2. Mechanisms of Female Reproductive Immunopathology

*Toxoplasma gondii* disrupts maternal–fetal immune homeostasis through coordinated dysregulation of multiple decidual cell populations.

#### 2.2.1. Uterine Innate Lymphoid Cells and Natural Killer Cells

Infection reprograms uILCs from a pregnancy-supportive (GATA-3^+^, *RORγt*^+^) to a pro-inflammatory Th1-like (*T-bet*^+^) phenotype, increasing IFN-γ and TNF-α while decreasing IL-5 and IL-17. dNK cells undergo cytotoxic skewing characterized by expansion of *CD56dimCD16*^+^ subset, upregulation of *NKG2D*, and loss of inhibitory receptors *KIR2DL4* and *ILT-2*, enhancing IFN-γ secretion and killing capacity [36,37,38,39,40]. The protective *Tim-3* checkpoint is downregulated, activating *PI3K*/*AKT* and *JAK*-*STAT* pathways and unleashing granzyme B and perforin [41]. *HLA-G* is paradoxically elevated, inducing dNK apoptosis via caspases while potentially facilitating immune evasion [42,43]. Functional deficiency of uterine trNK cells drives adverse pregnancy outcomes, rescued by adoptive transfer [44,45,46]. *PD-1*/*PD-L1* upregulation on *DX5+* NK cells correlates with decreased NK cell proportions [47].

#### 2.2.2. Decidual Macrophages and Dendritic Cells

dMφ polarize from M2 to pro-inflammatory M1 phenotype (↓*CD206*, arginase-1; ↑*iNOS*, *CD80*, *CD86*, TNF-α, IL-12) [48]. *TgROP18* downregulates *CD73* via *LSD1*/*SNAIL1*, reducing *Arg-1* and IL-10 through the adenosine/*A2AR*/*PKA*/*p-CREB*/*C/EBPβ* pathway [49,50,51,52,53,54]. *TgROP18* also suppresses *Gal-9* via *JNK*/*FOXO1*, causing dNK dysfunction through *Gal-9*/*Tim-3* interaction [51,52,53,54,55]. *Trem2* is downregulated, impairing *Trem2*-*Syk*-*PI3K* and *Trem2*-*PPARγ*-*STAT6* signaling [53,56]. Infected decidual dendritic cells produce IL-12, amplifying dNK cytotoxicity [37]. Single-cell RNA-seq reveals 279, 312, and 380 differentially expressed genes in dNK cells, dMφ, and decidual T cells, with 17 immune cell clusters showing proportion changes and 21 genes with altered expression [16]. This coordinated dysregulation across multiple immune cell populations creates a hostile decidual environment incompatible with healthy pregnancy maintenance.

#### 2.2.3. Regulatory T Cells and Myeloid-Derived Suppressor Cells

**Figure 1:**
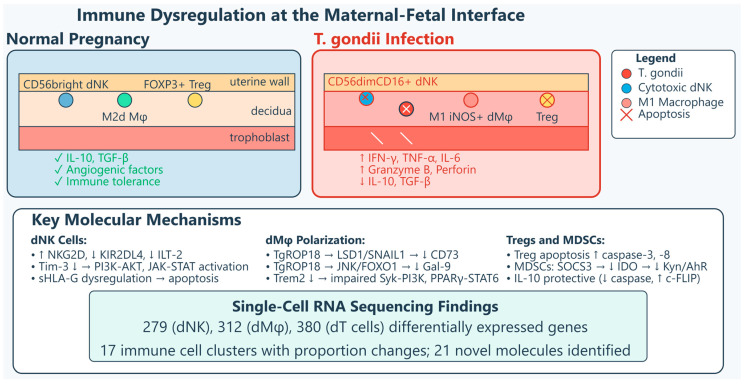
Immune dysregulation at the maternal–fetal interface. *Toxoplasma gondii* disrupts decidual immune homeostasis through coordinated dysregulation of multiple cell populations. In normal pregnancy (left), the decidua contains three pregnancy-supportive immune populations: (1) *CD56bright dNK* cells (light blue circle)—non-cytotoxic, cytokine-secreting natural killer cells that support vascular remodeling; (2) M2 dMφ (green circle)—alternatively activated decidual macrophages with anti-inflammatory, tissue-repair functions; and (3) FOXP3+ Tregs (yellow circle)—regulatory T cells that maintain maternal–fetal tolerance. During *T. gondii* infection (right), all three populations are altered, and the parasite appears directly. The red circle with a white cross represents *T. gondii* tachyzoites replicating in decidual/trophoblast tissue. The dark blue circle labeled *CD56dimCD16+ dNK* represents cytotoxic decidual NK cells that secrete granzyme B and perforin, killing trophoblast cells. The light red/pink circle labeled M1 iNOS+ dMφ represents pro-inflammatory M1 macrophages that produce iNOS, TNF-α, and IL-12, driving local inflammation. The yellow circle with a red “X” represents apoptotic FOXP3+ Tregs (caspase-3/8-mediated), indicating loss of immune suppression. White damage lines (\\) on the trophoblast layer indicate infection-induced tissue injury. Symbols: ↑ = increased expression/abundance (e.g., IFN-γ, TNF-α, granzyme B); ↓ = decreased expression/abundance (e.g., IL-10, TGF-β). → = proceed to. Bottom panel (molecular mechanisms): Parasite effector molecules (e.g., TgROP18) and disrupted host pathways (e.g., LSD1/SNAIL1, JNK/FOXO1, PI3K/AKT, JAK-STAT, Trem2-Syk-PI3K, PPARγ-STAT6, SOCS3-IDO-Kyn/AhR) are shown. The teal box summarizes single-cell RNA-seq findings: 279 (dNK), 312 (dMφ), and 380 (decidual T cells) differentially expressed genes; 17 altered immune cell clusters; 21 novel molecules identified. Legend (upper right): Quick reference for *T. gondii*, cytotoxic dNK, M1 macrophage, and apoptosis symbols.

Tregs undergo apoptosis via *caspase-3* and *caspase-8* upregulation [57,58]. IL-10 reduces Treg apoptosis; rIL-10 treatment downregulates cleaved *caspase-3* and *caspase-8*, while IL-10 deficiency worsens outcomes. *PD-1* expression increases on surviving Tregs, but overall numbers and suppressive capacity diminish [58,59]. MDSCs show reduced IDO expression via *SOCS3*-mediated degradation, reducing TGF-β and IL-10 through the *Kyn*/*AhR*/*SP1* pathway [51,52,53,54,59], Table 1, Figure 1.

#### 2.2.4. Cytokine-Mediated Tissue Damage and Cell Death Pathways

Both female and male reproductive tissues experience cytokine-driven pathology, though with distinct consequences reflecting tissue-specific functions. The decidual cytokine storm—characterized by increased IFN-γ and TNF-α with reciprocal decreases in IL-10 and TGF-β—directly damages placental tissues [61,62]. IFN-γ secreted by hyperactivated dNK cells correlates positively (r = 0.7163) with trophoblast apoptosis via caspase activation, with neutralizing antibodies significantly reducing cell death [40]. A study showed that in human placental trophoblast (BeWo and HTR-8/SVneo) and amniotic (WISH) cells, *T. gondii* decreases mitochondrial membrane potential, increases ROS production, and releases cathepsin B (CatB) into the cytosol [63]. This oxidative and lysosomal stress triggers activation of multiple inflammasome complexes—*NLRP1*, *NLRP3*, *NLRC4*, and *AIM2*—leading to caspase-1-mediated pyroptosis, as evidenced by increased expression of *ASC*, cleaved caspase-1, mature IL-1β, and gasdermin D cleavage. The functional significance was confirmed through rescue experiments: administration of ROS scavengers, CatB inhibitors, or inflammasome-specific siRNA reversed these effects in infected cells. Importantly, adverse pregnancy outcomes were observed in a *T. gondii*-infected pregnant mouse model, correlating with inflammasome activation and pyroptosis induction in placental tissues. This mechanism may play an important role in inducing cell injury in congenital toxoplasmosis, revealing that the parasite triggers a programmed, inflammatory form of cell death rather than passive necrosis [63].

Genetic background significantly influences susceptibility. *C57BL/6* mice (H2b haplotype) exhibit 90% abortion rates versus 50% in resistant *BALB/c* mice, associated with higher systemic TNF-α and decidual inflammatory foci [64,65]. The impaired pregnancy outcome in *C57BL/6* mice is associated with a higher inflammatory response leading to cell apoptosis and necrosis of implantation sites, with this phenomenon not due to inducible nitric oxide synthase expression in the decidua [65]. Studies using congenic mice have confirmed that the H2 haplotype influences pregnancy outcomes, with high IFN-γ and TNF serum levels involved in poor pregnancy outcomes even when parasite detection in the uterus and placenta is low [65]. Furthermore, *FOXP3* expression at the maternal–fetal interface is reduced following infection, with the reduction being larger in susceptible *C57BL/6* mice compared with resistant *BALB/c* mice [62]. IFN-γ receptor knockout mice show 50% less fetal resorption, though spiral artery dilation and placental apoptosis persist, revealing the cytokine’s dual role in protection and pathology [66]. *CCR5* signaling contributes to embryo loss even without detectable parasites in fetoplacental tissues, implicating maternal immune responses rather than direct fetal infection as the primary driver [67].

**Figure 2:**
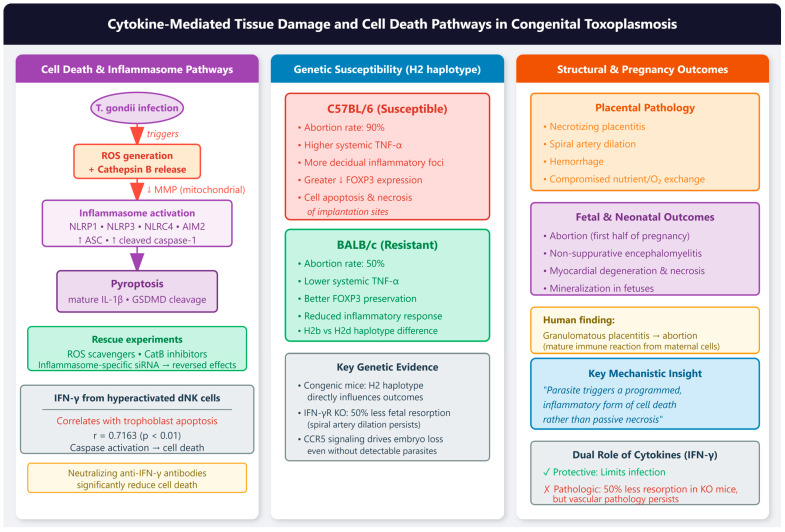
Cytokine-mediated tissue damage and cell death pathways in congenital toxoplasmosis. Left panel (Cell death & inflammasome pathways). Toxoplasmosis triggers reactive oxygen species (ROS) generation and cathepsin B (CatB) release into the cytosol, accompanied by decreased mitochondrial membrane potential (MMP). This oxidative and lysosomal stress activates multiple inflammasome complexes (*NLRP1*, *NLRP3*, *NLRC4*, *AIM2*), leading to *ASC* speck formation, cleaved caspase-1, mature IL-1β, and gasdermin D (*GSDMD*) cleavage—hallmarks of pyroptosis. Rescue experiments using ROS scavengers, CatB inhibitors, or inflammasome-specific siRNA reverse these effects. Separately, IFN-γ secreted by hyperactivated decidual natural killer (dNK) cells correlates positively with trophoblast apoptosis via caspase activation; neutralizing anti-IFN-γ antibodies significantly reduce cell death. Middle panel (Genetic susceptibility). The H2 haplotype strongly influences pregnancy outcomes. Susceptible *C57BL/6* mice (H2^b^) exhibit a 90% abortion rate, higher systemic TNF-α, more decidual inflammatory foci, greater reduction in *FOXP3* expression, and apoptosis/necrosis of implantation sites. Resistant *BALB/c* mice (H2^d^) show a 50% abortion rate with lower inflammation and better *FOXP3* preservation. Congenic mouse studies confirm the H2 haplotype’s direct role. IFN-γ receptor knockout reduces fetal resorption by 50%, but spiral artery dilation persists, revealing the cytokine’s dual role. *CCR5* signaling contributes to embryo loss even without detectable fetal parasites, implicating maternal immunity as the primary driver. Right panel (Structural & pregnancy outcomes). Consequences include necrotizing placentitis, spiral artery dilation, hemorrhage, and compromised nutrient exchange. Fetal outcomes include abortion (first half of pregnancy in humans), non-suppurative encephalomyelitis, myocardial degeneration, necrosis, and mineralization. Human granulomatous placentitis represents a mature maternal immune reaction. A key mechanistic insight is that the parasite triggers programmed, inflammatory cell death (pyroptosis) rather than passive necrosis. IFN-γ plays a dual role: protective against infection but also pathological, as demonstrated by reduced resorption but persistent vascular pathology in knockout models (Purple (boxes colour and text) indicate inflammasome activation and pyroptosis pathways; red denote inflammatory mediators and cellular stress signals; green represent protective or rescue interventions—in the left panel highlighting experimental evidence using ROS scavengers, cathepsin B inhibitors, and inflammasome-specific siRNA that reverse cell death, and in the middle panel denoting the resistant *BALB/c* mouse strain with favorable pregnancy outcomes; blue text indicate genetic susceptibility factors; orange denote structural and clinical outcomes including placental pathology and fetal/neonatal sequelae; yellow highlight key experimental findings and clinical observations such as neutralizing antibody effects and human pathological findings; and gray text present integrative concepts and supporting evidence including the dual role of IFN-γ and mechanistic insights from knockout studies. Italicized text indicates direct quotations from source literature. Abbreviations: ROS, reactive oxygen species; CatB, cathepsin B; ↓MMP, decreased mitochondrial membrane potential; ASC, apoptosis-associated speck-like protein containing a CARD; GSDMD, gasdermin D; dNK, decidual natural killer; KO, knockout; CCR5, C-C chemokine receptor type 5; H2, major histocompatibility complex class II haplotype in mice). ↑ = increased (expression); ↓ = decreased (expression); → = proceed to.

Structural consequences include necrotizing placentitis, spiral artery dilation, and hemorrhage, compromising nutrient and oxygen exchange [66,68,69]. In pregnant sows experimentally infected with *T. gondii* oocysts, necrotizing placentitis was observed with numerous tachyzoites in trophoblast cells lining areolae, along with non-suppurative encephalomyelitis and myocardial degeneration, necrosis, and mineralization in fetuses [69]. In humans, granulomatous placentitis due to toxoplasmosis has been reported as a cause of abortion in the first half of pregnancy, possibly representing a mature immune reaction from maternal immunocompetent cells [70], Figure 2.

### 2.3. Female: Pregnancy Hormones and Parasite Modulation

Pregnancy-associated hormones critically influence *T. gondii* pathogenesis. Monocytes and macrophages are a significant immunologic barrier against *T. gondii* by boosting inflammation. This outcome is highly regulated by signaling pathways such as *MAPK* (*ERK1/2*) and *PI3K* (*AKT*), necessary in cell growth and proliferation. It may be associated with the hormonal receptors’ modulation by *T. gondii* (Estrogen Receptor (ER)-α, *ERβ*, G Protein-coupled ER (*GPER*), and Prolactin Receptor (*PRLR*)). 17β-estradiol also activates *MAPK* and *PI3K*; however, its combined effect in THP-1 monocytes and macrophages, infected with *T. gondii*, has been evaluated. Studies demonstrate that 17β-estradiol performs activation of *ERK1/2* and *AKT* in *T. gondii*-infected macrophages and modulates the expression of hormonal receptors in infected cells: it increases the *PRLR* and *PrgR* in *T. gondii*-infected macrophages and decreases the *PRLR* and *ERα* in *T. gondii*-infected monocytes. As for *GPER*, its expression is abolished by *T. gondii*, and 17β-estradiol cannot restore it. These findings indicate that 17β-estradiol modifies the receptors of *T. gondii*-infected THP1 macrophages and monocytes in an *ERK*/*AKT*-dependent manner [14].

#### 2.3.1. Estradiol

Estradiol promotes Type II and III parasite infection and contributes to pathogenicity through the parasite’s own hydroxysteroid dehydrogenase (*Tg-HSD*), which efficiently transforms estrone to estradiol. *Tg-HSD*-overexpressing parasites show enhanced pathogenicity and upregulated estradiol levels in mice, demonstrating that the parasite utilizes host hormones for its own benefit [71]. The interaction between *Tg-HSD* and the host endocrine system operates through specific, non-conventional pathways rather than direct enzymatic interference with the host Hypothalamic–Pituitary–Gonadal (HPG) axis, as follows: (1) *Tg-HSD* does not directly interfere with the host HPG axis. Instead, the parasite converts host-derived estrone (E1) into estradiol (E2) within its own cytoplasm. The newly synthesized estradiol is subsequently released into the host circulation, where it acts indirectly to feedback-inhibit hypothalamic GnRH pulsatility. This represents an indirect endocrine disruption strategy, exploiting the host’s native feedback loops rather than directly blocking HPG components [71]. (2) *Tg-HSD* is localized specifically to the parasite Endoplasmic Reticulum (ER), as confirmed by co-localization with the *Tg-Der1* ER marker. There is no study on exosomal or vesicular transfer mechanisms; the crosstalk is characterized solely by intracellular conversion of estrone and release of estradiol into the host cytoplasm. The crosstalk is strictly defined by the intracellular conversion of estrone and the passive or active release of the resulting estradiol into the host cytoplasm [71]. (3) The parasite does not weaken or downregulate this pathway. Functional studies demonstrate that *Tg-HSD*-overexpressing parasitesshow significantly enhanced pathogenicity and upregulated serum estradiol levels in mice. This confirms that *T. gondii* actively strengthens (overexpresses) this metabolic pathway to manipulate host hormonal status for its own survival and virulence advantage [71].

Bioinformatics analysis shows that *T. gondii* may have a residual estradiol metabolism-related gene *Tg-HSD*. Functional studies demonstrate that *Tg-HSD* can efficiently transform estrone into estradiol, and *Tg-HSD*-overexpressing parasites show significantly enhanced pathogenicity and upregulation of estradiol levels in mice. These findings indicate that estradiol can promote toxoplasmosis in vitro and in vivo through mechanisms involving the parasite’s own *Tg-HSD* gene [71]. Sex steroids, particularly estrogen, have emerged as critical modulators of host–pathogen interactions [72]. The susceptibility to toxoplasmosis exhibits notable sex differences, with female mice demonstrating higher mortality rates despite better control of initial parasite replication [73]. This review examines the multifaceted relationship between estrogen and *T. gondii*, exploring how estrogen influences infection susceptibility, immune responses, and parasite biology, while also considering how the parasite may manipulate host endocrine systems for its survival and transmission.

##### Dose-Dependent Effects of Estrogen

Early studies established that estrogen exposure significantly influences susceptibility to toxoplasmosis. Pung and Luster (1986) [74] demonstrated that pharmacological concentrations of potent estrogenic compounds, including 17β-estradiol, diethylstilbestrol, and α-dienestrol, increased susceptibility in mice as measured by brain cyst formation. This effect was specific to estrogenic compounds, as weak estrogens and other hormones including 5α-dihydrotestosterone, progesterone, and zearalanol did not alter host resistance. Importantly, the ability of estrogens to increase susceptibility was inhibited by the estrogen antagonist tamoxifen, and restoration of ovariectomized mice with physiological estrogen concentrations had no effect on infection outcomes. These findings indicate that pharmacological, but not physiological, levels of estrogen selectively alter host resistance to *T. gondii* [74]. Subsequent studies confirmed that estrogen administration induces thymic atrophy and involution of peripheral lymphoid tissues, leading to suppression of cell-mediated immunity and overwhelming toxoplasmosis [75,76,77,78]. Hexoestrol-treated mice exhibited almost complete thymic atrophy and increased mortality following infection, suggesting that the cellular immune response is of greater importance than antibody formation in resistance to toxoplasmosis [75,78].

##### Estrogen and Pregnancy Outcomes

The relationship between estrogen and toxoplasmosis during pregnancy is particularly complex. During gestation, estrogen levels progressively increase and the severity of *T. gondii*-mediated adverse pregnancy outcomes is closely related to the timing of primary maternal infection [79]. Infection in early pregnancy is more likely to induce miscarriage in mice than infection in late pregnancy, correlating with inflammation at the maternal–fetal interface. The apoptotic rate of regulatory T cells (Tregs) was higher, and programmed death-1 (*PD-1*) expression on Tregs was lower in early pregnancy compared to late pregnancy [79]. Importantly, estrogen appeared to protect Tregs against apoptosis and upregulate *PD-1* expression in a dose-dependent manner through estrogen receptor α (*ERα*). Simulated mid-pregnancy levels of estradiol alleviated infection-induced apoptosis of Tregs and potentiated *PD-1* expression in nonpregnant mice. Thus, estrogen helps maintain immune balance and improve pregnancy outcomes during toxoplasmosis [79]. Similarly, *Inonotus obliquus* polysaccharide (IOP) protected against adverse pregnancy outcomes by restoring progesterone and estriol levels and regulating the Th17/Treg balance [80]. However, conflicting evidence exists regarding estrogen’s effects during pregnancy. Estradiol promoted infection with Type II (Pru) and Type III (VEG) strains, contributing to pathogenicity in mice [71]. This discrepancy may reflect differences in experimental models, parasite strains, and the distinction between physiological and pharmacological estrogen concentrations.

##### Sex Differences in Infection Outcomes

Sex differences in toxoplasmosis outcomes are well documented. Female mice are more susceptible to acute infection, with higher mortality rates and greater weight loss compared to males. Paradoxically, female mice exhibited lower parasite burdens during acute infection, suggesting that increased mortality results from an exaggerated immune response rather than failure to control parasite replication. Female mice produced significantly higher levels of monocyte chemoattractant protein-1 (MCP-1), interferon-γ (IFN-γ), and tumor necrosis factor-α (TNF-α) than males, indicating a stronger but potentially detrimental inflammatory response [73]. Similarly, female mice demonstrated higher brain cyst burden and more severe pathological reactions than males in chronic infection, while IL-12 serum levels were significantly higher in male mice. Treatment responses also differed by sex, with combined testosterone/atovaquone or testosterone/spiramycin/metronidazole regimens being most effective in females, while spiramycin/metronidazole alone was superior in males [81].

##### Estrogen Receptor Expression in Toxoplasmosis

The effects of estrogen on immune cells during toxoplasmosis are mediated through estrogen receptors. Toxoplasmosis induces the expression of *ERα* and *ERβ* in THP-1 monocytes while decreasing prolactin receptor (*PRLR*) expression. Estradiol treatment diminished *PRLR* expression, while progesterone decreased *PRLR* and *ERβ* but increased *ERα* expression. These findings indicate that hormones can modulate the expression of other hormonal receptors during infection, potentially influencing immune responses [82]. Further mechanistic studies revealed that 17β-estradiol modulates hormonal receptor expression in *T. gondii*-infected macrophages and monocytes through *AKT* and *ERK*-dependent signaling pathways. Estradiol increased *PRLR* and progesterone receptor expression in infected macrophages while decreasing *PRLR* and *ERα* in infected monocytes. The G protein-coupled estrogen receptor (*GPER*) was abolished by infection, and estradiol could not restore its expression. Blockade of *ERK* and *AKT* pathways modified hormonal receptor expression, establishing these signaling cascades as critical mediators of estrogen’s effects during infection [14].

##### Estrogen and Cytokine Production

Estrogen significantly influences cytokine production during toxoplasmosis. Estradiol decreased the secretion of proinflammatory cytokines IL-12 and IL-1β while increasing anti-inflammatory IL-10 production in infected THP-1 cells. Progesterone similarly increased IL-10 but reduced IL-4 and IL-13. These findings suggest that estrogen may promote an anti-inflammatory environment that could favor parasite survival [82]. The balance between proinflammatory and anti-inflammatory responses is critical for controlling toxoplasmosis while preventing immunopathology. The exaggerated inflammatory response in female mice, characterized by elevated MCP-1, IFN-γ, and TNF-α, contributed to increased morbidity and mortality despite better control of parasite replication. Interestingly, MCP-1 levels were not directly affected by physiological concentrations of estrogen or testosterone in vitro, suggesting that sex differences in immune responses are mediated through indirect mechanisms involving other factors [73].

##### Calcium Signaling and Parasite Motility

Emerging evidence indicates that estrogen directly influences parasite biology. Estradiol and progesterone induce cytosolic calcium fluxes in *T. gondii* tachyzoites. Estradiol-induced calcium fluxes required entry into the parasite cytoplasm and involved cGMP-dependent protein kinase G (*PKG*) and phosphoinositide-phospholipase C (*PI-PLC*). Cytosolic calcium mobilized by estradiol was primarily derived from acidic organelles and resulted in increased microneme protein 2 (*MIC2*) secretion, enhanced gliding motility, and accelerated parasite egress. In contrast, progesterone mobilized calcium from neutral stores, reduced *MIC2* secretion, and inhibited gliding motility, suggesting opposing effects of these hormones on parasite behavior [83]. These findings are consistent with the established role of calcium signaling in *T. gondii* egress. A positive feedback loop involving cAMP and lipid metabolism links calcium and cyclic nucleotide signaling during egress. Understanding how estrogen modulates these signaling pathways may provide insights into parasite biology and identify new therapeutic targets [84].

##### Parasite Estrogen Metabolism

Remarkably, *T. gondii* appears capable of metabolizing estrogens. *Tg-HSD* gene in the parasite genome was identified, and it efficiently converts estrone to estradiol. Overexpression of *Tg-HSD* in parasites significantly enhanced pathogenicity and increased estradiol levels in infected mice. The ability of *T. gondii* to metabolize estrogens suggests that the parasite may actively manipulate its hormonal environment to promote survival and replication [71].

##### Host–Parasite Endocrine Interactions

The bidirectional interactions between *T. gondii* and host endocrine systems extend beyond estrogen. There is evidence that sex steroids mediate bidirectional interactions between hosts and microbes. Testosterone, estradiol, and progesterone affect immune cell function, while microbes can use these hormones and manipulate sex steroid receptor signaling mechanisms to increase survival and replication. This arms race between hosts and microbes has driven the evolution of sophisticated mechanisms for utilizing sex steroid hormone signaling [72]. Chronic toxoplasmosis can also alter host endocrine function. Chronic murine toxoplasmosis causes ovarian dysfunction, leading to uterine atrophy. This effect was not due to uterine refractoriness to estrogen, as infected mice showed normal uterotropic responses to exogenous estradiol, but rather resulted from pituitary gonadotropin insufficiency. The authors hypothesized that peripherally released cytokines reach the hypothalamus, inhibiting pulsatile gonadotropin-releasing hormone release and impairing the pituitary-ovarian axis [6,7]. Similarly, *T. gondii* infection in pregnant goats disrupted endocrine fetal-placental function, with decreased oestrone sulphate levels and increased prostaglandin F2α metabolites preceding abortion [85].

##### Therapeutic Implications

The discovery that tamoxifen, a selective estrogen receptor modulator (SERM), exhibits potent antiparasitic activity against *T. gondii* has opened new therapeutic possibilities. A screen of 1120 compounds identified tamoxifen as a potent inhibitor of *T. gondii* replication, with a 50% inhibitory concentration below 5 μM. Although *T. gondii* can activate the estrogen receptor, tamoxifen inhibits parasite growth independently of this transcription factor. Instead, tamoxifen induces autophagy, stimulating recruitment of the autophagy marker LC3-GFP onto the parasitophorous vacuole membrane and leading to time-dependent elimination of intracellular parasites [86]. The antimicrobial properties of SERMs are noteworthy, with emerging evidence for their efficacy against normal and resistant microbial strains through various mechanisms. The repurposing of SERMs as broad-spectrum antimicrobial agents, alone or in combination with existing drugs, represents a promising approach for treating toxoplasmosis and other infections [87]. Combining hormone modulators with conventional antiparasitic drugs may enhance therapeutic efficacy. Combined therapy with testosterone/atovaquone or testosterone/spiramycin/metronidazole significantly reduced cyst numbers in female mice, while spiramycin/metronidazole alone was most effective in males. These findings highlight the importance of sex-specific treatment strategies for chronic toxoplasmosis [81]. S-methylcysteine, a garlic-derived compound with antioxidant and anti-inflammatory properties, combined with spiramycin, significantly mitigated hormonal disruptions and promoted tissue recovery in *T. gondii*-infected rats. This combination approach, targeting both the parasite and host endocrine dysfunction, may offer improved outcomes for toxoplasmosis during pregnancy [88].

Despite promising preclinical findings, several challenges remain. The dose-dependent effects of estrogen on infection outcomes suggest that careful consideration of hormonal status is necessary when designing treatment protocols. The paradoxical finding that physiological estrogen levels may protect against adverse pregnancy outcomes while pharmacological levels increase susceptibility underscores the importance of dosing in therapeutic applications [79]. Furthermore, the complex signaling networks involved in *T. gondii* biology presents both challenges and opportunities. The critical role of phosphodiesterases in cyclic nucleotide turnover across apicomplexan parasites has been reviewed, highlighting their potential as therapeutic targets. Understanding how estrogen modulates these signaling pathways could identify novel intervention points [74,89].

Several key questions remain regarding the molecular mechanisms underlying estrogen–*T. gondii* interactions. The signaling pathways identified (*AKT* and *ERK*) and (*PKG* and *PI-PLC*) require further elucidation [14,83,90]. Additionally, the role of estrogen in modulating autophagy and xenophagy, as suggested by tamoxifen studies [86], warrants investigation using physiological estrogen concentrations. The association between toxoplasmosis and altered offspring sex ratios suggests that toxoplasmosis may influence human reproductive endocrinology [91]. It is hypothesized that women infected with *T. gondii* may have elevated estrogen levels, potentially explaining reports of higher offspring sex ratios, longer gestations, and behavioral traits. Validation of these hypotheses in human populations could have important public health implications [91]. The identification of tamoxifen as an antiparasitic agent [86,87] supports further investigation of SERMs and other hormone modulators for toxoplasmosis treatment. The development of parasite-specific inhibitors targeting *Tg-HSD* [71] or phosphodiesterases [89] may provide more selective therapeutic options. Additionally, understanding sex differences in treatment responses [81] will be essential for optimizing clinical protocols. The temporal dynamics of estradiol during pregnancy modulate disease severity. Early pregnancy infection, when estradiol levels are lower, induces higher Treg apoptosis and lower *PD-1* expression than late pregnancy infection. Estradiol protects Tregs against apoptosis and upregulates *PD-1* in a dose-dependent manner through *ERα*, explaining the discrepancy in outcomes by gestational age [79]. As the level of E2 in mouse serum gradually increases with the development of pregnancy, E2 may contribute to the discrepancy of Tregs at different stages of pregnancy. E2 in vitro can protect Tregs against apoptosis and upregulate the expression of *PD-1* on Tregs in a dose-dependent manner through *ERα*. Likewise, the simulated mid-pregnancy level of E2 in nonpregnant mice also alleviates toxoplasmosis-induced apoptosis of Tregs and potentiates *PD-1* expression on Tregs. Therefore, in the pathogenesis of *T. gondii*-induced abnormal pregnancy, E2 helps maintain immune balance and improve pregnancy outcome through regulating Tregs [79].

**Figure 3:**
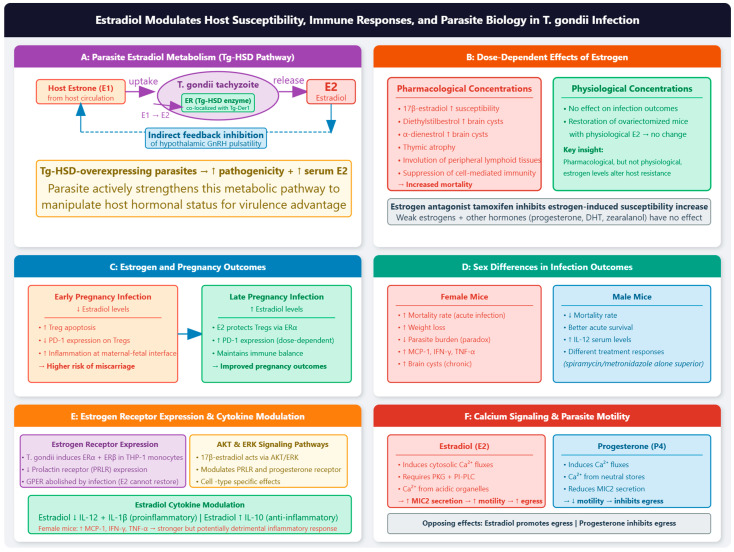
Estradiol modulates host susceptibility, immune responses, and parasite biology in toxoplasmosis. (**A**) Parasite estradiol metabolism (*Tg-HSD* pathway). *Toxoplasma gondii* possesses a hydroxysteroid dehydrogenase gene (*Tg-HSD*) localized to the parasite endoplasmic reticulum (ER), confirmed by co-localization with the *Tg-Der1* ER marker. *Tg-HSD* efficiently converts host-derived estrone (E1) into estradiol (E2) within the parasite cytoplasm. The newly synthesized estradiol is released into host circulation, where it acts indirectly to feedback-inhibit hypothalamic GnRH pulsatility. This represents an indirect endocrine disruption strategy exploiting the host’s native feedback loops rather than directly blocking HPG axis components. *Tg-HSD*-overexpressing parasites show enhanced pathogenicity and upregulated serum estradiol levels in mice, demonstrating that the parasite actively strengthens this metabolic pathway to manipulate host hormonal status for virulence advantage. (**B**) Dose-dependent effects of estrogen. Pharmacological concentrations of potent estrogenic compounds (17β-estradiol, diethylstilbestrol, α-dienestrol) increase host susceptibility to *T. gondii* as measured by brain cyst formation. These compounds induce thymic atrophy, involution of peripheral lymphoid tissues, and suppression of cell-mediated immunity, leading to increased mortality. Weak estrogens and other hormones (5α-dihydrotestosterone, progesterone, zearalanol) do not alter host resistance. The estrogen antagonist tamoxifen inhibits the estrogen-induced increase in susceptibility. Restoration of ovariectomized mice with physiological estrogen concentrations has no effect on infection outcomes, indicating that pharmacological, but not physiological, estrogen levels selectively alter host resistance. (**C**) Estrogen and pregnancy outcomes. During gestation, estrogen levels progressively increase. Infection in early pregnancy (low estradiol) induces higher regulatory T cell (Treg) apoptosis, lower *PD-1* expression on Tregs, and increased inflammation at the maternal–fetal interface, correlating with a higher risk of miscarriage. Infection in late pregnancy (high estradiol) shows protective effects: estradiol protects Tregs against apoptosis and upregulates *PD-1* expression in a dose-dependent manner through estrogen receptor α (*ERα*), helping maintain immune balance and improve pregnancy outcomes. Simulated mid-pregnancy levels of estradiol in nonpregnant mice alleviate infection-induced Treg apoptosis and potentiate *PD-1* expression. (**D**) Sex differences in infection outcomes. Female mice are more susceptible to acute infection, with higher mortality rates and greater weight loss than males, yet exhibit lower parasite burdens during acute infection. This paradox suggests that increased mortality results from an exaggerated immune response rather than failure to control parasite replication. Female mice produce significantly higher levels of MCP-1, IFN-γ, and TNF-α than males, indicating a stronger but potentially detrimental inflammatory response. In chronic infection, females demonstrate higher brain cyst burden and more severe pathological reactions than males, while IL-12 serum levels are significantly higher in males. Treatment responses also differ by sex, with combined testosterone/atovaquone or testosterone/spiramycin/metronidazole regimens being most effective in females, while spiramycin/metronidazole alone is superior in males. (**E**) Estrogen receptor expression and cytokine modulation. *Toxoplasma gondii* infection induces *ERα* and *ERβ* expression in THP-1 monocytes while decreasing prolactin receptor (*PRLR*) expression. The G protein-coupled estrogen receptor (*GPER*) is abolished by infection, and estradiol cannot restore its expression. 17β-Estradiol modulates hormonal receptor expression through *AKT* and *ERK*-dependent signaling pathways, with cell type-specific effects in macrophages versus monocytes. Estradiol decreases the secretion of proinflammatory cytokines IL-12 and IL-1β while increasing anti-inflammatory IL-10 production in infected cells, suggesting that estrogen may promote an anti-inflammatory environment favoring parasite survival. However, female mice exhibit elevated MCP-1, IFN-γ, and TNF-α, contributing to increased morbidity despite better control of parasite replication. (**F**) Calcium signaling and parasite motility. Estradiol induces cytosolic calcium fluxes in *T. gondii* tachyzoites, requiring entry into the parasite cytoplasm and involving cGMP-dependent protein kinase G (*PKG*) and phosphoinositide-phospholipase C (*PI-PLC*). Cytosolic calcium mobilized by estradiol is primarily derived from acidic organelles and results in increased microneme protein 2 (*MIC2*) secretion, enhanced gliding motility, and accelerated parasite egress. Progesterone has opposing effects: it mobilizes calcium from neutral stores, reduces *MIC2* secretion, and inhibits gliding motility. These opposing effects demonstrate that host hormones directly modulate parasite behavior through calcium-dependent signaling pathways (Purple arrows (**A**) indicate estradiol (E2) metabolism, trafficking, and conversion (E1 → E2) within the parasite endoplasmic reticulum. Blue dashed arrows (**A**) represent indirect feedback inhibition of the hypothalamic–pituitary–gonadal (HPG) axis. Blue arrows between panels (**C**) indicate comparison between early and late pregnancy. Color coding: Red/pink boxes denote detrimental outcomes (increased susceptibility, pathology, miscarriage, female sex, pro-inflammatory effects); blue boxes denote male-specific effects or protective mechanisms; green/teal boxes denote physiological concentrations, protective outcomes, or improved pregnancy outcomes; orange/purple boxes denote mechanistic pathways (dose effects, estrogen receptor signaling, AKT/ERK pathways); and yellow boxes highlight key findings or therapeutic insights). ↑ = increased (expression); ↓ = decreased (expression); → = proceed to.

Exposure to pharmacological concentrations of potent estrogenic compounds, including 17β-estradiol, diethylstilbestrol, and α-dienestrol, increases the susceptibility of mice to *T. gondii* as measured by brain cyst formation. Compounds with weak estrogenic activity or other hormonal activity, including 5α-dihydrotestosterone, progesterone, and zearalanol, do not alter host resistance to infection. The ability of estrogens to alter susceptibility is inhibited by the estrogen antagonist tamoxifen. The restoration of ovariectomized mice with normal physiological concentrations of estrogen has no effect on subsequent infection with *T. gondii*, indicating that pharmacological, but not physiological, levels of estrogen selectively alter host resistance to *T. gondii*, possibly through hormonal events [74], Figure 3.

#### 2.3.2. Progesterone

Progesterone is a critical hormone for the establishment and maintenance of pregnancy. During gestation, progesterone levels progressively increase, peaking in the third trimester [92]. Given the temporal association between rising progesterone levels and increased susceptibility to toxoplasmosis during pregnancy, it has long been hypothesized that this hormone may modulate host–parasite interactions [93]. However, the relationship between progesterone and *T. gondii* has proven remarkably complex, with evidence for both host-mediated and direct parasite-directed effects.

##### Direct Effects of Progesterone on *T. gondii*

A paradigm-shifting discovery demonstrated that progesterone directly inhibits the invasion and proliferation of *T. gondii* tachyzoites, inducing abnormal cytoskeletal daughter budding and subsequent autophagy in vitro. This finding contradicts the long-held assumption that progesterone uniformly benefits the parasite. A *T. gondii* progesterone membrane receptor protein (*TgPGRMC*) localized to the mitochondrion was identified that mediates these effects. Critically, knockout of the *PGRMC* gene conferred resistance to progesterone’s inhibitory effects, establishing *TgPGRMC* as an essential link between the parasite and progesterone signaling [94]. The existence of a parasite-encoded steroid hormone receptor is consistent with the broader concept that microbes have evolved to utilize host hormonal signaling mechanisms for survival and replication [72]. Further characterization of the steroidogenic capacity of *T. gondii* demonstrated that the parasite possesses a functional type I cytochrome *P450* enzyme (*TgCYP450mt*) and a membrane-associated progesterone receptor (*TgMAPR*) that localize to the mitochondrion. Genetic ablation of *CYP450mt* impairs parasite growth, and both *TgCYP450mt* and *TgMAPR* are required for full virulence in mice. Metabolomics studies revealed that these proteins are involved in steroid synthesis, with the parasite producing anti-inflammatory hydroxypregnenolone species, deoxycorticosterone, and dehydroepiandrosterone [19].

The effects of progesterone on parasite motility are mediated through calcium signaling. Progesterone induces cytosolic calcium fluxes in *T. gondii* tachyzoites, with calcium mobilized primarily from neutral stores. In contrast to estradiol, which increases microneme protein 2 (*MIC2*) secretion and promotes gliding motility, progesterone reduces *MIC2* secretion and inhibits parasite gliding motility, potentially decreasing pathogenicity [83,90]. This differential effect highlights the opposing actions of estradiol and progesterone on parasite behavior.

The interaction between progesterone and *TgGST2*, a parasite glutathione S-transferase involved in vesicle trafficking and sterol metabolism, was investigated. Progesterone bound to *TgGST2* and inhibited its enzymatic activity in a concentration-dependent manner. Upon progesterone treatment, *TgGST2* localization changed from the Golgi and vesicles to hollow circles, leading to abnormal localization of the molecular transporter Sortilin (*VPS10*) and microneme proteins (*M2AP* and *MIC2*), ultimately affecting parasite life activities. Notably, estradiol did not produce similar effects, indicating the specificity of the progesterone–*TgGST2* interaction [95].

Dehydroepiandrosterone (DHEA), a precursor steroid that can be converted to progesterone, exhibits toxoplasmicidal effects on extracellular tachyzoites. DHEA treatment altered parasite cytoskeletal structures, leading to loss of organelle organization and cellular shape. Notably, in the presence of DHEA, a *TgPGRMC* with a cytochrome b5 family heme/steroid binding domain was expressed, while proteins essential for motility and virulence were reduced. In vivo, DHEA treatment reduced parasitic load in male but not female mice, suggesting sex-dependent effects [96].

##### Progesterone Modulation of Host Immune Responses

Progesterone exerts profound effects on the host immune response to *T. gondii*. Progesterone treatment of THP-1 monocytes infected with *T. gondii* increased infection rates while reducing proinflammatory cytokines IL-4 and IL-13. Both progesterone and estradiol increased anti-inflammatory IL-10 production, suggesting that these hormones promote an anti-inflammatory environment that may facilitate parasite survival [82]. The mechanisms by which progesterone modulates macrophage function following Toll-like receptor-4 (*TLR-4*) activation were systematically investigated. Using the synthetic progesterone receptor-specific agonist norgestrel and the glucocorticoid receptor agonist dexamethasone, they demonstrated that progesterone-mediated suppression of nitric oxide (NO) production occurs through the glucocorticoid receptor, while suppression of interleukin-12 (IL-12) production can be mediated through either receptor. Importantly, while progesterone reduced NO-mediated killing of *Leishmania donovani*, it had little effect on *T. gondii* growth in activated macrophages, suggesting that progesterone-mediated increased susceptibility during pregnancy is more likely related to IL-12 downregulation and suppression of type-1 immune responses [97].

T cell immunity to *T. gondii* in pregnant women with primary toxoplasmosis was investigated, a question previously unexplored in humans. T cells from infected pregnant women proliferated in response to *T. gondii* tachyzoites, excretory-secretory antigens, and recombinant surface antigen-1 (rSAG-1), displaying a Th1 or Th0 cytokine profile with overexpression of IFN-γ. Importantly, this Th1-biased response remained unchanged upon in vitro exposure to progesterone at concentrations approximating those at the maternal–fetal interface, suggesting that progesterone does not directly suppress established anti-*T. gondii* T cell responses [98].

The expression of progesterone receptors is dynamically regulated during infection. Toxoplasmosis induces the expression of estrogen receptors *ERα* and *ERβ* while lowering prolactin receptor (*PRLR*) expression. Progesterone treatment decreased *PRLR* and *ERβ* expression but increased *ERα* expression, indicating complex cross-regulation among hormone receptors during infection [82]. Progesterone combined with estradiol decreased the number of intracellular parasites in infected neurons at 48 h post-treatment. Using specific agonists and antagonists, they showed that the effects of progesterone on neuronal infection are mediated through classical progesterone receptors, as the antagonist mifepristone modulated progesterone’s effects [99].

##### Synthetic Progestins and Toxoplasmosis

The first study examining the effects of levonorgestrel (LNG), a synthetic progestin used for contraception in stray animals, on *T. gondii* susceptibility was conducted. Using both in vivo and in vitro models, they demonstrated that LNG increased the risk of toxoplasmosis, an effect associated with downregulation of IFN-γ levels rather than alterations in host sex hormone levels. Concurrently, LNG enhanced the expression of its ligand, the progesterone receptor (*PGR*), on host cells. Critically, the promotional effect of LNG on infection was attenuated when the *PGR* gene was knocked down, establishing a causal role for *PGR* upregulation [51,52,53,54]. These findings have important implications for the widespread use of LNG as a contraceptive in stray dog and cat populations, which serve as intermediate and definitive hosts for *T. gondii*, respectively. The effects of progesterone, norgestrel (a synthetic progesterone-receptor-specific agonist), and dexamethasone on macrophage function were compared. While progesterone and dexamethasone significantly reduced NO production following LPS stimulation, norgestrel did not, indicating that this effect is mediated through the glucocorticoid receptor rather than the progesterone receptor. However, all three steroids downregulated IL-12 production, demonstrating that progesterone receptor ligation alone is sufficient to modulate this aspect of the immune response [97]. The available evidence suggests that natural progesterone and synthetic progestins may have divergent effects on toxoplasmosis. Direct antiparasitic effects of natural progesterone through *TgPGRMC* [94] were domonstrated, while levonorgestrel increased infection risk through host PGR upregulation and IFN-γ suppression [51,52,53,54]. These differences may reflect distinct mechanisms of action, with natural progesterone acting directly on the parasite and synthetic progestins primarily affecting host immunity. Alternatively, the concentrations used and experimental models may account for some discrepancies.

##### Effects of Toxoplasmosis on Progesterone Homeostasis

*Toxoplasma gondii* infection in pregnant *BALB/c* mice reduced serum progesterone levels and induced adverse effects at the maternal–fetal interface, including inflammation, necrosis, and fetal resorption. Infected pregnant mice exhibited clinical signs of infection, and alterations in immune spleen cells were dependent on the day of pregnancy [100]. Similarly, *T. gondii* infection in pregnant goats caused all inoculated animals aborted or delivered stillborn or weak kids 54–73 days after inoculation. Plasma progesterone levels showed a slight decrease after inoculation, and the pattern of progesterone changes around abortion resembled that around normal parturition, but with elevated prostaglandin F2α metabolites. Oestrone sulphate levels failed to increase before abortion, indicating disturbed endocrine fetal-placental function [85]. Similar findings were reported in pregnant ewes, with infected animals showing decreased progesterone and oestrone sulphate levels following an initial increase in prostaglandin metabolite [101].

A follow-up study of 52 pregnant women was conducted to determine associations between serum hormone levels and anti-*T. gondii* antibody kinetics. The prevalence of anti-*T. gondii* IgG antibodies was 26.92% in the first and second trimesters, increasing to 32.7% in the third trimester. In seropositive women, progesterone increased significantly (*p* < 0.039) in the third trimester, while 17β-estradiol increased in the second and third trimesters and prolactin increased in the second trimester (*p* < 0.021). These findings indicate that progesterone and other hormones are associated with toxoplasmosis during pregnancy, though the direction of causality remains to be determined [92]. *Toxoplasma gondii*-infected human villous explants increased secretion of estradiol, progesterone, and HCG+β. Treatment with azithromycin or the standard combination of pyrimethamine, sulfadiazine, and folinic acid reduced both parasite load and hormone secretion, suggesting that hormonal dysregulation is a consequence of infection rather than a pre-existing condition [102].

The effects of toxoplasmosis on aversive behaviors in female rats were investigated, finding that infected females lost their natural preference for rabbit urine over bobcat urine and spent more time investigating social novelty, indicating reduced risk aversion and increased exploratory behavior. Notably, infected rats had elevated levels of circulating progesterone, a known anxiolytic, and the behavioral effects were influenced by the estrus cycle. Uninfected rats preferred rabbit urine throughout the cycle except at estrus and metestrus, while infected rats lost this preference at every stage except estrus [103]. However, the loss of predator aversion in female rats after infection was not dependent on ovarian steroids, suggesting alternative mechanisms [104].

##### Progesterone and Congenital Toxoplasmosis

The relationship between foodborne infections during pregnancy was reviewed, noting that escalated production of progesterone during pregnancy leads to down-regulation of cellular (cell-mediated) immune functions. Many intracellular pathogens, including *T. gondii*, are controlled by cell-mediated immunity, and pregnancy-induced decreases in these functions increase susceptibility. *Toxoplasma gondii* and *Listeria monocytogenes* are identified as the most important foodborne pathogens in pregnancy in the United States, capable of inducing death or grave disease in the fetus and newborn [105]. Global miRNA expression profiling of domestic cat livers following acute toxoplasmosis and identified 82 differentially expressed miRNAs. Kyoto Encyclopedia of Genes and Genomes analysis revealed that predicted gene targets were involved in multiple pathways, including the progesterone-mediated oocyte maturation pathway, suggesting that infection may influence progesterone signaling at the molecular level [106]. IOP protected against adverse pregnancy outcomes caused by toxoplasmosis in mice. IOP significantly reduced abortion rates, inhibited the decrease in progesterone and estriol levels, and restored the balance of Th17/Treg cells through the *TLR4/NF-κB* pathway [80]. Similarly, S-methylcysteine combined with spiramycin mitigated hormonal disruptions (including progesterone) and promoted tissue recovery in infected rats, suggesting that therapies targeting both the parasite and host endocrine function may be beneficial [88].

##### Conflicting Evidence and Knowledge Gaps

Several studies have reported conflicting findings regarding progesterone’s effects on *T. gondii*. Progesterone failed to modulate *T. gondii* replication in RAW 264.7 murine macrophages, even though it inhibited NO production induced by IFN-γ/LPS. The authors concluded that progesterone has no direct effect on macrophage response to the parasite and that the real effects of sex steroids in toxoplasmosis likely involve broader immune mechanisms [107]. The effects of testosterone and progesterone on *T. gondii* propagation in glioblastoma cells were investigated, finding that testosterone significantly increased infection while progesterone had no significant effects. This contrasts with the direct inhibitory effects reported by Wu et al. (2022) and may reflect differences in cell types, parasite strains, or experimental conditions [94,108]. A major challenge in understanding progesterone–*T. gondii* interactions is distinguishing direct effects on the parasite from indirect effects mediated through host immunity. The discovery of *TgPGRMC* [94] and the steroidogenic capacity of the parasite [19] provides compelling evidence for direct parasite–hormone interactions. However, the immune-modulatory effects of progesterone, including suppression of IL-12 and promotion of IL-10 [82,97], likely contribute to increased susceptibility in vivo. The effects of progesterone may be concentration-dependent. Progesterone, unlike estrogens, did not alter host resistance to infection when administered at pharmacological concentrations [74]. However, this study used pharmacological rather than physiological concentrations, and the effects of physiological progesterone levels during pregnancy may differ. Direct antiparasitic effects were found using progesterone concentrations relevant to pregnancy, suggesting that the discrepancy may relate to experimental conditions [94].

##### Clinical and Public Health Implications

Understanding the complex role of progesterone in toxoplasmosis has important implications for managing toxoplasmosis during pregnancy. While progesterone is essential for pregnancy maintenance and is commonly supplemented in cases of luteal phase deficiency or threatened abortion [109], its potential effects on toxoplasmosis should be considered. The finding that progesterone has direct antiparasitic effects is reassuring [94], but the immunosuppressive effects of pregnancy, mediated in part by progesterone, remain a concern [105]. Important public health concerns were raised regarding the use of levonorgestrel as a contraceptive in stray dogs and cats. Given that these animals play critical roles in the transmission of *T. gondii* (cats as definitive hosts, dogs as intermediate hosts), the finding that LNG increases infection risk has implications for both animal welfare and public health. Alternative contraceptive strategies may need to be considered, or LNG use should be accompanied by monitoring for toxoplasmosis [51,52,53,54]. The identification of *TgPGRMC* as a mediator of progesterone’s antiparasitic effects [94] and the steroidogenic capacity of the parasite [19] suggest new therapeutic targets. Drugs that modulate progesterone receptor signaling or interfere with parasite steroid metabolism could be developed as novel anti-*T. gondii* agents. Additionally, the protective effects of IOP [80] and S-methylcysteine [88] on progesterone levels and pregnancy outcomes suggest that adjunctive therapies targeting hormone homeostasis may improve clinical outcomes.

**Figure 4:**
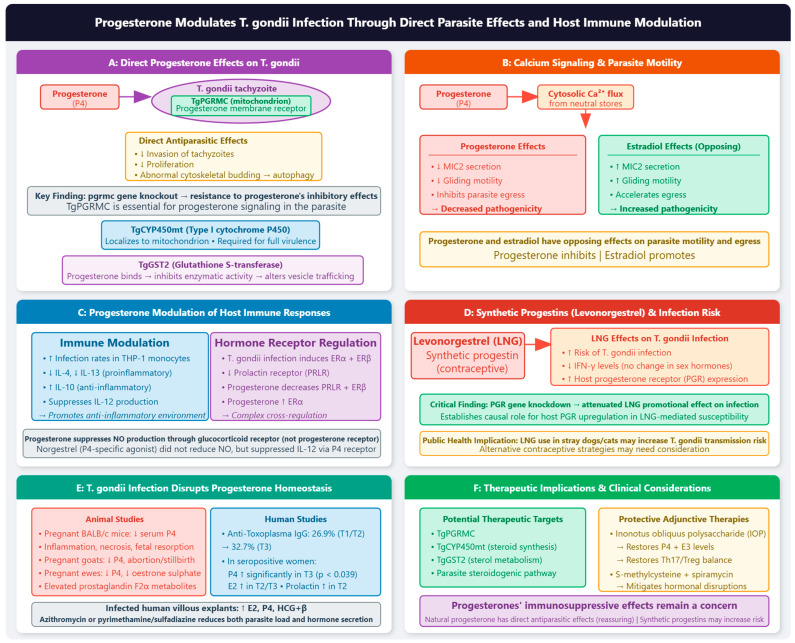
Progesterone modulates toxoplasmosis through direct parasite effects and host immune modulation. (**A**) Direct progesterone effects on *T. gondii*. Progesterone directly inhibits the invasion and proliferation of *T. gondii* tachyzoites, inducing abnormal cytoskeletal daughter budding and subsequent autophagy. These effects are mediated by a parasite-encoded progesterone membrane receptor protein (*TgPGRMC*) localized to the mitochondrion. *TgPGRMC* gene knockout confers resistance to progesterone’s inhibitory effects, establishing *TgPGRMC* as an essential link between the parasite and progesterone signaling. The parasite also possesses a functional type I cytochrome *P450* enzyme (*TgCYP450mt*) and a membrane-associated progesterone receptor (*TgMAPR*) that localize to the mitochondrion; both are required for full virulence in mice. Additionally, progesterone binds to *TgGST2* (a parasite glutathione S-transferase) and inhibits its enzymatic activity in a concentration-dependent manner, leading to abnormal localization of Sortilin (*VPS10*) and microneme proteins (*M2AP* and *MIC2*), ultimately affecting parasite life activities. Estradiol does not produce similar effects, indicating the specificity of the progesterone–*TgGST2* interaction. (**B**) Calcium signaling and parasite motility. Progesterone induces cytosolic calcium fluxes in *T. gondii* tachyzoites, with calcium mobilized primarily from neutral stores. In contrast to estradiol, which increases microneme protein 2 (*MIC2*) secretion and promotes gliding motility, progesterone reduces *MIC2* secretion and inhibits parasite gliding motility, potentially decreasing pathogenicity. (**C**) Progesterone modulation of host immune responses. Progesterone exerts profound effects on the host immune response to *T. gondii*. Progesterone treatment of infected THP-1 monocytes increases infection rates while reducing proinflammatory cytokines IL-4 and IL-13. Both progesterone and estradiol increase anti-inflammatory IL-10 production, suggesting that these hormones promote an anti-inflammatory environment that may facilitate parasite survival. Progesterone-mediated suppression of nitric oxide (NO) production occurs through the glucocorticoid receptor, while suppression of IL-12 can be mediated through either the glucocorticoid or progesterone receptor. Regarding hormone receptor regulation, toxoplasmosis induces *ERα* and *ERβ* expression while lowering prolactin receptor (*PRLR*) expression. Progesterone treatment decreases *PRLR* and *ERβ* expression but increases *ERα* expression, indicating complex cross-regulation among hormone receptors during infection. (**D**) Synthetic progestins (levonorgestrel) and infection risk. Levonorgestrel (LNG) increases the risk of toxoplasmosis. This effect is associated with downregulation of IFN-γ levels (rather than alterations in host sex hormone levels) and enhanced expression of the progesterone receptor (*PGR*) on host cells. Critically, *PGR* gene knockdown attenuates the promotional effect of LNG on infection, establishing a causal role for host *PGR* upregulation. These findings have important public health implications, as LNG use in stray dog and cat populations (which serve as intermediate and definitive hosts for *T. gondii*, respectively) may increase parasite transmission risk. Natural progesterone and synthetic progestins may have divergent effects: natural progesterone exhibits direct antiparasitic effects through *TgPGRMC*, while synthetic progestins primarily affect host immunity. (**E**) Toxoplasmosis disrupts progesterone homeostasis. Infection disrupts progesterone homeostasis in both animal models and humans. In pregnant *BALB/c* mice, infection reduces serum progesterone levels and induces adverse effects at the maternal–fetal interface, including inflammation, necrosis, and fetal resorption. Infected pregnant goats and ewes show decreased progesterone and oestrone sulphate levels following an initial increase in prostaglandin metabolites, leading to abortion or stillbirth. In human studies, anti-*T. gondii* IgG prevalence increases from 26.9% in the first/second trimesters to 32.7% in the third trimester. In seropositive women, progesterone increases significantly in the third trimester (*p* < 0.039), while estradiol increases in the second and third trimesters and prolactin increases in the second trimester (*p* < 0.021). Infected human villous explants show increased secretion of estradiol, progesterone, and HCG+β; treatment with azithromycin or pyrimethamine/sulfadiazine reduces both parasite load and hormone secretion, suggesting that hormonal dysregulation is a consequence of infection rather than a pre-existing condition. (**F**) Therapeutic implications and clinical considerations. The identification of *TgPGRMC*, *TgCYP450mt*, and *TgGST2* as mediators of progesterone’s antiparasitic effects suggests new therapeutic targets. Drugs that modulate progesterone receptor signaling or interfere with parasite steroid metabolism could be developed as novel anti-*T. gondii* agents. Protective adjunctive therapies include IOP, which significantly reduces abortion rates, inhibits the decrease in progesterone and estriol levels, and restores Th17/Treg balance through the *TLR4/NF-κB* pathway. S-methylcysteine combined with spiramycin mitigates hormonal disruptions (including progesterone) and promotes tissue recovery in infected rats. While progesterone is essential for pregnancy maintenance and is commonly supplemented in cases of luteal phase deficiency or threatened abortion, its immunosuppressive effects remain a concern. The finding that natural progesterone has direct antiparasitic effects is reassuring, but synthetic progestins may increase infection risk (pink boxes indicate progesterone and its effects; purple boxes denote *T. gondii* parasite structures and direct parasite–hormone interactions; red/orange boxes indicate synthetic progestins, calcium signaling, and infection risk; green boxes indicate protective/therapeutic effects and estradiol; blue boxes denote host immune modulation; arrows indicate increased or decreased expresson level or directional regulatory relationships (e.g., hormone → parasite effect, infection → hormone disruption). ↑ = increased (expression); ↓ = decreased (expression); → = proceed to.

Several key questions remain regarding the molecular mechanisms underlying progesterone–*T. gondii* interactions. The signaling pathways downstream of *TgPGRMC* require further elucidation, as does the functional significance of the parasite’s steroidogenic capacity. The relationship between progesterone-induced calcium fluxes [83,90] and *TgPGRMC* activation needs to be established. Most current evidence derives from animal models and in vitro systems. Prospective human studies examining the relationship between endogenous progesterone levels, progesterone supplementation, and the risk and severity of toxoplasmosis during pregnancy are needed. The findings of [92] provide a foundation for such studies. The development of drugs targeting *TgPGRMC* or other components of the parasite steroid signaling pathway represents a promising avenue for anti-*T. gondii* therapy. Additionally, the differential effects of natural progesterone versus synthetic progestins warrant further investigation to guide the safe use of these compounds in at-risk populations (Figure 4).

#### 2.3.3. Oxytocin

Toxoplasmosis in female rats causes an atypical increase in oxytocin and its receptor in the brain, specifically enhancing oxytocin mRNA in the paraventricular nucleus of the hypothalamus and oxytocin receptor mRNA in the posterodorsal medial amygdala [110]. This finding is significant because it reveals a sexually dimorphic mechanism underlying the parasite’s behavioral manipulation. While infected male rats lose their innate fear of cat odors through increased testosterone and vasopressin signaling, infected female rats exhibit the same loss of predator aversion without changes in gonadal steroids [110]. Instead, the authors propose that the enhanced oxytocin signaling from the hypothalamus to the medial amygdala may alter activity in social salience circuits, shifting the behavioral output from defensive avoidance toward approach-like responses to ambivalent cues such as cat odors [110]. Thus, *T. gondii* achieves sexually monomorphic behavioral changes through sexually dimorphic neural substrates.

#### 2.3.4. Follicle Stimulating Hormone (FSH)

The relationship between toxoplasmosis and Follicle Stimulating Hormone (FSH) levels is not consistently clear across different studies, and the existing evidence suggests that the parasite may have a limited or sex-specific impact on this hormone. A study conducted exclusively on male blood donors in Baghdad specifically examined the effect of toxoplasmosis on FSH levels. Their findings indicated that in both acute and chronic phases of toxoplasmosis, the mean concentration of FSH showed non-significant differences compared to controls. Specifically, the mean FSH levels were 6.41 ± 0.47 IU/mL and 6.515 ± 0.51 IU/mL for acute and chronic infections, respectively, leading the authors to conclude that toxoplasmosis did not produce a statistically significant alteration in FSH levels in their male cohort [111]. This observation of a lack of significant FSH change is partially supported by another human study, this time a pilot study conducted on immunocompetent human male volunteers. This investigation also found no statistically significant influence of latent Toxoplasmosis on serum levels of FSH in males. However, the authors of this pilot study caution that due to their limited sample size (60 subjects), they cannot definitively affirm the absence of an influence. They calculated that a much larger study (approximately 994 subjects) would be needed to reliably detect a potential effect in human males, leaving the question partially open [112].

In stark contrast to these human male studies, research on animal models—specifically on female mice—suggests a more complex role for FSH in the context of toxoplasmosis, particularly regarding ovarian dysfunction. A study on chronically infected female mice found that while the ovaries could respond to exogenous gonadotropins, there was evidence of pituitary FSH insufficiency. Specifically, following unilateral ovariectomy, infected female mice did not develop compensatory hypertrophy of the remaining ovary—a process normally driven by FSH. This pointed to a deficiency in endogenous FSH secretion from the pituitary. The researchers hypothesized that this impairment was likely due to cytokines released by the immune response to the parasite, which could inhibit the hypothalamic release of gonadotropin-releasing hormone (GnRH), thereby secondarily suppressing FSH (and LH) production in females [6,7]. Therefore, while human studies on males to date report no significant change in baseline FSH levels, animal research on females indicates that chronic toxoplasmosis can disrupt the functional capacity of the FSH axis, potentially leading to ovulatory problems. Whether *T. gondii* affects FSH in human females or in male animals remains unknown from these studies.

#### 2.3.5. Luteinizing Hormone (LH)

In female mice, chronic toxoplasmosis clearly suppresses LH function and disrupts the reproductive axis. Chronically infected female mice exhibited reproductive failure characterized by constant diestrous (a non-reproductive phase of the estrous cycle) along with ovarian and uterine atrophy. While basal serum LH levels were similar between infected and uninfected mice, infected females showed a significantly decreased pituitary responsiveness to a luteinizing hormone-releasing hormone (LHRH) analog. This indicates a dysfunction at the level of the hypothalamic-adenohypophyseal axis, specifically a reduction in the readily releasable pool of pituitary LH. A follow-up study by the same group further confirmed the absence of the critical preovulatory surge of endogenous LH from the pituitary in infected female mice, which directly prevented ovulation despite otherwise vigorous follicular growth stimulated by exogenous hormones [6,7].

In male mice, the evidence also points to a suppressive effect of *T. gondii* on LH, which correlates with reduced reproductive fitness. A study found that the level of LH in the urine of *T. gondii*-infected male mice was significantly lower compared to uninfected controls. This reduction in LH was directly correlated with diminished testicular function, including lower sperm counts, reduced numbers of spermatocytes and spermatids, and impaired spermatogenesis. These findings demonstrate a direct relationship between toxoplasmosis and decreased male reproductive fitness in mice, mediated in part by lowered LH levels. Later intervention studies using polysaccharides from medicinal fungi in *T. gondii*-infected male mice further support this pattern [113]. Both treatment with IOP or ginseng polysaccharide (GP), respectively, was able to increase serum LH levels (along with testosterone and FSH) while mitigating testicular damage and improving spermatogenic capacity. The fact that these therapeutic agents raised LH levels back toward normal strongly implies that chronic toxoplasmosis originally suppressed them.

In humans, the evidence regarding LH is more limited but suggests a potential for disruption, particularly in immunocompromised individuals. No large-scale human study specifically examining LH levels in immunocompetent males with latent toxoplasmosis was provided in these abstracts. However, a case report described a 37-year-old immunocompromised man (HIV-positive with a very low CD4 count) who presented with intracranial toxoplasmosis [114]. His laboratory evaluation revealed panhypopituitarism, including low levels of luteinizing hormone (LH) and testosterone, along with other pituitary hormones. A brain biopsy confirmed *T. gondii* as the cause of the brain lesions, which were believed to be directly causing his hormonal deficiencies [114]. This case demonstrates that in severe, disseminated toxoplasmosis affecting the central nervous system in an immunocompromised host, LH deficiency can occur as part of a broader pituitary failure. Whether latent toxoplasmosis affects LH levels in otherwise healthy humans remains unknown from these studies.

#### 2.3.6. Human Chorionic Gonadotropin (hCG)

A study on female mice chronically infected with *T. gondii* used hCG as a research tool to probe ovarian function [6,7]. In this experiment, the researchers administered hCG to infected female mice three days after they had received pregnant mare serum gonadotropin (PMSG). hCG is structurally similar to luteinizing hormone (LH) and is commonly used experimentally to mimic the LH surge that triggers ovulation. The key finding was thathCG successfully induced “superovulation” within 16 hin the chronically infected mice. This result is highly significant because it demonstrates that the ovaries of infected mice werenotthe site of failure—they remained capable of ovulating when given the appropriate hormonal signal (hCG). The problem, as the authors concluded, was not primary ovarian failure but rather the absence of theendogenouspreovulatory LH surge from the pituitary. In other words, toxoplasmosis impaired the brain and/or pituitary gland, but the ovaries themselves were still functionally responsive to exogenous gonadotropins like hCG.

#### 2.3.7. Prolactin

##### Prolactin as a Protective Factor Against Infection in Humans

Multiple human studies indicate that elevated prolactin levels are associated with a lower prevalence of toxoplasmosis, particularly in women. A cross-sectional study on 343 individuals found that women with high prolactin levels had a significantly lower prevalence of anti-*T. gondii* IgG antibodies compared to those with normal prolactin levels. Furthermore, as prolactin levels increased in women with hyperprolactinemia, the prevalence of toxoplasmosis decreased progressively [115]. This finding was corroborated by Dzitko et al., who studied a Polish population of 234 hyperprolactinemic individuals, 41 hypoprolactinemic individuals, and 281 controls. Women with hyperprolactinemia showed significantly lower seroprevalence (33.90%) than those with normal prolactin levels (45.58%). Notably, women with very high prolactin levels (>86 ng/mL) had a seroprevalence of only 12.50%, which was significantly lower than that of controls [116]. Both studies suggest that high prolactin may serve as a protective factor against *T. gondii* acquisition in women.

##### Prolactin in Pregnancy and Infertility

During pregnancy, hormonal changes create a complex relationship with toxoplasmosis. A study of 52 pregnant women across all three trimesters found that prolactin levels increased significantly in the second trimester among seropositive women (*p* < 0.021), alongside increases in 17β estradiol and progesterone [92]. The prevalence of anti-*T. gondii* IgG antibodies rose from 26.92% in the first and second trimesters to 32.7% in the third trimester. In the context of infertility, 520 infertile women over a ten-year period were examined and 65.77% were seropositive for anti-*T. gondii* IgG. However, serum prolactin levels were normal in 81.29% of these seropositive infertile women, suggesting that while latent toxoplasmosis is highly prevalent among infertile women, it does not typically present with abnormal prolactin levels [117]. Additionally, a case report described a 27-year-old woman with secondary amenorrhea and moderately elevated prolactin levels who was found to have both a nonfunctioning pituitary adenoma and *T. gondii* bradyzoites within the adenoma [118]. This rare presentation highlights that sellar toxoplasmosis can coexist with pituitary lesions and hyperprolactinemia.

##### Prolactin Receptors and Direct Parasite Binding

The interaction between prolactin and *T. gondii* appears to involve direct binding at the molecular level. Sheep prolactin binds specifically to live tachyzoites of both type I (RH) and type II (*ME49*) *T. gondii* strains, as demonstrated using fluorescein- and biotin-labeled prolactin. This binding was confirmed to be specific through competitive inhibition analysis [119]. Furthermore, *T. gondii* infection itself lowers the expression of the prolactin receptor (*PRLR*) on THP-1 human monocytic cells [82]. Treatment with exogenous prolactin further decreased the expression of estrogen receptors (*ERα* and *ERβ*), indicating complex cross-talk between hormonal signaling pathways during infection. 17β-estradiol modulates the expression of *PRLR* in *T. gondii*-infected macrophages and monocytes in an *AKT* and *ERK*-dependent manner, increasing *PRLR* expression in infected macrophages while decreasing it in infected monocytes [14].

##### Prolactin’s Anti-Parasitic Effects in Cell Cultures

In vitro studies consistently demonstrate that prolactin inhibits *T. gondii* proliferation. Recombinant human prolactin (rhPRL) on three cell lines (murine L929 and human Hs27 and HeLa) was evaluated and pre-incubation of tachyzoites with rhPRL significantly reduced their replication ability by up to 36.15%, primarily by limiting the parasites’ capacity to penetrate host cells. No toxic effects of rhPRL on host cells or extracellular tachyzoites were observed [120]. Expanding on this, peripheral blood mononuclear cells (PBMCs) from female hyperprolactinemia patients found that both exogenous rhPRL and autologous serum prolactin significantly restricted intracellular growth of *T. gondii*. This effect was positively correlated with increased production of IL-10, suggesting an immunomodulatory mechanism [121]. Similarly, prolactin, along with TNF-α, induced *T. gondii* static activity in murine microglia, leading to intracellular killing of *T. gondii* and release of IL-1β, IL-3, and IL-6, with the effect partially dependent on ICAM-1 expression [122].

##### Prolactin’s Protective Effects in Animal Models

In vivo animal studies confirm that prolactin administration confers protection against toxoplasmosis. Mice treated with recombinant mouse prolactin for five days prior to toxoplasmosis showed a significant increase in survival rate, a significant decrease in the number, size, and DNA content of brain cysts, and noticeable improvement in histopathological lesions in brain and liver tissues. These protective effects were associated with significant rises in serum levels of anti-*T. gondii* IgM, IFN-γ, and TNF-α, indicating stimulation of both humoral and cell-mediated immunity [123]. Mice lethally infected with *T. gondii* and treated with prolactin (0.5–2 mg/kg twice daily for 12 days) were protected against death in a dose-dependent manner (*p* < 0.001). Survival rates reached 50–70% when prolactin was combined with either rIFN-γ or rTNF-α, and a slight synergistic effect on reducing brain cyst formation was observed with prolactin plus TNF-α [124]. The authors concluded that prolactin regulates endogenous TNF-α production and plays an important role in modulating host immune defense against toxoplasmosis. One methodological study described the use of xanthine oxidase as a label in solid-phase immunoassays, including the detection of anti-*T. gondii* IgG antibodies and prolactin measurement [125]. This technical paper demonstrates that prolactin and *T. gondii* serology can be measured using the same labeling technology, but it does not address any biological relationship between the two.

#### 2.3.8. Gonadotropin-Releasing Hormone (GnRH)

Two studies point to a model in which toxoplasmosis leads to suppression of GnRH, which in turn causes downstream impairment of the pituitary-gonadal axis. However, the evidence comes from different contexts: one in female mice with chronic infection, and the other in male humans with acute infection. In female mice with chronic toxoplasmosis, the etiology of ovarian dysfunction was investigated. It was found that while the ovaries of infected mice remained capable of responding to exogenous gonadotropins (PMSG and hCG) by undergoing folliculogenesis and even superovulation, the mice failed to ovulate on their own due to the absence of the endogenous preovulatory LH surge. Furthermore, unilateral ovariectomy did not result in compensatory hypertrophy of the remaining ovary, indicating insufficient FSH. Taken together, these findings pointed to a central defect at the level of the hypothalamus or pituitary, rather than primary ovarian failure. The authors directly hypothesized that “cytokines released peripherally in response to the parasite reached the hypothalamus and initiated a sequence of events that inhibited the pulsatile release of gonadotropin-releasing hormone (GnRH), leading to the subsequent impairment of the pituitary-ovarian axis.” This study thus places GnRH suppression at the center of *T. gondii*-induced reproductive dysfunction in female mice [6,7].

In male humans with acute toxoplasmosis, an outbreak of toxoplasmosis in a male boarding school in Turkey was studied. Among 40 infected male patients aged 17–18 years, nine (group B) were found to have low levels of FSH, LH, free testosterone, and total testosterone, consistent with hypogonadotropic hypogonadism (i.e., gonadal failure caused by insufficient pituitary stimulation rather than a primary testicular problem). The remaining 31 patients (group A) had normal sex hormone levels. The key finding was that interleukin-1 beta (IL-1β) levels were significantly higher in group B patients than in group A, and IL-1β levels correlated negatively with FSH, LH, and testosterone across all infected patients. While this study did not measure GnRH directly, the pattern of low FSH and LH with normal pituitary responsiveness (implied) is classic for hypothalamic GnRH deficiency. The authors concluded that acute toxoplasmosis causes temporary hypogonadotropic hypogonadism, and the suppressive effect is likely mediated by IL-1β acting on GnRH secretion [126], Table 2.

#### 2.3.9. Anti-Müllerian Hormone

The relationship between toxoplasmosis and Anti-Müllerian Hormone (AMH) levels in young, unmarried women aged 20–25 years was investigated. AMH is a hormone produced by ovarian granulosa cells and is a clinically important marker of ovarian reserve—the number of remaining eggs in a woman’s ovaries. Higher than normal AMH can be associated with polycystic ovary syndrome (PCOS), while low AMH indicates diminished ovarian reserve. The key finding from both studies is that toxoplasmosis is associated with elevated AMH levels, and this elevation correlates positively with various pro-inflammatory and regulatory cytokines [127,128]. In the first study, blood samples were collected from 80 unmarried women (40 infected with *T. gondii* and 40 uninfected controls). They measured levels of IL-2, IL-10, IL-12, and AMH. The results showed a significant increase in IL-2, IL-10, and IL-12 levels in infected women compared to the control group. Importantly, there was a positive correlation between all three cytokines and AMH levels in infected women. The authors concluded that this positive correlation supports the possibility of adopting the AMH level as an indicator for future cases of polycystic ovariancysts and infertility in women with toxoplasmosis [127]. In other words, toxoplasmosis, through its effects on the immune system, may raise AMH levels, which could predispose women to PCOS and related fertility problems. The second study appears to be a follow-up investigation by the same group using the same study design (80 women total: 40 infected, 40 controls) but focusing on different inflammatory cytokines: IL-1β, IL-6, and tumor necrosis factor alpha (TNF-α). The results mirrored the first study: infected women had significantly higher levels of IL-1β, IL-6, and TNF-α compared to controls. Once again, AMH concentrations correlated significantly and positively with all three immune parameters [128]. The authors concluded that the strong association between AMH and these inflammatory cytokines implies that toxoplasmosis may influence ovarian reserve markers through immune-mediated mechanisms.

## 3. Male Reproductive Tract: Testicular Tropism and Seminal Shedding

The possibility of venereal transmission—infection through semen during natural mating or artificial insemination—is inconsistent across species and experimental models, with some studies demonstrating clear transmission while others find no evidence. The testis represents an immunologically privileged site essential for germ cell development, yet *T. gondii* efficiently breaches the blood–testis barrier to establish productive infection. Seminal studies using in vivo bioluminescence imaging demonstrated rapid parasite dissemination to testes following systemic inoculation, with intense photon signals detectable by day 4–5 post-infection and strong correlation between testicular signal and viable parasite counts [12]. This establishes the testis as a significant site of acute parasite replication. Notably, MyD88-deficient mice fail to contain infection and exhibit parasite loads up to two orders of magnitude greater than wild-type, establishing the critical role of Toll/interleukin-1 receptor signaling in testicular defense [12]. Direct visualization studies confirm testicular tropism across species. In experimentally infected mice and rats, parasites localize to the testes and epididymis with accompanying inflammatory infiltration and architectural disruption [12,129]. Histological demonstrations in animals and humans have confirmed the presence of *T. gondii* trophozoites and proliferative forms, with special consideration given to the sex organs as sites of infection [130].

### 3.1. Species-Specific Patterns of Seminal Shedding

#### 3.1.1. Sheep: Brief Shedding but Demonstrated Transmission

The most rigorous early investigation of seminal shedding in ruminants came from Teale, Blewett, and Miller (1982) [131]. Six young rams were inoculated subcutaneously with 2000 *T. gondii* tissue cysts, with observations continuing for over 90 days post-infection (pi). Three of six infected rams produced infective semen, each on two occasions, occurring between 14 and 26 days pi—demonstrating that “the production of infective semen was restricted to a brief period shortly after infection” [131]. The authors concluded that venereal transmission was unlikely to be significant in the spread of ovine toxoplasmosis, based on the brief duration of shedding, absence of diagnostic clinical changes, and the likelihood that acutely ill rams would be removed from breeding.

However, subsequent studies demonstrated that transmission can occur despite this narrow window. Sexual transmission of *T. gondii* in sheep has been definitively demonstrated. Male sheep were inoculated either orally with 2.0 × 10^5^ oocysts (P strain) or subcutaneously with 1.0 × 10^6^ tachyzoites (*RH* strain). After inoculation, seronegative ewes were exposed to natural mating. *Toxoplasma gondii* was isolated from the semen of infected rams before mating. Following natural mating, 5 of 12 females seroconverted, with one ewe developing a macerated fetus on day 70 post-coverage. The parasite was isolated from tissue pools of all five seroconverted females and their lambs, demonstrating both sexual and subsequent vertical transmission [132]. Artificial insemination with semen experimentally contaminated with tachyzoites was shown to infect ewes in a dose-dependent manner: 33.3% seroconversion with 6.5 × 10^4^ tachyzoites versus 100% with 4 × 10^7^ tachyzoites. Nested PCR confirmed parasitemia in 93.3% of infected animals across both dose groups [133]. These findings were extended by demonstrating that frozen-thawed semen spiked with tachyzoites remained infectious. All five ewes inseminated with contaminated frozen semen seroconverted by day 7 post-insemination, with parasites detected in tissues by immunohistochemistry—significant implications for artificial insemination programs, as standard freezing protocols do not inactivate the parasite [134].

Not all studies support significant venereal transmission in sheep. Semen from 77 apparently normal rams was tested by mouse passage and found no patent infections. Two rams with chronic toxoplasmosis were repeatedly mated with seronegative ewes; none seroconverted, and all lambed satisfactorily [135]. Similarly, semen from five seropositive rams were tested negative for *T. gondii* by both endpoint and real-time PCR [136]. These negative findings likely reflect that shedding is restricted to acute infection [131], and chronically infected rams do not continuously shed. In rams with experimental oral infection, *T. gondii* DNA was detected in 12.5% of sperm samples at 15 weeks post-infection, with infected animals showing characteristic fever, seroconversion, and deteriorating sperm quality despite intermittent parasite detection [137]. Notably, sulphadimidine administered 24 h post-infection or at two months post-infection failed to prevent deterioration in sperm quality parameters, possibly reflecting the inability of sulphadimidine to adequately penetrate the blood–testis barrier [137]. Early in vitro work further supported the parasite’s tropism for male reproductive tissues by successfully cultivating *T. gondii* in lamb testicular cell culture [137,138].

#### 3.1.2. Goats: Prolonged Shedding but Limited Follow-Up

The Dubey and Sharma (1980) [139] goat study remains the most frequently cited evidence for prolonged shedding. Three one-year-old male goats were orally inoculated with 10^4^ oocysts of the *GT-1* strain. All three shed *T. gondii* in semen, with excretion periods of 52, 16, and 5 days, respectively. Mouse bioassay confirmed infectivity [139]. The 52-day duration substantially exceeds the 12-day window (14–26 days pi) reported in rams. The animal shedding for 52 days was euthanized while still shedding, leaving the true maximum duration unknown. Possible explanations for species differences include inoculation route (oral vs. subcutaneous) or strain variation [139]. Beyond this study, data on seminal shedding in goats remain sparse, and the finding has not been systematically replicated.

#### 3.1.3. Mice: Sexual Transmission with Reduced Fertility

Sexual transmission in mice using the *RH* strain was directly evaluated. Male *Balb/C* mice were intraperitoneally inoculated with tachyzoites; semen collected from epididymides 48 h later was examined by Giemsa staining and PCR. Female mice mated with infected males showed dramatically reduced pregnancy rates: only 4 of 20 (20%) became pregnant compared to 17 of 20 (85%) in controls (*p* = 0.0001). Despite detection of parasite DNA and whole organisms in semen, no abortion or death was observed in females, and no transmission to females or offspring was detected via mating. The authors suggested that infected male mice “cannot entirely mate with females due to reduction in male weapon and body size, physiological vigor and energy” [140]. This study demonstrates that while toxoplasmosis impairs male reproductive capacity and fertility, successful sexual transmission may be limited by behavioral and physiological changes in infected males.

#### 3.1.4. Rats: Impaired Reproductive Parameters

Male Wistar rats infected orally with 5 × 10^3^ tissue cysts. Over 60 days post-inoculation, they observed significantly decreased sperm motility (days 10–60), decreased sperm concentration (days 10, 30, 40, 60), and increased sperm abnormalities (days 30 and 40). Epididymal weight was significantly decreased on day 30 pi. No pathological lesions were detected in the pituitary gland or testes [129]. These findings were extended to latent infection. Infected rats exhibited a significant progressive loss of body weight and absolute testicular weight. Sperm motility, viability, and concentration showed highly significant decreases throughout the observation period, with highly significant increases in abnormal sperm forms. Histopathological insults in the testes were documented. These studies confirm that both acute and latent toxoplasmosis adversely affect male rat reproductive parameters, though neither directly demonstrated seminal shedding duration [141].

#### 3.1.5. Rabbits: Limited Window of Infectivity

Early experimental infection of male rabbits demonstrated that infective *T. gondii* could be isolated from the testicles and accessory gonads only up to the 29th day post-infection, and attempts to transmit infection through sexual intercourse to *T. gondii*-free female rabbits were uniformly unsuccessful, suggesting that sexual transmission, even if biologically possible, may be epidemiologically insignificant [142].

#### 3.1.6. Dogs: Molecular Detection in Breeding Animals

*Toxoplasma gondii* in semen of breeding dogs from southeastern Iran was investigated using a highly sensitive REP-based nested PCR assay. Among 36 purebred male dogs (31% seropositive), 8.33% (3/36) had detectable *T. gondii* DNA in semen. One positive sample exhibited orchitis with significant sperm abnormalities including reduced motility and tail defects. However, the authors noted “no relation between *T. gondii* detection in semen and reproductive disorders” in this population, calling for further studies to confirm sexual transmission [143].

#### 3.1.7. Cats: A Notable Negative Finding

Given that cats are the definitive hosts for *T. gondii*, their potential role in venereal transmission merits investigation. Twelve male cats were experimentally infected with either 600 tissue cysts (*P* strain, type III) or 2 × 10^5^ tachyzoites (*RH* strain, type I). Despite confirmed seroconversion and parasitemia, no *T. gondii* was detected in semen samples collected on days 7, 14, 21, 28, 42, 56, and 70 pi. Testicular and epididymal tissues collected at orchiectomy on day 70 were also negative by nested PCR, mouse bioassay, histopathology, and immunohistochemistry [144]. The authors concluded: “sexual transmission in domestic cats does not appear to be a major route of toxoplasmosis, possibly demonstrating the tendency of this protozoan to develop a response directed to the formation and excretion of oocysts in the feces of these definite hosts” [144]. This finding is biologically plausible: the parasite’s evolutionary strategy in the definitive host prioritizes fecal–oral transmission, and reproductive tract colonization may be selected against.

#### 3.1.8. Field Studies: Prevalence in Naturally Infected Animals

A field study of 92 rams from four regions in Tunisia was conducted. Using PCR on semen samples, they found a molecular prevalence of 51.09% (±10.21%) for *T. gondii* DNA, with an overall seroprevalence of 39.13% (±9.97%). Risk factors included geographic location and number of completed mating seasons (*p* < 0.05). Concordance between sero- and molecular prevalence was fair (Kappa = 0.33). Sequencing of five positive samples revealed 100% identical haplotypes, matching sequences previously reported in chickens, cats, European polecats, and humans from multiple countries, indicating wide geographic distribution and lack of host specificity [145]. This high molecular prevalence in field-collected semen samples—contrasting with the negative findings of Blewett et al. in 77 apparently normal rams—warrants explanation [135]. Possible factors include: (1) different detection methods (PCR detects DNA from non-viable as well as viable organisms); (2) different geographic regions with varying infection pressures; (3) different stages of infection in sampled animals; or (4) different parasite strains. It is noted that “further studies concerning its venereal transmission capacity are needed prior to recommending a systematic screening of *T. gondii* DNA in rams’ semen” [145].

#### 3.1.9. Human Studies: HIV/AIDS, Latent Infection, and Reproductive Consequences

Autopsy studies of AIDS patients identified testicular *T. gondii* in 3.7–39% of cases, sometimes as the only extracerebral manifestation [146,147,148,149]. A comprehensive study of 56 AIDS patients with systemic opportunistic infections found light-microscopic evidence of *T. gondii* in the testes of 39% of cases, leading to speculation about possible sexual transmission of the parasite based on the prevalence and histologic features of testicular involvement [146]. Symptomatic orchitis has been documented as the initial presentation of disseminated toxoplasmosis, with nephrotic syndrome remitting only after orchiectomy and anti-parasitic therapy [150]. Case reports have further documented concurrent cerebral toxoplasmosis and myocardial involvement in AIDS patients with testicular disease, emphasizing the disseminated nature of infection in immunocompromised hosts [151]. Histopathological examination reveals both pseudocysts (containing bradyzoites) within necroses and inflammatory foci by conventional staining, while trophozoites became apparent only with immunohistochemical methods [147], highlighting the importance of sensitive detection techniques. A broader histopathological comparison of 57 AIDS patients with age-matched controls further revealed that infected individuals exhibited significantly impaired spermatogenesis, along with prominent thickening of the basement membrane and interstitial fibrosis [149]. HIV/AIDS exacerbates *T. gondii*-induced reproductive dysfunction through immunosuppression-driven reactivation and direct endocrine disruption. In HIV-infected pregnant women, *T. gondii* seroprevalence reaches 41%, with co-infection associated with increased miscarriage, stillbirth, and congenital defects [152,153]. In non-pregnant individuals, cerebral toxoplasmosis—occurring when CD4+ counts fall below 100 cells/μL—can cause isolated gonadotrophin deficiency. A documented case report describes an HIV-positive woman who developed secondary amenorrhea and ovarian failure due to a pituitary toxoplasmosis lesion that impaired LH/FSH secretion [154]. Additionally, toxoplasmosis of the hypothalamus or pituitary in AIDS patients can precipitate hypogonadotropic hypogonadism, directly compromising fertility [155]. Thus, in HIV-endemic regions, *T. gondii* co-infection represents a lesion-mediated cause of central reproductive failure, Table 3 and Table 4.

### 3.2. Mechanisms of Male Reproductive Immunopathology

The testis maintains immune privilege to protect germ cells expressing novel antigens from autoimmune attack. Toxoplasmosis triggers robust pro-inflammatory responses that compromise this privilege, fundamentally altering the testicular microenvironment.

#### 3.2.1. Innate Immune Recognition and Signaling

Toxoplasmosis initiates powerful innate immune responses through Toll/interleukin-1 receptor (TIR) signaling via the adaptor protein MyD88. MyD88-deficient mice fail to contain infection, exhibiting parasite loads two orders of magnitude higher than wild-type, with bioluminescence imaging confirming uncontrolled testicular dissemination [12]. Notably, ICE-deficient mice (lacking functional IL-1 and IL-18) control infection normally, suggesting MyD88 signaling occurs through Toll-like receptors rather than IL-1R or IL-18R [12]. However, the host defense is complex and redundant, as single deficiencies in *TLR1*, *TLR2*, *TLR4*, *TLR6*, or *TLR9* do not independently increase susceptibility to infection [12]. Male germ cells express *TLR11*, which recognizes *T. gondii*-derived profilin and initiates innate immune responses characterized by inflammatory cytokine production [160]. The *TLR4-P2X7R/NLRP3* inflammasome pathway is activated in the testes of infected mice, contributing to the inflammatory cascade [161]. Neutrophil IL-12 production requires *JNK2* MAP kinase, demonstrating pathway-specific host defense requirements [162]. The critical role of immune signaling in controlling testicular infection is further illustrated by observations in immunocompromised hosts, where testicular toxoplasmosis can become clinically apparent [146,148].

#### 3.2.2. Cytokine Profiles and T Helper Responses

Infection shifts the testicular cytokine milieu toward a Th1-polarized state with elevated IFN-γ, TNF-α, and IL-6 [163,164]. Acute infection induces a strong Th1 response with increased pro-apoptotic *Bax* in spermatogenic cells, correlating with sustained testicular damage [163]. This inflammatory environment, while necessary for parasite control, creates bystander damage to developing germ cells. Studies on gender and sex hormones in susceptibility demonstrate that female animals died significantly earlier than males after peroral infection, with early mortality of females associated with greater numbers of tachyzoites and severe necrosis in their small intestines [165]. Treatment of female mice with testosterone resulted in markedly reduced intestinal parasite numbers and pathology, indicating that gender and sex hormones are important factors for determining susceptibility to toxoplasmosis [165]. Both female and male reproductive tissues experience cytokine-driven pathology, though with distinct consequences reflecting tissue-specific functions.

#### 3.2.3. Male: Germ Cell Apoptosis and Spermatogenic Failure

*T. gondii*-induced testicular damage occurs through multiple convergent pathways. Direct parasite-trophozoite interaction with spermatozoa in vitro causes headless sperm, twisted tails, and plasma membrane disruptions through mitochondrial membrane potential loss without ROS modulation—pointing to mitochondrial dysfunction as the primary driver [8]. *Toxoplasma gondii* demonstrates definitive tropism for reproductive tissues, colonizing the testes and epididymis, altering tissue homeostasis, and directly damaging human spermatozoa through mitochondrial dysfunction [8]. In vivo, infection reduces seminiferous tubule diameter, depletes germ cells, and disrupts spermatogenic epithelium architecture [12,141,166,167]. In mice, infection reduces the number of spermatocytes and spermatids [113], disrupting the normal architecture of the seminiferous epithelium. Sperm concentration and motility decline significantly while morphological abnormalities increase, with peak abnormality rates of 30% at 30–40 days post-infection [129]. A comprehensive time-course study in male Wistar rats found that sperm motility was significantly decreased in infected animals at all time points measured from day 10 to day 60 post-inoculation, with sperm concentration significantly reduced on days 10, 30, 40, and 60, and a marked increase in sperm abnormalities including bent tail, loss of hook shape, and detached heads [129]. DNA damage, evidenced by increased acridine orange, aniline blue, and toluidine blue staining, indicates chromatin abnormalities persisting beyond acute infection [168]. To investigate the influence of acute toxoplasmosis on the reproductive function of male mice, a study found that infection led to significantly reduced testicular LDH-X levels, lower sperm concentration and motility, and a higher percentage of deformed spermatozoa compared to controls, confirming a direct negative impact on male reproductive function [169].

**Figure 5:**
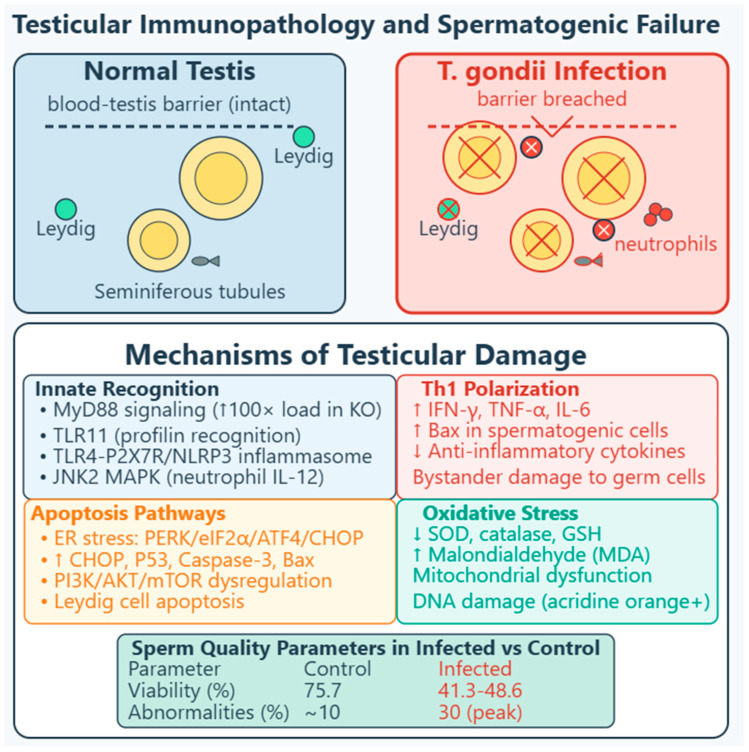
*Toxoplasma gondii* breaches the blood–testis barrier, triggering inflammatory responses, Leydig cell dysfunction, and germ cell apoptosis through multiple convergent pathways including ER stress, oxidative stress, and cytokine-mediated damage. The bottom table shows sperm quality parameters in infected versus control animals: viability decreased from 75.7% (control) to 41.3–48.6% (infected), and morphological abnormalities increased from approximately 10% (control) to 30% peak (infected) [129,137], (Yellow circles with dark yellow centers represent seminiferous tubules (red crosses indicate damage), green circles represent Leydig cells (red crosses indicate apoptosis), red circles with white crosses represent *T. gondii* tachyzoites, small red circles represent neutrophil infiltrate, dashed blue lines indicate intact blood–testis barrier, dashed red lines with V-shaped symbols indicate barrier breach, normal fish-shaped icon represents normal sperm, and deformed fish-shaped icon represents damaged sperm. ↑ = increased (expression); ↓ = decreased (expression)).

The ram study provides a detailed quantitative assessment of sperm quality parameters following infection during the prepubertal period. Sperm viability was significantly reduced in all infected groups compared to controls (41.33–48.55% vs. 75.71% in controls). The hypo-osmotic swelling test revealed similarly dramatic differences, and total morphological abnormalities were significantly higher in infected groups, with head defects and tail abnormalities particularly elevated [137]. Collectively, these findings demonstrate that toxoplasmosis compromises male fertility through a dual mechanism: it disrupts the physiological process of spermatogenesis within the testes and directly impairs the functional integrity of mature spermatozoa, with effects that persist for months after initial infection [129]. Apoptosis occurs via endoplasmic reticulum stress through the *PERK*/*eIF2α*/*ATF4*/*CHOP* pathway, with upregulation of *CHOP*, *P53*, *Caspase-3*, and *Bax* [161,170]. Mechanistic investigations reveal that *T. gondii* induces Leydig cell apoptosis via the endoplasmic reticulum stress pathway, as evidenced by upregulation of *CHOP*, *P53*, *Caspase-3*, and *Bax*, with concurrent increases in *Bcl-2* expression [170]. Concurrently, the *PI3K*/*AKT*/mTOR survival pathway is often dysregulated in infected testicular tissue [161,171]. Chronic infection also alters the expression of genes crucial for maintaining the blood–testis barrier [172], potentially compromising the immune-privileged environment that protects developing germ cells from systemic inflammatory responses. Transcriptomic analysis of testicular tissue from chronically infected mice identified 250 differentially expressed genes, including those involved in blood–testis barrier integrity (e.g., *PTGDS*), offering new insights into how infection disrupts the spermatogenic microenvironment [172]. Oxidative stress represents a unifying mechanism across both sexes. Testicular infection depletes superoxide dismutase, catalase, and glutathione while increasing malondialdehyde, creating biochemical hostility to spermatogenesis [173,174,175]. In *T. gondii*-infected rats, significant decreases in superoxide dismutase activity on day 80, catalase activity on days 60 and 80, and reduced glutathione and total antioxidant capacity on day 80 were observed in testis tissue, while malondialdehyde concentration increased on day 70 post-infection, confirming that infection causes oxidative stress in testicular tissue which may adversely affect the male reproductive system [173,174], Table 5, Figure 5.

**Figure 6:**
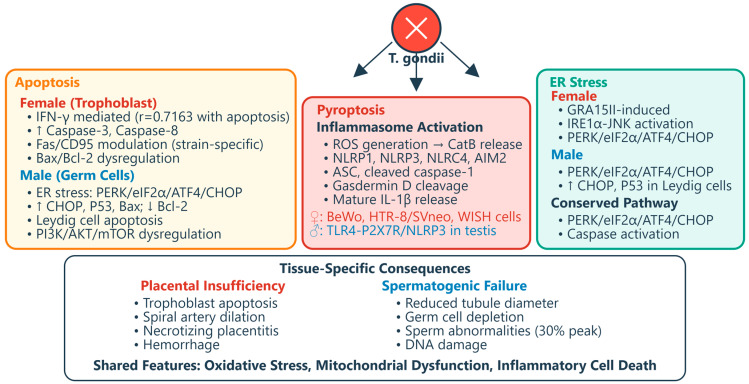
Convergent cell death mechanisms in female and male reproductive tissues. Both sexes experience apoptosis, pyroptosis, and ER stress-mediated cell death through shared molecular pathways, though with tissue-specific consequences (Black arrows originating from *T. gondii* (red circle with white cross, center) indicate direct induction of each cell death pathway. Colored boxes distinguish the three pathways: yellow = Apoptosis, red = Pyroptosis, teal = ER stress. Within each pathway box, red text denotes findings in female reproductive tissues (e.g., trophoblast, placenta), while blue text denotes findings in male reproductive tissues (e.g., germ cells, Leydig cells, testis). The bottom panel summarizes tissue-specific downstream consequences: placental insufficiency (female, left) and spermatogenic failure (male, right), with shared unifying mechanisms listed beneath). ↑ = increased (expression); ↓ = decreased (expression); → = proceed to.

These effects persist even without detectable local parasites, suggesting self-perpetuating tissue injury cycles [129]. Acute toxoplasmosis in mice results in a significant increase in oxygen-free radicals and a subsequent compensatory rise in superoxide dismutase (SOD) in both serum and testicular tissue, indicating the induction of oxidative stress [173]. In vitro exposure of bovine sperm to soluble *T. gondii* antigens impairs sperm motility, mitochondrial activity, and membrane integrity, and while it does not affect fertilization rates, it negatively impacts subsequent early embryonic development [10]. Toxoplasmosis, a parasitic infection, disrupts sexual functions, resulting in a decline in reproductive and economic performance in sheep. A high prevalence of *T. gondii* DNA (51%) was found in the semen of rams from Tunisian farms, with risk factors including location and breeding history, underscoring the potential for venereal transmission and the need for screening in breeding programs [145]. In breeding dogs with reproductive disorders, *T. gondii* DNA was detected in the semen of 8.33% of animals, with one positive case exhibiting orchitis and sperm abnormalities, suggesting a potential link that warrants further investigation [143]. Oral inoculation of mice with *T. gondii* oocysts results in dose-dependent mortality and widespread histopathological changes, including in the testes, demonstrating the multi-organ damage caused by disseminated infection [175], Table 6, Figure 6.

### 3.3. Male Hormonal Disruption and Endocrine Crosstalk

#### 3.3.1. Testosterone Dysregulation and HPG Axis Effects

Testosterone regulation in infected males shows complex, apparently contradictory patterns. Meta-analysis of 18 human studies confirms elevated testosterone in latently infected males (pooled increase of 0.73 units), proposed as parasite manipulation enhancing sexual behavior and transmission [1,21,22,23]. Infected men show increased perceived dominance and masculinity, consistent with androgenic effects [183]. Conversely, infected women tend to exhibit lower testosterone concentrations than uninfected controls, a sex-specific divergence that may underlie the distinct behavioral shifts observed in toxoplasmosis [21,22,23,184]. However, the literature is not unanimous. A study in Palestinian butchers found median testosterone levels of 351 ng/dL in IgG-positive versus 428.5 ng/dL in seronegative individuals, with raw meat consumption and soil exposure as significant risk factors [185]. Similarly, a study in Iranian men with chronic toxoplasmosis reported significantly lower serum testosterone compared to controls [186]. Animal studies similarly show variable outcomes, with most reporting testosterone declines in infected rodents and hyenas, though some rat studies note increases, suggesting species-specific or strain-specific effects [1,187]. In spotted hyenas, a negative association between toxoplasmosis and plasma testosterone was detected in female cubs, subadults, and adult males, further supporting species-specific and age-dependent endocrine disruption [188].

Despite acute in vitro findings of testosterone elevation, chronic infection models consistently demonstrate significant reductions in both serum and intratesticular testosterone levels [166,167,187]. This hormonal suppression is mechanistically linked to downregulation of key steroidogenic enzymes, including steroidogenic acute regulatory protein (*StAR*), cytochrome *P450* side-chain cleavage enzyme (*P450scc*), and 17β-hydroxysteroid dehydrogenase (*17β-HSD*) [161,171]. The parasite actively weakens host hormone signaling through targeted downregulation of steroidogenic enzymes including *StAR*, *P450scc*, and *17β-HSD* in Leydig cells and hormone receptors including *ERα*, *GPER*, and *PRLR* in immune cells. Direct infection of Leydig cells by *T. gondii* has been demonstrated in vitro, leading to increased testosterone and inflammatory mediators (MCP-1, IFN-γ) in the short term, but apoptosis of Leydig cells via the endoplasmic reticulum stress pathway (*CHOP*, *Caspase-3*, *Bax* upregulation) over longer periods [164,170]. The discrepancy between acute in vitro testosterone elevation and chronic in vivo suppression may reflect initial Leydig cell hyperstimulation followed by exhaustion and apoptosis—a pattern consistent with the dualistic outcomes reported in human studies. The hypothalamic–pituitary–gonadal (HPG) axis is affected at multiple levels. Chronically infected rodents exhibit lower luteinizing hormone (LH) levels and elevated follicle-stimulating hormone (FSH), suggesting both primary testicular dysfunction and altered central feedback regulation [113,166]. Specifically, one study reported significantly higher mean serum FSH in infected rats compared to controls at 40, 50, and 60 days post-infection, while intratesticular testosterone became significantly lower only by day 60 [166]. These findings align with observations from a human toxoplasmosis outbreak, where acutely infected males developed temporary hypogonadotropic hypogonadism characterized by suppressed LH and FSH that correlated negatively with elevated IL-1β levels [126]. In that outbreak, nine of 40 acutely infected males showed low sex hormones, with IL-1β levels significantly higher in these individuals and negatively correlated with FSH, LH, free testosterone, and total testosterone [126]. Cytokine-mediated inhibition of hypothalamic gonadotropin-releasing hormone (GnRH) pulsatility—a mechanism documented in female murine toxoplasmosis [6,7]—likely contributes to this central suppression in males as well.

Beyond direct endocrine disruption, *T. gondii*-induced testosterone alterations have broader biological implications. Infection enhances sexual attractiveness and attenuates innate fear of cat odor in male rats, effects associated with upregulation of testicular steroidogenesis genes and increased testosterone production—a proposed parasite manipulation strategy to facilitate transmission to the feline definitive host [189]. In humans, latent toxoplasmosis correlates with increased perceived dominance and masculinity in infected men, consistent with elevated testosterone [183], and has been associated with altered offspring sex ratios, potentially reflecting hormone-driven parental effects [190]. Furthermore, a study on male infertility found that among *T. gondii*-seropositive infertile men, a substantial proportion (38.2% of IgG-positive and 75% of IgM-positive cases) had disturbed testosterone-to-estradiol ratios, with the T:E2 ratio being significantly lower in IgM-positive cases (8.68 ± 1.95) compared to seronegative controls (10.45 ± 0.54), highlighting the clinical relevance of toxoplasmosis in male reproductive health [191]. The *RhD* blood group phenotype may modulate susceptibility to *T. gondii*-induced behavioral changes in a testosterone-dependent manner, with *RhD*-positive individuals showing relative protection against infection-associated reaction time prolongation [21,22,23]. This protective effect of *RhD* positivity against *T. gondii*-induced reaction time impairment has been confirmed in both men and women, and testosterone concentration strongly influences reaction times, suggesting that *RhD* polymorphism may be maintained by balancing selection related to geographic variation in toxoplasmosis prevalence [21,22,23].

Decision-making under risk involves balancing the potential of gaining rewards with the possibility of loss and/or punishment. Tolerance to risk varies between individuals. Understanding the biological basis of risk tolerance is pertinent because excessive tolerance contributes to adverse health and safety outcomes. Using a rodent model of the balloon analogous risk task, studies show that latent toxoplasmosis leads to a greater tolerance of reward forfeiture. Furthermore, effects of the infection on risk can be recapitulated with testosterone supplementation alone, demonstrating that greater testosterone synthesis by the host post-infection is sufficient to change risk tolerance [192]. However, a study in female humans found no significant association between toxoplasmosis and financial risk-taking behavior measured by experimental economic tasks, suggesting that the effect of toxoplasmosis on risk tolerance may be sex-dependent [193].

The effect of *T. gondii* on rat behavior assessed in a colony of free-ranging wild/laboratory hybrid rats allowed to compete freely for food and mates in an outdoor naturalistic enclosure reveals that toxoplasmosis has no effect on social status or mating success, both the product of costly and competitive activities. However, the propensity to explore novel stimuli in their environment is higher in infected than in uninfected individuals. These results are consistent with the hypothesis that *T. gondii* only affects the behavioral traits thatselectively benefit the parasite, rather than causing a general alteration of rat behavior [194].

The protozoan *T. gondii* manipulates the behavior of its rodent intermediate host to facilitate its passage to its feline definitive host. This is accomplished by a reduction in the aversive response that rodents show towards cat odors, which likely increases the predation risk. Females, on average, show similar changes as males. However, behaviors that relate to aversion and attraction are usually strongly influenced by the estrus cycle. Studies replicating behavioral effects of *T. gondii* in female rats and characterizing the role of the estrus cycle demonstrate that infected females lose preferences for safety and spend more time investigating social novelty. Infection makes females less risk-averse and more exploratory, and this effect is influenced by the estrus cycle. Uninfected rats prefer rabbit urine to bobcat urine throughout the cycle except at estrus and metestrus, while infected rats lose this preference at every stage of the cycle except estrus. Commensurate with the possibility that this was a hormone-dependent effect, infected rats have elevated levels of circulating progesterone, a known anxiolytic [103].

Toxoplasmosis reduces aversion to cat odors in male rats. Relevant proximate mechanisms include the interaction of gonadal testosterone and brain nonapeptide arginine-vasopressin. Specifically, infected male rats exhibit hypomethylation of the arginine vasopressin promoter in the medial amygdala, leading to greater expression of this nonapeptide and greater activation of vasopressinergic neurons after exposure to cat odor; this loss of fear can be rescued by systemic hypermethylation and recapitulated by directed hypomethylation [195]. Furthermore, exogenous testosterone administration into the medial amygdala of uninfected castrated rats recapitulates the reduced fear response to cat odor, while castration post-infection precludes this behavioral change, demonstrating that testosterone acts specifically within the medial amygdala to mediate this parasitic manipulation [196]. Both of these substrates are sexually dimorphic with preferential expression in males, suggesting either absence of behavioral change in females or mediation by analogous neuroendocrine substrates. Studies demonstrate that toxoplasmosis reduces aversion to cat odor in female rats. This change is not accompanied by altered steroid hormones; cannot be rescued by gonadal removal; and does not depend on arginine-vasopressin. Thus, behavioral change in males and females occurs through non-analogous mechanisms that remain hitherto unknown [104].

Typically, female rats demonstrate clear mate choice. Mate preference is driven by the evolutionary need to choose males with heritable parasite resistance and to prevent the transmission of contagious diseases during mating. Thus, females detect and avoid parasitized males. Over evolutionary time scales, parasite-free males plausibly evolve to advertise their status. This arrangement between males and females is obviously detrimental to parasites, especially for sexually transmitted parasites. Yet *T. gondii*, a sexually transmitted parasite, gets around this obstacle by manipulating the mate choice of uninfected females. Males infected with this parasite become more attractive to uninfected females. The ability of *T. gondii* to not only advantageously alter the behavior and physiology of its host but also secondarily alter the behavior of uninfected females presents a striking example of the ‘extended phenotype’ of parasites. *Toxoplasma gondii* also abolishes the innate fear response of rats to cat odor; this likely increases parasite transmission through the trophic route. It is plausible that these two manipulations are not two distinct phenotypes, but are rather part of a single pattern built around testosterone-mediated interplay between mate choice, parasitism and predation [197].

A sexually dimorphic characteristic, the second-to-fourth digit ratio (2D:4D ratio), has been shown to reflect the prenatal concentration of sex steroid hormones and to correlate with many personality, physiological, and life history traits. The correlations are usually stronger for the right than the left hand. Most studies have shown that the 2D:4D ratio does not vary with age or postnatal concentration of sex steroid hormones. Recently, a strong association between the left hand 2D:4D ratio and infection with a common human parasite *T. gondii* has been reported. The confounding effect of toxoplasmosis on the left hand 2D:4D ratio could be responsible for the stronger association between different traits and the right hand rather than the left hand 2D:4D ratio. This confounding effect of toxoplasmosis could also be responsible for the difficulty in finding an association between the 2D:4D ratio and age or postnatal steroid hormone concentration. Analysis of the association between sex and age and 2D:4D ratio in a population of students with and without controlling for the confounding variables of toxoplasmosis and testosterone concentration shows that the relationship between age and sex and 2D:4D ratio increases sharply when toxoplasmosis and testosterone concentration are controlled. Consistent with this, infected men and women have lower 2D:4D ratios (indicating higher prenatal testosterone) compared to uninfected controls, and the 2D:4D ratio negatively correlates with anti-*T. gondii* antibody levels in seronegative subjects, suggesting that individuals with higher prenatal testosterone may have lower natural resistance to infection [21,22,23]. These results suggest that the left hand 2D:4D ratio is more susceptible to postnatal influences and that the confounding factors of toxoplasmosis, testosterone concentration and possibly also age, should be controlled in future 2D:4D ratio studies. Because of a stronger 2D:4D dimorphism in *T. gondii*-infected than in *T. gondii*-free subjects, it is predicted that 2D:4D ratio dimorphism and the right hand/left hand 2D:4D ratio dimorphism will be higher in countries with a high prevalence of toxoplasmosis than in those with a low prevalence [21,22,23].

*Toxoplasma gondii* is a parasite of cats that uses any warm-blooded animalas an intermediate host. It is known to induce shifts in behavior, physiology and even morphology of its intermediate hosts, including humans. The lower second-to-fourth digit ratio (2D:4D ratio) in infected men and women, and higher height in infected men suggest that sex steroid hormones like testosterone could play a role in these shifts. Studies showing portrait pictures of male students to female students reveal that when statistically corrected for age, men with latent toxoplasmosis are perceived as more dominant (*p* = 0.009) and masculine (*p* = 0.052). These results support the idea that the higher level of testosterone could be responsible for at least some of the toxoplasmosis-associated shifts in human and animal behavior [183]. However, a large population-based study using NHANES III data found no association between latent toxoplasmosis and any body measures including height, weight, BMI, or waist-hip ratio in U.S. adults, suggesting that the relationship between *T. gondii* and human morphology may vary across populations or be influenced by unmeasured confounders [198]. An attempt is made to summarize the evidence that the offspring sex ratios (proportion male at birth) of mammals (including man) are causally related to the hormone levels of both parents around the time of conception. Almost all of the cited studies were reported by non-endocrinologists. This being so, it would seem desirable to have comments fromendocrinologists on this topic. The purpose of this article is to elicit such a comment [199].

**Figure 7:**
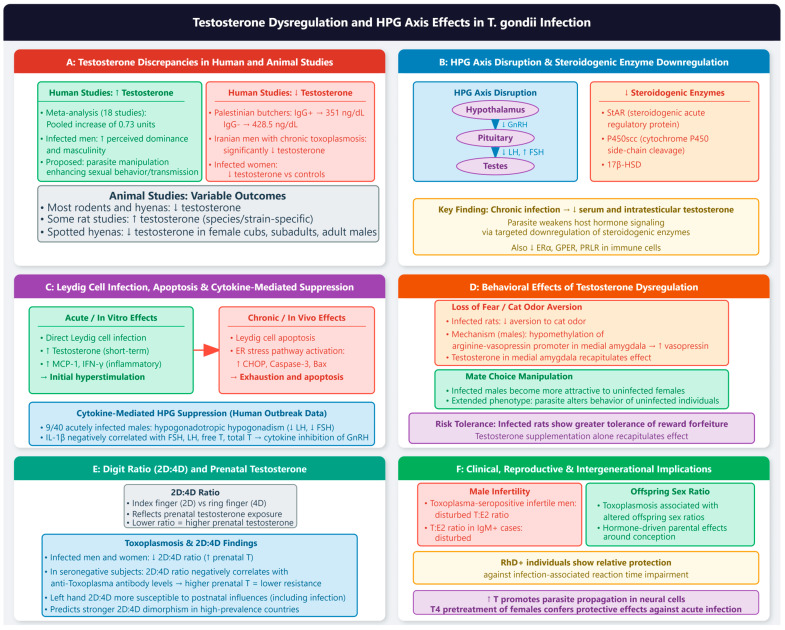
Testosterone dysregulation and HPG axis effects in toxoplasmosis. (**A**) Testosterone discrepancies. Human studies conflict: meta-analysis shows ↑ testosterone in infected males (+0.73 units) with increased dominance/masculinity, but some studies report ↓ testosterone (Palestinian butchers, Iranian men). Infected females show ↓ testosterone. Animal studies mostly show ↓ testosterone, with species/strain variation. (**B**) HPG axis & enzymes. Chronic infection ↓ serum and intratesticular testosterone via downregulation of *StAR*, *P450scc*, and *17β-HSD*. Infected rodents show ↓ LH and ↑ FSH. Human outbreak: acute infection causes hypogonadotropic hypogonadism; IL-1β negatively correlates with LH, FSH, and testosterone. (**C**) Leydig cell apoptosis. Acute infection → hyperstimulation (↑ testosterone, ↑ cytokines). Chronic infection → ER stress-mediated apoptosis (↑ *CHOP*, *Caspase-3*, *Bax*) → cell exhaustion and testosterone decline. (**D**) Behavioral effects. Infected male rats lose cat odor aversion (vasopressin hypomethylation in amygdala; testosterone recapitulates). Infected males are more attractive to uninfected females. Infected rats show greater risk tolerance (recapitulated by testosterone alone). (**E**) Digit ratio (2D:4D). Infected individuals have lower 2D:4D (higher prenatal testosterone). In seronegatives, 2D:4D negatively correlates with antibody levels (higher prenatal T = lower resistance). (**F**) Clinical implications. Infected infertile men have a disturbed T:E2 ratio (8.68 vs. 10.45). Infection alters offspring sex ratios. *RhD+* confers protection. Testosterone has bidirectional effects: it promotes parasite growth in neural cells but protects females against acute infection (Green boxes generally indicate increased/elevated findings, red/pink boxes indicate decreased/pathological findings, blue boxes indicate mechanistic pathways, and yellow boxes highlight key conclusions. ↑ = increased (expression); ↓ = decreased (expression); → = proceed to).

**Figure 8:**
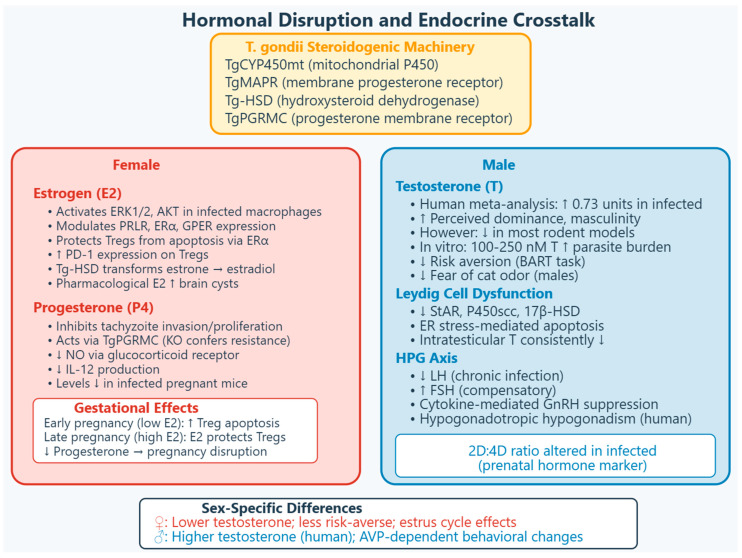
Bidirectional hormonal interactions between host and parasite. *Toxoplasma gondii* possesses intrinsic steroidogenic machinery (*TgCYP450mt*, *TgMAPR*, *Tg-HSD*) while simultaneously manipulating host hormone signaling, leading to sex-specific endocrine disruption (↑ = increased (expression); ↓ = decreased (expression); → = proceed to).

Testosterone modulates infection dynamics bidirectionally. Testosterone at concentrations of 100 and 250 nM significantly increases the number of infected cells and parasite burden 24 and 48 h post-treatment compared to untreated controls in neural cells. Progesterone has no significant effects in the same manner, indicating that testosterone could augment the propagation of *T. gondii* in vitro [108]. Furthermore, testosterone treatment of female mice prior to peroral infection with *T. gondii* tissue cysts markedly reduces intestinal parasite numbers and pathology, demonstrating that testosterone can also confer protective effects against acute infection depending on the context and host sex [165]. Collectively, these findings establish that toxoplasmosis disrupts male endocrine function through multiple mechanisms: direct Leydig cell infection and apoptosis, suppression of steroidogenic enzyme expression, HPG axis dysfunction, and complex cytokine–hormone interactions. The resulting hormonal alterations have consequences extending beyond fertility to encompass behavior, secondary sex characteristics, and potentially intergenerational effects (Table 7, Figure 7 and Figure 8).

#### 3.3.2. Molecular and Genomic Convergence

High-throughput analyses reveal the breadth of *T. gondii*’s impact on reproductive tissue gene expression. In the uterus, infection before pregnancy, after pregnancy, and after implantation differentially affects 4561, 2345, and 2997 genes, respectively, with pathways including anatomical structure development, hormone biosynthesis, and cytokine–cytokine receptor interactions significantly affected [200]. These genomic changes provide a molecular basis for the observed histopathological and functional abnormalities. Cross-tissue transcriptome comparison identified common pathways and specific genes (*Nlrp5*, *Insc*, and *Gbp7*) involved in host response across both testes and uterus, while *Gbp2b* and *Ifit3* represent shared host defense upregulation [17]. This molecular convergence suggests therapeutic targets effective in both sexes. Both uterine and testicular infection share fundamental requirements: breach of physical barriers (blood–testis barrier or placental barrier), evasion of local immune surveillance, and establishment of persistent reservoirs. However, significant differences characterize these processes. Uterine infection typically occurs during acute phases with risk of immediate pregnancy disruption, while testicular infection may persist chronically with cumulative damage to spermatogenesis. The uterus experiences cyclical hormonal fluctuations that may modulate parasite replication, whereas testicular infection faces constant high-testosterone environments that paradoxically may enhance parasite propagation [108]. Transcriptomic analysis comparing infected testes and uterus identified common host response genes including *Nlrp5*, *Insc*, and *Gbp7*, suggesting shared fundamental mechanisms of reproductive damage, while *Gbp2b* and *Ifit3* represent upregulated defense genes in both tissues [17]. This molecular convergence supports integrated therapeutic approaches targeting conserved pathogenic pathways. Chronic toxoplasmosis also has systemic metabolic consequences, significantly altering the expression of hepatic drug-metabolizing enzymes, including multiple cytochrome *P450* genes, which may have important implications for drug efficacy and toxicity in infected individuals [201]. This molecular convergence supports integrated therapeutic approaches targeting conserved pathogenic pathways.

The parasite’s intrinsic steroidogenic capability represents a paradigm shift in understanding host–parasite interactions. Targeted lipidomics detected dehydroepiandrosterone and deoxycorticosterone within *T. gondii*, with *TgCYP450mt* and *TgMAPR* localizing to mitochondria and interacting to support steroidogenesis. Genetic disruption impairs parasite growth, mitochondrial integrity, and virulence, confirming that *T. gondii* is not merely subject to host hormonal influences but possesses endogenous hormone synthesis capacity [19]. Proteomic profiling of human decidual immune cells identified 181 dysregulated proteins, with 11 verified to be associated with trophoblast invasion, placental development, intrauterine fetal growth, and immune tolerance [90].

## 4. Transmission Dynamics and Reproductive Consequences

### 4.1. Vertical Transmission: Timing and Mechanisms

Congenital transmission represents the most clinically significant outcome of maternal infection, with risk varying by gestational timing. Primary maternal infection may cause fetal death in utero or severe sequelae [3,4]. A widevariety of microorganisms are capable of causing fetal infections, and protozoa such as *T. gondii* are prominent among them [202]. In red deer, infection correlates with abortion across all trimesters, indicating a persistent threat throughout gestation [33]. Within-herd seroprevalence at the second pregnancy scan was positively associated with daily abortion rate, and *T. gondii* DNA was detected in uteri, cotyledons, and fetal brains from aborted hinds [33]. The extravillous trophoblast layer represents the initial cellular barrier for placental invasion, with first-trimester explant studies demonstrating significantly greater vulnerability of decidual versus villous regions despite smaller surface area [11]. Using first-trimester human placental explants, researchers demonstrated that the decidual site with extravillous trophoblasts is significantly more vulnerable to infection than the villous region, despite presenting a vastly smaller surface area [11]. Morphological studies in *Calomys callosus* show parasite infection during early blastocyst-endometrial relationship, with p30-containing trails in extracellular matrix highlighting trophoblast defense barrier function [203]. Pregnant *BALB/c* mice infected with a *T. gondii* type II strain have been used to determine how pregnancy interferes with the development of maternal immunity to *T. gondii* and how infection disrupts pregnancy and fetal development. Infected pregnant mice exhibit clinical signs of infection, inflammation and necrosis at the maternal–fetal interface, and decreased serum progesterone levels. In infected mice, there is a clear effect of pregnancy and infection on macrophage cell numbers. However, no differences in parasite load are detected between non-pregnant and pregnant mice, suggesting a pregnancy-dependent mechanism during toxoplasmosis able to interfere with macrophage numbers [100].

Timing of fetal invasion varies by species: in goats, *T. gondii* reaches fetal tissues 11–15 days post-maternal inoculation, with preferential isolation from skeletal muscle, heart, lung, and brain [204,205]. In pigs, transplacental infection appears less common than postnatal infection, but when it occurs, tissue cysts persist in edible organs for several months, with longer persistence in the brain, heart, and tongue [206]. The functional consequences of placental infection are clearly illustrated in pregnant goats experimentally inoculated with *T. gondii*, which abort or deliver stillborn kids 54–73 days post-infection [85]. Ultrasound examination reveals that fetal death precedes expulsion by 1–12 days, with fetal appearance ranging from fresh to mummified depending on the interval. Endocrine profiling demonstrates that infection profoundly disrupts fetal-placental function: inoculated animals exhibit elevated plasma 15-ketodihydro-PGF2α (a prostaglandin metabolite) from approximately day 40 onward, indicating active inflammation and luteolysis, accompanied by a failure of oestrone sulphate to rise normally before parturition [85].

Critically, vertical transmission can occur during chronic infection upon reinfection with different strains, demonstrating that prior immunity may not fully protect the fetus [207]. *Calomys callosus* chronically infected with the *ME-49* strain and reinfected with Brazilian strains showed placental parasite detection and vertical transmission of both strains, with TgChBrUD2 reinfection causing more severe outcomes [207]. However, *BALB/c* mouse studies did not detect congenital transmission of the challenge strain during chronic infection, suggesting host genetic influences on transmission risk [208]. Fatal systemic toxoplasmosis with transplacental transmission has been documented in a pregnant cat and its kittens, providing strong evidence for vertical transmission in feline species. The mother cat presented with dystocia, and despite cesarean section, both surviving kittens died, with *T. gondii* immunopositive reactions observed in multiple tissues and PCR confirming infection [209]. Studies on naturally occurring latent infection in cats and dogs have also documented the distribution of *T. gondii* in various organs, contributing to the understanding of its life cycle and persistence in hosts [210].

In a mouse model of acute infection, congenital transmission was observed in 60.6% of pups when infection occurred 6–11 days after pregnancy, while minocycline treatment reduced transmission to 3.6%. During chronic infection, females exhibited reproductive problems with accentuated hypertrophy of endometrium and myometrium, and only two of 49 females gave birth [211]. Protective immunity against congenital transmission is primarily IFN-γ-mediated; its absence leads to higher uterine and placental parasite loads and more frequent fetal infection, revealing the cytokine’s paradoxical role in protection and immunopathology [212]. Studies in IFN-γ knockout mice demonstrate that *T. gondii* loads are higher in the uterus and placenta of both susceptible (*C57BL/6*) and resistant (*BALB/c*) strains, with fetal infection detected only in IFN-γ KO *C57BL/6* mice, indicating that both IFN-γ and genetic background are important for protective immunity against vertical transmission [212], Table 8.

### 4.2. Sexual Transmission: Biological Plausibility and Controversy

The consistent detection of *T. gondii* in male reproductive tissues raises the critical question of sexual transmission. Biological plausibility is firmly established: parasites colonize testes by day 4 post-infection, replicate significantly, and are present in semen [12,157]. *Toxoplasma gondii* DNA has been detected in ram and human sperm, and infective parasites have been isolated from goat semen for extended periods [137,139,157]. Epidemiological hypotheses suggest that sexual transmission could explain congenital toxoplasmosis cases without identified traditional risk factors [214]. *Toxoplasma gondii* infects about 30% of the human population. The authors hypothesize that sexual transmission of *T. gondii* from men to women could explain many cases of congenital toxoplasmosis where no traditional risk factor is identified, citing the presence of the parasite in semen and epidemiological patterns as supporting evidence [214,215].

However, direct evidence remains limited. An earlier study found no parasites in the semen of husbands of women with *T. gondii*-positive endometrium. Rabbit mating studies failed to demonstrate sexual transmission [142], and infected cats showed no seminal parasites despite disseminated infection [144]. Experimental evidence is mixed. Artificial insemination with contaminated semen successfully transmitted infection in sheep, goats, and rabbits, establishing biological feasibility [133,134,216,217]. Natural mating of ewes with experimentally infected rams resulted in seroconversion and vertical transmission, providing strong evidence for venereal spread in sheep [132]. However, chronic ram infection with subsequent immunosuppression or mating did not result in reactivation or venereal transmission, suggesting latent infection poses a low risk [135]. Experimentally infected rams shed infective *T. gondii* in their semen only transiently (between 14 and 26 days post-infection), leading to the conclusion that venereal transmission is unlikely to be a significant route of spread in ovine toxoplasmosis [131]. *Toxoplasma gondii* was detected in the semen and reproductive tissues of experimentally infected dogs. Artificial insemination of females with this contaminated semen led to seroconversion, fetal reabsorption, and the presence of cysts in offspring, suggesting sexual transmission is possible in dogs [218]. In rams with chronic toxoplasmosis, subsequent immunosuppression or mating with seronegative ewes did not result in reactivation of infection or evidence of venereal transmission, indicating that latent infection poses a low risk for sexual spread [135].

**Figure 9:**
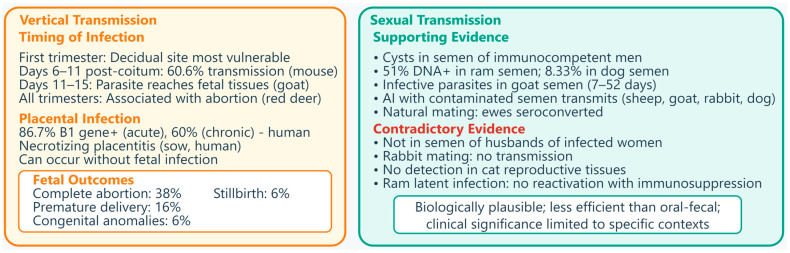
Transmission routes of *T. gondii* with reproductive consequences. Vertical transmission occurs during pregnancy with gestational timing-dependent outcomes. Sexual transmission, while biologically plausible, remains controversial with species-specific evidence. White boxes summarize quantitative fetal outcomes (**left**) and consensus conclusions (**right**).

Laparoscopic artificial insemination of ewes with frozen-thawed semen experimentally contaminated with *T. gondii* tachyzoites successfully established infection, demonstrating that cryopreservation does not eliminate the risk of parasite transmission [134]. In a rabbit model, *T. gondii* was successfully transmitted to females via artificial insemination with contaminated semen, with the vaginal health status of the females not affecting transmission rates [217]. *Toxoplasma gondii* DNA was detected intermittently in the semen and reproductive organs of experimentally infected goats, suggesting that male goats could serve as a source of venereal transmission [219,220]. Goats inseminated vaginally with semen containing *T. gondii* tachyzoites became infected, leading to seroconversion, detection of parasite DNA in blood, and embryonic reabsorption, confirming the potential for vaginal transmission [216]. Sheep inseminated with fresh semen contaminated with *T. gondii* tachyzoites became infected, with higher parasite doses resulting in 100% seroconversion and evidence of embryonic reabsorption, confirming that contaminated semen is an effective route of transmission [133]. Despite a 22.6% seroprevalence in a large cohort of cats and dogs undergoing neutering, no *T. gondii* DNA or viable parasites were detected in their reproductive tissues, suggesting that natural infection may not always lead to colonization of these organs [159]. Current consensus holds that while biological plausibility for sexual transmission is established, it represents a less efficient route than oral–fecal transmission from felids (via oocysts) or oral transmission via tissue cysts from infected intermediate hosts. Clinical significance may be limited to specific contexts such as serodiscordant couples during pregnancy or artificial insemination with contaminated semen, Table 9, Figure 9.

## 5. Therapeutic Strategies and Future Directions

### 5.1. Conventional Antiparasitic Therapy

Standard therapy combining pyrimethamine and sulfadiazine achieves parasite suppression but faces limitations including bone marrow suppression, megaloblastic anemia, leukopenia, and granulocytopenia, and poor penetration of reproductive sanctuaries [220,221]. The failure of sulphadimidine to prevent sperm quality deterioration in infected rams, despite partial immune suppression, highlights the blood–testis barrier’s therapeutic challenge [137]. Nitrofurantoin, alone or combined with spiramycin, reduces parasite burden and uterine inflammation with good inhibitory effects on hepatic, renal, and uterine inflammatory processes [222]. The combination of diclazuril and atovaquone shows synergistic pregnancy protection [223]. Early studies with sulfamonomethoxine in experimentally infected pigs provided foundational knowledge on drug distribution and efficacy in controlling toxoplasmosis. Monensin, an ionophore antibiotic, significantly reduces lamb losses in infected ewes (16.7% versus 55.2% in untreated controls) and results in heavier lambs, possibly due to a lesser “weight” of infection within the gravid uterus. Monensin alone caused no discernible problems [224].

### 5.2. Immunomodulatory Approaches

Given that reproductive pathology often stems from immunopathology rather than direct parasite cytopathic effect, immunomodulatory strategies targeting host responses show particular promise. TGF-β1 administration improves pregnancy outcomes by decreasing dNK cell cytotoxicity via *NKG2D*/DAP10 pathway modulation. IL-10 reduces Treg apoptosis and improves outcomes, with recombinant IL-10 downregulating caspase expression [57]. The immunomodulatory potential of IL-10 in *T. gondii*-infected trophoblasts was investigated, and it was demonstrated that IL-10 treatment of infected human trophoblasts and BeWo cells decreased *HLA-G* expression compared to untreated infected cells, while no change was observed in uninfected cells treated with IL-10. Critically, IL-10 treatment reduced apoptosis levels in infected trophoblasts, with corresponding decreases in *caspase-3* and *caspase-8* expression and increased *c-FLIP* levels. These findings suggest that IL-10 regulates *HLA-G* expression and reduces apoptosis in *T. gondii*-infected trophoblasts, potentially contributing to improved pregnancy outcomes when infected women receive IL-10 treatment [57,176].

IOP demonstrates multi-target protective effects in both sexes. In pregnant mice, IOP reduces abortion rates, preserves progesterone and estriol, mitigates oxidative stress, and rebalances the immune environment by inhibiting pro-inflammatory cytokines (TNF-α, IL-6, IFN-γ, IL-1β, IL-17A) and promoting anti-inflammatory mediators (IL-10, TGF-β) via *TLR4/NF-κB* signaling and Th17/Treg equilibrium restoration [80]. In males, IOP improves spermatogenic capacity, restores testosterone, LH, and FSH levels, upregulates steroidogenic enzymes (*StAR*, *P450scc*, *17β-HSD*), enhances antioxidant defenses via Nrf2 (increasing SOD, GSH, HO-1, NQO-1 while decreasing MDA and NO), and suppresses apoptosis through *PI3K*/*AKT*/*mTOR* pathway activation [171]. Ginseng polysaccharide similarly mitigates testicular injury by reducing inflammation (*TLR4*-*P2X7R/NLRP3* pathway), inhibiting apoptosis (ER stress *PERK*/*eIF2α* pathway), and restoring hormonal levels [161].

### 5.3. Natural Products and Emerging Therapies

Natural products including propolis and wheat germ oil, particularly in combination, decrease parasite load and restore histopathological damage in the brain, uterus, and kidney in both acute and chronic models [225,226]. The combination treatment effectively restored histopathological changes observed in the brain, uterus, and kidney [225]. *Copaifera* oleoresins impair parasite proliferation in trophoblasts and villous explants through immunomodulation and direct antiparasitic action, with irreversible concentration-dependent antiparasitic action and parasite cell cycle arrest in the S/M phase [227]. Prenylated chalcones cause ultrastructural tachyzoite damage and modulate cytokine profiles by increasing IL-8 and downmodulating MIF and ROS levels in BeWo cells, and downregulating TNF-α release in villous explants [228]. S-methylcysteine combined with spiramycin mitigates hormonal imbalances and promotes tissue recovery in the uterus and ovary in female rats [88]. Nanomedicine approaches may overcome blood–tissue barrier limitations. Biogenic silver nanoparticles (AgNp-Bio) reduce tachyzoite viability without Leydig cell cytotoxicity at low concentrations, decreasing infection rates and proliferation while activating microbicidal and inflammatory mechanisms [221]. Among the morphological and ultrastructural changes, AgNp-Bio induces a reduction in the number of intracellular tachyzoites and causes changes in the tachyzoites with accumulation of autophagic vacuoles and a decrease in the number of tachyzoites inside the parasitophorous vacuoles [221].

### 5.4. Vaccination Strategies

Despite decades of research, no commercial vaccine exists for human toxoplasmosis, with the only licensed product being for sheep to prevent ovine abortions [18,20]. The parasite’s complex life cycle, ability to form latent tissue cysts, and sophisticated immune evasion mechanisms have frustrated conventional vaccine approaches. The urgency is further underscored by the observation that cerebral toxoplasmosis remains a common opportunistic infection in AIDS patients, where delayed diagnosis frequently leads to neurological impairment and death [229].

#### 5.4.1. Foundational Lessons from Veterinary Vaccinology

The development of effective vaccination strategies for toxoplasmosis has been significantly informed by decades of research in animal models, particularly regarding the prevention of reproductive failure. Early studies demonstrated proof-of-concept using heterologous parasites: *Hammondiahammondi* oocyst vaccination of goats resulted in four of five vaccinated does giving birth to healthy kids despite subsequent *T. gondii* challenge, although *T. gondii* was still isolated from tissues of all kids born to vaccinated does [204,205]. Similar protection against abortion and neonatal death was observed in goats vaccinated with *H. hammondi* oocysts, though again fetal infection was not completely prevented [230]. Studies in sheep established that vaccination suppresses parasite dissemination in lymph and therefore to other sites, including the gravid uterus. This work culminated in the commercial development of the live attenuated S48 strain (Toxovax^®^), which increased viable lamb births, reduced placental damage, and provided protection lasting at least 18 months [231]. Mechanistic investigations revealed that toxoplasmosis in pregnant ewes triggers an early rise in prostaglandin metabolite levels followed by declines in both progesterone and oestrone sulphate—hormonal patterns that correlate with adverse pregnancy outcomes [101]. Critically, vaccination with immunostimulating complexes (iscoms) prior to infection markedly preserved normal endocrine profiles in challenged animals compared to unvaccinated controls, suggesting that immune modulation can protect the fetal-placental unit from hormone-mediated pregnancy failure [101]. These foundational insights have guided contemporary vaccine development, which now encompasses an expanding arsenal of platforms including inactivated vaccines, virus-like particles (VLPs), viral vectors, DNA constructs, mRNA formulations, and transmission-blocking strategies.

#### 5.4.2. mRNA Vaccines: A Promising Frontier

A recent study presents the exploration of an mRNA-based vaccine against *T. gondii*, employing reverse vaccinology to design a multi-epitope construct targeting *MIC1*, *MIC3*, *ROP29*, and *SAG1* proteins. This in silico approach achieved 100% predicted global population coverage, with docking analyses revealing high binding affinities to *TLR-2* and *TLR-4* receptors (energy scores of −1000.5 and −1008.6 kJ/mol, respectively). The refined vaccine demonstrated favorable biophysical properties with a Ramachandran score of 80.9% and a Z-score of −7.55 [232]. Building on this foundation, another study directly compared unmodified versus nucleoside-modified mRNA constructs expressing *GRA1* protein in HEK293T cells. Surprisingly, unmodified constructs with extended poly-A tails yielded significantly higher protein expression than their nucleoside-modified counterparts. Both formats produced proteins that reacted strongly with *T. gondii* IgG seropositive sera, suggesting that the immunogenicity of unmodified mRNA may be sufficient without nucleoside modification—a finding with important manufacturing implications [233]. Similarly, optimized *ROP6* mRNA constructs have been shown to successfully expressimmunogenic protein in cell culture, with recombinant protein strongly reacting with sera from infected mice (*p* = 0.0003) [234]. The most clinically advanced mRNA candidate involves a quadrivalent self-amplifying mRNA-LNP vaccine incorporating *ROP18*, *TGME49_237490*, *TGME49_268230*, and *MIC13* [235]. This construct induced sustained antibody titers for at least 10 weeks post-vaccination and achieved 60–80% survival against lethal challenges with *RH*, *ME49*, and *WH6* strains. Notably, vaccinated mice showed dramatically reduced tissue cyst burdens (72.5% reduction, *p* < 0.05) following PRU oocyst challenge, addressing both acute and chronic disease [235].

#### 5.4.3. Live-Attenuated Vaccines: Efficacy with Safety Concerns

Live-attenuated vaccines consistently demonstrate the most robust protection but raise safety concerns. The *PruΔpp2a-c* mutant was shown to elicit strong cellular (IL-2, IL-4, IL-10, IL-12, IFN-γ) and humoral (IgG, IgG1, IgG2a) responses in mice and, importantly, reduced feline oocyst shedding by 94.5% (*p* < 0.001). This dual efficacy in both intermediate and definitive hosts represents a significant advance for transmission-blocking strategies [236]. Similarly, the *PruΔUrm1* mutant provided 100% protection against lethal type I *RH* challenge while exhibiting markedly reduced pathogenicity and significantly elevated cytokine levels including TNF-α, IFN-γ and IL-10 [237]. A C2 domain-containing protein deletion mutant (*ME49Δ203240*) was found to protect mice against lethal challenge with type I, II, and III strains, eliciting high levels of specific IgG antibodies and key cytokines (IFN-γ, TNF-α, IL-12) [238]. Additionally, *UBLCP1*-deficient parasites exhibited collapsed mitochondria, decreased mitochondrial membrane potential, and compromised growth; immunization with the deletion mutant protected mice against challenges with virulent PRU and VEG strains [239]. A direct comparison of gamma-irradiated live tachyzoites (0.25 kGy) versus killed preparations (1.5 kGy) in a murine model demonstrated that the live attenuated vaccine achieved 57.1% long-term survival (six months) with 99.8% infection reduction and complete absence of tachyzoites in liver impression smears, whereas the killed vaccine offered minimal protection. The live vaccine triggered significantly elevated levels of IFN-γ, IL-12, and IL-17, and increased percentages of CD4+ and CD8+ T-lymphocytes [240]. However, regulatory hurdles for live vaccines in humans remain substantial, directing attention toward safer platforms, particularly given that *T. gondii*’s capacity to disrupt programmed cell death pathways and establish chronic infections while avoiding immune detection poses fundamental challenges for vaccine development [241].

#### 5.4.4. Inactivated Vaccines: Adjuvant Innovation

An inactivated vaccine based on a low-passage type III isolate (*TgPigSP1*) was evaluated in pregnant sheep, achieving 100% and 78% viable gestations in two trials compared to 50% in controls [242]. The vaccine, adjuvanted with QuilA^®^, induced both cellular and humoral immune responses without adverse gestation effects [242]. The same vaccine platform, when tested in pigs, reduced tissue cyst burden by ≥95% in target muscles, with viable *T. gondii* cysts undetectable in at least 50% of vaccinated animals as confirmed by bioassay [243]. Colloidal manganese salt (MnJ) adjuvant has been shown to significantly enhance an inactivated vaccine’s efficacy, triggering powerful innate immunity that increased CD4+ and CD8+ T cells secreting IFN-γ and enhanced CD8+ central and effector memory T cell generation. The survival rate reached 50% against acute infection—a 40% improvement over vaccine alone—while brain cyst burden was reduced by 90.77% during chronic infection [244]. A novel low-temperature inactivation method hasbeen introduced, achieving 10% protection with inactivated vaccine alone, but 50% and 70% protection when combined with adjuvants HA201 and HA203, respectively. The approach preserved antigen integrity while ensuring biosafety, addressing a key limitation of traditional chemical inactivation that can damage protective epitopes [245].

#### 5.4.5. Virus-like Particles and Viral Vectors

The VLP platform has shown remarkable versatility. Influenza matrix protein-based VLPs displaying *GRA14* achieved 100% short-term survival and 83% long-term protection (32 weeks post-vaccination) against lethal *ME49* challenge [246]. Vaccinated mice exhibited elevated *T. gondii*-specific IgG, IgG1, and IgG2a responses, increased antibody-secreting plasma cells, germinal center B cells, memory B cells, and both Th1 (IFN-γ) and Th2 (IL-4, IL-5) cytokines [246]. *GRA5* VLPs induced parasite-specific IgG in serum and both IgG and IgA in brain tissues, and notably suppressed IFN-γ and IL-6 production in the brain, leading to significantly reduced inflammation and decreased cyst counts—suggesting a mechanism that limits immunopathology while controlling infection [247]. Similarly, *GRA7* VLPs conferred 100% survival with a marked reduction in cerebral pro-inflammatory cytokines and parasite cyst burden [248]. Recombinant vaccinia viruses expressing *MIC8*, *AMA1*, or *RON4* have been compared, with all three providing 100% survival against *ME49* challenge, with *MIC8-rVV* eliciting the strongest CD8+ T cell and B cell responses and achieving 89.6% reduction in brain cyst counts [249]. A subsequent heterologous prime-boost strategy (rVV prime, VLP boost) further improved efficacy, with the rVV + VLP group showing higher antibody-secreting cell responses, memory B cells, and CD4+/CD8+ T cells compared to homologous VLP vaccination [249]. Recombinant vaccinia viruses expressing *MIC8* and *AMA1* together produced significant increases in *T. gondii*-specific IgG, robust CD4+ and CD8+ T cell activation, memory B cell expansion, and 89.6% cyst reduction [250]. Additionally, *ROP4*-expressing recombinant vaccinia virus induced parasite-specific serum antibody responses as early as 3 weeks post-immunization, with enhanced mucosal IgA in intestines upon challenge. Immunization preserved T cell and germinal center B cell populations, mitigated neuroinflammation, reduced tissue cyst formation, and ensured survival [251]. A comprehensive review of VLP applications against protozoan parasites notes that VLP vaccines targeting *T. gondii*, *Plasmodium* spp., and *Leishmania* spp. have the potential to make significant contributions to public health, though antigenic diversity and short-lived immunity remain challenge [252].

#### 5.4.6. Subunit and DNA Vaccines

Multiple groups have pursued DNA vaccine strategies with various antigen combinations. *GRA28* and *GRA83* with *IL-28B* as a molecular adjuvant demonstrated that the cocktail vaccine with adjuvant significantly improved survival and reduced cyst formation compared to single antigens. Co-administration of pVAX-*IL-28B* further augmented vaccine-induced immunity, enhancing both cellular and humoral responses in a type I IFN signaling-independent manner consistent with type III IFN biology [253]. A *ROP6*/*MIC12* bivalent DNA vaccine reduced brain cyst burden by 56.6% and prolonged survival following lethal *RH* challenge, outperforming single-gene formulations. The vaccine elicited elevated IgG2a/IgG1 ratios, enhanced cytotoxic T lymphocyte activity, increased CD4+ and CD8+ T cell populations, and elevated production of IFN-γ, IL-12, and IL-2. Similarly, a five-gene DNA vaccine cocktail (*ROP5*, *ROP18*, *GRA7*, *GRA15*, *MIC6*) with *IL-24* adjuvant significantly increased serum IgG levels, Th1 cytokine production, and CD4+/CD8+ T cell proportions, leading to extended survival and reduced brain cyst counts [254]. *IMP1* DNA vaccine with IL-12 adjuvant induced elevated IgG1 and IgG2a antibodies, strong lymphoproliferative responses, and higher IFN-γ and IL-4 production, with prolonged survival times compared to controls (*p* < 0.05), though protection remained modest. A multi-epitope DNA vaccine encoding *ROP21* and *ROP29*, which significantly increased total IgG antibodies (122.16 ng/mL) and IFN-γ (12.37 pg/mL) with a predominant IgG2a response, prolonged survival to 14 days, and reduced brain cyst burden [255]. A *ROP18* DNA vaccine in domestic catsresultedin vaccinated animals shed 53.3% fewer oocysts than controls and produced specific IgG antibodies, though statistical significance was not achieved for oocyst reduction (*p* > 0.05) [256].

#### 5.4.7. Nanoparticle Delivery Systems

Several studies have focused on optimizing antigen delivery through nanoparticle formulations. Encapsulation of TgGAP45 in PLGA nanoparticlesinduced stronger immunoprotection against acute toxoplasmosis than oil adjuvants (Montanide ISA 660 VG and 206 VG). The PLGA formulation induced a mixed Th1/Th2 immune response and provided the strongest reduction in parasite burden in spleen and heart tissues [257]. Similarly, pVAX1-*TgIMC1*-loaded PLGA and chitosan nanospheres enhanced humoral and cellular immune responses compared to naked DNA, significantly increasing specific IgG levels and cytokine production (IFN-γ and IL-17), with PLGA nanospheres exhibiting superior protection against acute toxoplasmosis, while chitosan nanospheres offered advantages in antigen stability and delivery [253]. Niosomes as vehicles for excretory/secretory antigensachieved 85% and 90% parasite reduction in liver and spleen, respectively—superior to conventional adjuvants. Niosome-formulated vaccine efficiently reduced inflammation and parenchymal injury in the liver with intense *iNOS* immunostaining expression, and significantly increased anti-*T. gondii* IgG, CD4+ and CD8+ percentages, and serum IFN-γ levels [258]. Cationic DOTAP liposomes with imiquimod adjuvant induced strong Th1 responses with elevated IgG2a and IFN-γ secretion (*p* < 0.0001), significantly improving survival against *RH* challenge compared to control groups. A systematic review of liposomal nanocarrier systems against protozoan diseases confirmed that such platforms effectively modulate immunological profiles by elevating protective cytokines including IFN-γ, TNF-α, IL-12, and IL-17 [259].

#### 5.4.8. Veterinary Applications and One Health Implications

Several studies specifically addressed veterinary applications with direct public health relevance. The inactivated vaccine platform demonstrates the feasibility of controlling *T. gondii* in livestock, thereby reducing zoonotic transmission through meat consumption. The vaccine was safe in both murine models and piglets, with an absence of local reactions observed in piglets, and induced robust humoral and cellular immune responses evidenced by elevated IgG and IFN-γ levels [242,243]. Immunization of cats with recombinant *GRA12* protein formulated with ISA 201 adjuvant achieved a 20.1–27.9% reduction in oocyst shedding (*p* < 0.05) and extendedmedian survival from 19 days (controls) to 60 days (vaccinated), inducing mixed Th1/Th2 responses with an IgG1/IgG2a ratio > 1, elevated IFN-γ, IL-2, TNF-α, and IL-4 levels. Parasite burdens in the brain, heart, lungs, liver, and spleen were significantly reduced in vaccinated cats (*p* < 0.01) [260].

An innovative using a plant-based vaccine approach involved orally administering leaves expressing *SAG1* fused to *Arabidopsis thaliana* Hsp90 to lambs. While the subcutaneous recombinant vaccine (produced in *E. coli*) induced higher antibody titers (∼4-fold more than controls), the oral plant vaccine uniquely increased IFN-γ serum levels post-challenge (~3-fold more than controls). Both vaccination strategies reduced histopathological lesions by approximately 80% compared to unvaccinated challenged animals. The study used the recombinant Gra4-Gra7 protein as an acute infection marker, confirming that vaccinated lambs had lower serological reactivity correlating with reduced lesion severity [261]. The acute immune response in sheep following immunization with tachyzoites or parasite-derived glycoconjugates, finding that infected sheep developed specific IgM antibodies from day 4 and IgG against glycoconjugates from day 12 post-infection. Glycoconjugate-immunized sheep produced IgM against lysate antigens from day 4 and IgG from day 12, with flow cytometry revealing a significant increase in circulating CD8+ T cells on day 60 [262].

#### 5.4.9. Immunoinformatics and Rational Design

Characterization of *TgSPATR* identified 534 amino acids with moderate thermostability (aliphatic index 65.71) and hydrophilic nature (GRAVY-0.507). The protein contained 120 post-translational modification sites, with refined 3D models achieving 93.8% of residues in favored Ramachandran regions and an ERRAT score increasing from 89.557 to 95.046 after refinement. Virtual immune simulation suggested that three injections would elicit both humoral and cellular immune responses [263]. Multi-criteria decision-making tools have been applied (TOPSIS, VIKOR, MABAC) to rank all 8000+ *T. gondii* proteins for vaccine potential, integrating Vacceed exposure scores, B and T cell epitope metrics, and physicochemical properties. Top-ranked proteins included novel, uncharacterized membrane transporters and proteins associated with RNA metabolism. Conformal prediction intervals provided confidence estimates for each rank [264]. Other notable computational studies include characterization of *CDPK8* [265], which identified notable surface accessibility, flexibility, and antigenicity indices; *SABP1* [266], a 315-residue protein with antigenicity score 0.46 and high solubility (0.783); perforin-like proteins *PLP1* and *PLP2* [267], both showing good antigenicity (0.7021 and 0.5701) with numerous post-translational modification sites; *ROP41* [268], with secondary structure containing 33.47% alpha-helix and 49.18% random coil; and *TgAMA1* [269], a 569-amino acid protein with refined model showing over 97% of residues in favored Ramachandran regions. *SRS67* and *SRS20A* genes were cloned and analyzed, successfully expressing recombinant proteins in *E. coli* and confirming their immunogenicity [270]. A secreted form of *SAG2* protein has been designed, identifying appropriate signal peptides for fusion and translocation into the extracellular environment [271]. The multi-epitope vaccine design approach has been further advanced by a construct combining *ROP5*, *ROP7*, and *SAG1* epitopes, within silico analyses confirming antigenicity (VaxiJen score: 0.96), non-allergenicity, solubility (GRAVY index: 0.45), and physicochemical stability (instability index: 32.14; aliphatic index: 78.3). Molecular docking demonstrated high-affinity binding to murine *TLR4*, and vaccination reduced cerebral cyst burden by 76% (*p* < 0.01) [272]. Five multi-epitope vaccine candidates using membrane protein-derived epitopes have been designed using membrance protein-derived epitopes, with constructs containing RS-09 adjuvant (Toxo-App), IFN-γ (Toxo-Apfn), and 50S ribosomal protein (Toxo-Ribos) showing the highest predicted immunogenicity [267,273]. A chimeric RMS immunogen from *ROP18*, *MIC4*, and *SAG1* domains (545 amino acids; MW 58.8 kDa) has been designed, with secondary structure containing 21.28% alpha-helix, 24.59% extended strand, and 54.13% random coil [274].

#### 5.4.10. Mechanistic Insights Supporting Vaccine Development

Understanding parasite biology continues to inform vaccine design. *TgEfhc3*, an EF-hand domain-containing protein with the highest Ca^2+^-binding affinity among *T. gondii* calcium-binding proteins, is essential for tachyzoite proliferation, egress, and invasion. Genetic deletion severely impaired these processes, and recombinant protein immunization induced high levels of anti-*T. gondii* IgG, enhanced Th1/Th2 cytokine production (IL-4, IFN-γ, IL-10), stimulated robust CD4+ T-cell proliferation, and significantly prolonged survival time following acute infection [275]. *ROP6* expressed in *Saccharomyces cerevisiae* INVSc1 cells, showing that Freund’s-adjuvanted *rROP6* induced strong CD8+ T lymphocyte IFN-γ secretion (*p* < 0.05) and provided significant protection with all vaccinated mice surviving challenge and showing significantly reduced tissue cysts and *T. gondii* DNA (*p* < 0.01; *p* < 0.0001). A comparison of cell wall disruption methods for *S. cerevisiae* expressing *rROP6* found that microfluidizer and acid-washed glass bead methods achieved superior efficiency for large-scale processing, while Y-PER reagent offered better protein stability with reduced multimerization and degradation [276]. Glutaredoxin 5 (*Grx5*) has been identified as essential for oocyst formation and sporulation, with knockout reducing oocyst production in cats by approximately 70% and sporulation rate by 50%. Tachyzoites showed no defects in growth or virulence, and neither in vitro nor in vivo tachyzoite-to-bradyzoite differentiation was affected, indicating that *Grx5* plays a predominant role specifically within the feline host and during the external environmental stage of sporulation—providing a crucial molecular target for transmission-blocking vaccine development [277]. Quantitative proteomic analysis of the *T. gondii* cytoskeleton identified 623 proteins including 30 IMC proteins, 34 cytoskeleton proteins, and 14 uncharacterized proteins. *ROP8* emerged as the most promising vaccine candidate based on epitope prediction [278]. Immune-proteomics identified 313 antigenic proteins recognized by sera from patients with acute and chronic toxoplasmosis, including 63 recognized by IgM and 250 by IgG antibodies, with bioinformatic analyses enabling selection of highly immunogenic proteins [279].

Characterization of the N-glycoproteome of *T. gondii* tachyzoites using lectin enrichment and mass spectrometry identified over 100 N-glycoproteins with glycosylation sites and N-glycan compositions. Identified glycoproteins included known virulence factors and vaccine candidates, providing ground knowledge for rational vaccine design [280]. *SRS67* and *SRS20A* proteins, both containing signal peptides, exhibit favorable immunogenicity with multiple B-cell, Th-cell, and CTL epitopes. *SRS67* had 7 dominant B-cell epitopes, 10 Th epitopes, and 2 CTL epitopes; *SRS20A* had 8 B-cell, 20 Th, and 3 CTL epitopes. *MIC13* possesses 70 phosphorylation sites and five acetylation sites, with secondary structure containing 27.99% alpha-helix, 16.45% extended strand, and 55.56% random coil [270]. *GRA10* has 192 post-translational modification sites and confirming its immunogenic but non-allergenic nature [281].

#### 5.4.11. Novel Platforms and Emerging Concepts

*T. gondii*-derived cell membrane-derived nanovesicles (TgCMNVs) retain unique features including abundant GPI-anchored SRS proteins, phosphatidylthreonine-rich lipids, and an editable genome enabling versatile engineering. TgCMNVs show promise for immunomodulation, attenuation of tissue injury, cancer immunotherapy, and self-adjuvanting vaccine design [282]. *Bacillus subtilis* spores displaying *GRA12* (rBS-*GRA12*) significantly increased splenocyte proliferation, IgG and IgG2a titers, IFN-γ, IL-12, and IL-4 production, and secretory sIgA levels compared to controls, and immunized mice exhibited significantly lower brain and liver parasite loads and longer survival times when challenged with acute toxoplasmosis [283]. A large-scale evaluation of VXN-Toxo, an intranasal vaccine composed of maltodextrin nanoparticles conjugated with inactivated *T. gondii*, was administered to 784 animals representing over 58 species across 20 zoos. Retrospective mortality data revealed an overall 96.7% reduction in toxoplasmosis-associated deaths post-vaccination, demonstrating consistent broad-spectrum protection against different *T. gondii* strains in a wide array of captive wildlife species [284]. Despite these advances, significant hurdles remain. Acomprehensivereview of challenges, including antigenic diversity, short-lived immunity, regulatory barriers, and limited funding, notes that emerging technologies, including systems vaccinology, reverse vaccinology, and vectored delivery platforms, are paving the way for more targeted and effective vaccination [285]. The comparative efficacy across platforms is instructive. Live-attenuated vaccines consistently provide the strongest protection but face safety concerns for human use, particularly in immunocompromised populations who need protection most. mRNA and VLP platforms offer superior safety profiles with impressive efficacy in animal models, though long-term durability remains to be established. The 4x-mRNA-LNP vaccinemaintainsantibody responses for 10 weeks post-vaccination, which is encouraging, but human data are lacking [235].

Adjuvant selection emerges as a critical determinant of efficacy. The success of MnJ [244], niosomes [258], PLGA nanoparticles [257], and liposomal formulations [259,286] suggests that formulation science may be as important as antigen selection. The finding that NPL/*T. gondii* formulation specifically triggers existing Th1 memory in seropositive donors, raises intriguing possibilities for therapeutic vaccination to prevent reactivation [287]. The potential for parasite-based cancer immunotherapy represents an unexpected but promising direction. *Toxoplasma gondii* immunomodulators exhibit antineoplastic activity against human colorectal and hepatocellular carcinoma cells, with anti-*T. gondii* antibodies showing 50.43% inhibition of HT-29 colorectal cancer cells [288]. Molecular mimicry between *T. gondii* and cancer cells has been documented, though *T. gondii* antibodies did not react with MCF-7 breast cancer or A549 lung cancer cell extracts, suggesting species-specific cross-reactivity patterns [289].

#### 5.4.12. Post-Vaccination Protection Against Reproductive Toxoplasmosis

The prevention of *T. gondii*-induced reproductive failure through vaccination has been pursued for nearly four decades, with markedly different outcomes depending on the vaccine platform. Live vaccines have consistently succeeded where killed vaccines failed. Early attempts using killed, disintegrated tachyzoite vaccines in ewes actually worsened lambing outcomes: twice-vaccinated ewes had significantly lower lambing percentages than unvaccinated controls following challenge [290]. This failure established that effective immunity against *T. gondii* abortion requires live vaccination. The live attenuated S48 strain (later commercialized as Toxovax^®^) proved highly effective. Vaccinated ewes challenged at mid-gestation produced 72–81% viable lambs compared to only 18% in unvaccinated controls [291,292]. Critically, this protection lasted at least 18 months after a single pre-mating immunization [291]. The mechanism involves preservation of normal endocrine function: infected ewes show characteristic rises in prostaglandin metabolites followed by falls in progesterone and oestrone sulphate, but vaccinated animals maintain significantly more normal hormonal patterns [101]. Toxovax^®^ remains the only commercially licensed vaccine for ovine toxoplasmosis worldwide [231,293].

Alternative live candidates have since emerged. The *Mic1-3KO* strain proved as effective as S48 in sheep, with 62–91% of lambs viable following challenge compared to 0% in unvaccinated ewes [294]. The *TS-4* temperature-sensitive mutant showed no transplacental transmission in mice and was avirulent in pregnant adults, though it caused disease in neonates [295]. More recent deletion mutants including *RHΔtkl1* and *RHΔgra17Δnpt1* have protected mice against congenital infection, with vaccinated dams producing viable litters while all unvaccinated dams aborted [296,297]. Subunit and DNA vaccines have shown only partial success. Recombinant *SAG1* protein protected 66–86% of guinea pig fetuses from congenital transmission [298]. A DNA cocktail encoding *GRA4* and *SAG1* with *GM-CSF* increased live births in mice from 1.1 to 4.3 per litter, though vertical transmission was not completely prevented [299]. The *ROP6*/*MIC12* bivalent DNA vaccine reduced brain cysts by 57% and prolonged survival, but protection remained partial [254]. Importantly, *T. gondii* vaccination does not cross-protect against *Neospora caninum*. Ewes immunized with Toxovax^®^ suffered 100% fetal death following *N. caninum* challenge, despite evidence of cross-reactive cellular immunity [300]. However, the converse approach—immunization with attenuated *T. gondii mic1-3KO*—did protect mice against lethal *N. caninum* infection, with 70–80% survival compared to 30% in controls [301].

### 5.5. Drug Safety Considerations

Clinicians must remain aware of drug interactions: tamoxifen exacerbates chronic infection and increases uterine pathology with severe endometrial necrosis [302]. Tamoxifen treatment during chronic toxoplasmosis increased parasite burden and induced severe histopathological changes including multiple *T. gondii* tissue cysts in the lumen of proximal convoluted tubules with complete necrosis, multiple tissue cysts in hepatic parenchyma, clusters of intracellular tachyzoites in the endometrial lining epithelium associated with severe endometrial necrosis, and hemorrhages in pia mater, with severity increasing with treatment duration [302]. Pyrimethamine causes testicular developmental delays in neonatal rats, with delays in testicular development, reduced cell proliferation, and increased apoptosis, effects that are partially mitigated by folic acid supplementation [303]. Even when pyrimethamine is highly specific against parasites, it may provoke neutropenia in patients apart from other complications, which usually justify its suspension. Moreover, medication against congenital toxoplasmosis coincides with the proliferation stage of Sertoli and germ cells, highlighting potential side effects of this common anti-*T. gondii* drug [303].

**Figure 10:**
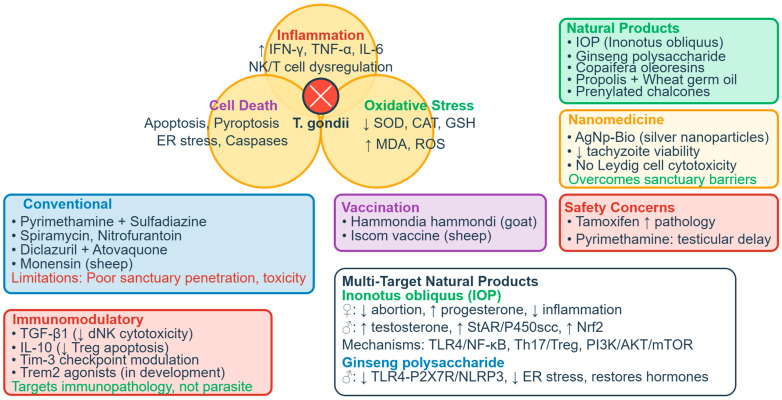
Integrated therapeutic approaches targeting the pathogenic triad of inflammation, oxidative stress, and cell death in both sexes. Conventional antiparasitics, immunomodulators, natural products, and nanomedicine offer complementary strategies for reproductive toxoplasmosis. The central red circle with white cross represents *T. gondii*. The three surrounding yellow circles depict the core pathogenic triad: Inflammation (top), Oxidative Stress (bottom-right), and Cell Death (bottom-left), illustrating how the parasite drives all three interconnected pathological processes. ↑ = increased (expression); ↓ = decreased (expression).

Treatment with flunixin meglumine, a prostaglandin synthesis inhibitor, could not prevent abortion in infected ewes despite depressing prostaglandin release and eliminating fever, suggesting that the infectious process itself was not sufficiently affected by anti-inflammatory therapy alone [304]. The aim of examining the endocrinological response (15-ketodihydro-PGF2α, progesterone, and oestrone sulphate) of pregnant ewes constantly treated with flunixin meglumine (FM) after infection with *T. gondii* reveals that FM can neither completely inhibit prostaglandin release during abortion nor the physiological change in the hormone before parturition, even though it depresses prostaglandin release before abortion or parturition and eliminates fever. The infectious process caused by the organism is probably not affected. FM treatment alone has no observed negative effects on pregnant ewes and their fetuses [304], Table 10, Figure 10.

## 6. Broader Clinical and Epidemiological Implications

The impact of *T. gondii* extends beyond direct reproductive pathology. Spontaneous abortion is estimated to occur in 15–20% of all clinical pregnancies. Among the infective factors, *T. gondii* is difficult to analyse since serology is not sufficient without real proof of endometrial colonization [109]. Recurrent pregnancy loss (RPL), affecting approximately 5% of couples, remains idiopathic in up to 50% of cases, and environmental triggers such as toxoplasmosis are increasingly recognized as contributing factors, along with genetic polymorphisms like *NOS2 rs2779249*, immune dysfunction characterized by altered Treg subsets, impaired checkpoint expression (*PD-1*/*PD-L1* and *Tim-3*), aberrant cytokine profiles, and dysregulated non-coding RNAs [306]. Molecular studies from Iran and Yemen confirm that *T. gondii* DNA is detectable in blood, placenta, and umbilical cord of women with abortion, but not in normal deliveries, directly linking active parasitemia and placental invasion to fetal loss [307], Table 11.

Male factor infertility accounts for approximately 50% of couple infertility cases, with a substantial portion classified as idiopathic [8,141]. An analysis of testicular biopsies from 371 azoospermic patients categorized the degree of germinal epithelial damage and attempted to correlate it with various etiopathogenetic factors, including potential infectious causes [308]. The parasite’s ability to persist in various tissues has direct implications for public health. The high seropositivity rates in food-producing animals underscore this risk, with studies in sheep demonstrating high seropositivity rates [309]. In Egyptian small ruminants, *T. gondii* seropositivity was 26.8% in live ewes and 21.2% in she-goats, with Type II (92.8%) and Type III (7.1%) genotypes identified, confirming the public health importance of these livestock species [29]. *Toxoplasma gondii* is a protozoan parasite with a broad range of intermediate hosts. Following oral infection with *T. gondii* oocysts, turkeys show widespread parasite dissemination, with a high prevalence in edible tissues like brain and thigh muscle, confirming the risk of human infection through consumption of undercooked meat [310].

The systemic nature of toxoplasmosis is also evident in human health. Seroprevalence in healthy blood donors can be significant, as demonstrated by a study in Ankara, Turkey, which found an overall rate of 42.5% [311]. In immunocompromised populations, such as those with AIDS, the parasite can cause disseminated disease, with testicular involvement documented in autopsy studies [147]. Clinical outcome data from pregnant women with a bad obstetric history reveal that toxoplasmosis is associated with 38% complete abortion, 6% stillbirths, 16% premature delivery, and 6% congenital anomalies [213]. There are no previous studies about ocular toxoplasmosis and serum levels of dehydroepiandrosterone sulphated hormone (DHEAS). Studies comparing DHEAS levels between individuals with chronic asymptomatic infection, chronic asymptomatic patients with retinal scars of retinochoroiditis, acute symptomatic patients with active retinochoroiditis, and individuals with negative assays for IgG anti-*T. gondii* show no significant differences in serum levels of DHEAS between groups when age and sex are controlled. DHEAS levels are not significantly different in active ocular toxoplasmosis related to non-active or non-infected persons [312].

### 6.1. Wildlife and Conservation Implications

Toxoplasmosis poses significant threats to wildlife populations. In endangered Hector’s dolphins (*Cephalorhynchushectori*) endemic to New Zealand, 7 of 28 (25%) dolphins examined died due to disseminated toxoplasmosis, with an additional 10 dolphins having *T. gondii* DNA detected in tissues. Genotyping revealed an atypical Type II genotype in 7 of 8 successfully amplified isolates. Fatal cases exhibited necrotising and haemorrhagic lesions in the lung, lymph nodes, liver, and adrenals, with tachyzoites and tissue cysts present in the brain, heart, stomach, and uterus. One dolphin had marked suppurative metritis with numerous intra-epithelial tachyzoites. This provides the first evidence that infectious agents could be important in the population decline of this endangered species and highlights the need for research into how *T. gondii* enters the marine environment worldwide [32].

### 6.2. Differential Diagnosis and Co-Infections

The etiological complexity of reproductive diseases is highlighted by studies in female goats, where pathological involvement of the uterus (62.96%) was associated with various pathogens including *Campylobacter* spp., *B. melitensis*, and *Chlamydophila* spp. [313]. In humans, a case of uterine gas gangrene was reported as a co-infection of *T. gondii* and Clostridium perfringens following term pregnancy, suggesting that co-infections may explain severe presentations like myonecrosis [314]. The parasite’s effects are not limited to the reproductive tract. Research on large cohorts, such as the FACE-SZ study in schizophrenia patients, has identified latent toxoplasmosis as a factor of interest, contributing to a model of low-grade peripheral inflammation in a subset of patients [315]. The ocular lesions of toxoplasmosis, which may occur in up to 25% of infected individuals, can result from both congenital and post-natal infection [316].

### 6.3. Ultrastructural and Gender Effects

Toxoplasmosis increases lymphocyte trafficking through lymph node post-capillary venules while paradoxically reducing endothelial Golgi apparatus content and increasing ribosomes, suggesting a redirection from protein processing to synthesis. These ultrastructural changes are modulated by sex hormones, with females showing greater baseline endothelial Golgi content that is reduced by oophorectomy and enhanced by estrogen treatment [75,78], Table 10.

### 6.4. Recommendations for Clinical Practice

The mechanistic evidence summarized in this review supports specific, actionable changes to clinical practice that extend beyond current diagnostic and therapeutic paradigms. First, given that *T. gondii* colonizes endometrial and testicular tissues even in seronegative individuals [5], routine infertility workups should be expanded to include PCR-based detection of parasite DNA in menstrual blood, endometrial biopsies, and semen samples, particularly for couples with recurrent implantation failure or unexplained infertility [5,16,157,172]. Second, strain genotyping should be integrated into the evaluation of adverse pregnancy outcomes, as the H2 haplotype significantly modulates abortion risk (90% in susceptible mouse strains versus 50% in resistant strains) [64,65], and distinct parasite genotypes (e.g., Type II vs. III) exhibit differential virulence and tissue tropism [29]; therefore, genotyping of placental or fetal tissues following pregnancy loss could inform prognosis and guide strain-specific management. Third, standard antiparasitic therapy (pyrimethamine-sulfadiazine) should be supplemented with immunomodulatory adjuncts, as conventional agents fail to adequately penetrate the blood–testis barrier and do not reverse the Th1-skewed immunopathology or Treg apoptosis that drive reproductive tissue damage [57]. Adjunctive strategies with preclinical support include IL-10 or TGF-β1 analogs to reduce trophoblast pyroptosis [63,176,305] and natural products such as IOP, which restores hormonal balance, mitigates oxidative stress via Nrf2 activation, and improves pregnancy outcomes in animal models [80,171]. Implementing these evidence-based recommendations would shift the clinical paradigm from serology-dependent diagnosis and parasite-centric therapy toward an integrated approach that addresses direct parasitism, immunopathology, and endocrine disruption in both sexes (Table 12).

## 7. Synthesis and Conclusions

*Toxoplasma gondii* directly causes reproductive dysfunction in both sexes through mechanistically convergent pathways involving barrier breach, immunopathology, oxidative stress, cell death, and bidirectional hormonal disruption, establishing that this ubiquitous parasite represents a fundamental threat to fertility and pregnancy outcomes rather than merely a risk for congenital infection. In females, the parasite creates persistent uterine reservoirs, triggers decidual immune dysregulation characterized by NK cell cytotoxicity, M1 macrophage polarization, Treg apoptosis, and inflammasome-mediated pyroptosis, while disrupting estrogen and progesterone signaling through both host receptor modulation and intrinsic parasite steroidogenic enzymes including *TgCYP450mt*, *TgMAPR*, and *Tg-HSD*. In males, *T. gondii* breaches the blood–testis barrier, induces germ cell and Leydig cell apoptosis via ER stress and caspase pathways, impairs sperm quality parameters across acute and chronic infection, and disrupts the hypothalamic–pituitary–gonadal axis with complex, context-dependent effects on testosterone regulation. Conserved molecular mechanisms—including *NLRP3* inflammasome activation, *PERK*/*eIF2α*/*ATF4*/*CHOP*-mediated ER stress, and *PI3K*/*AKT*/*mTOR* dysregulation—operate in both reproductive tissues, while shared host response genes including *Nlrp5*, *Insc*, and *Gbp7* suggest common pathogenic foundations. The detection of intact parasite cysts in human semen from latently infected immunocompetent men and evidence of sexual transmission in animal models raises critical questions about venereal spread, though direct transmission in humans remains unproven. Therapeutic approaches must address the pathogenic triad of direct parasitism, immunopathology, and oxidative stress, with immunomodulatory strategies and natural products like IOP and ginseng polysaccharide showing particular promise, while conventional antiparasitics face limitations due to poor reproductive sanctuary penetration and toxicity.

Longitudinal cohort studies are urgently needed to determine the duration, frequency, and viability of seminal parasite shedding in infected humans and to definitively establish whether sexual transmission occurs at clinically significant rates through serodiscordant couple studies with strain genotyping. Systematic comparison of strain-specific pathogenic mechanisms must evaluate how diverse *T. gondii* genotypes affect placental invasion, testicular tropism, and vertical transmission, moving beyond laboratory-adapted strains to include atypical isolates. Immunomodulatory therapies targeting *Trem2*, *Tim-3*, and the *NLRP3* inflammasome require preclinical development and testing in validated mouse models of reproductive toxoplasmosis, with particular attention to optimal timing relative to infection and pregnancy. Transgenerational and epigenetic effects demand investigation through sperm small RNA profiling and methylation studies in offspring of infected males to determine whether heritable reproductive or behavioral alterations occur in humans. Clinical diagnostic improvement should focus on validating PCR-based assays on endometrial biopsies, menstrual blood, and semen samples, alongside biomarker discovery to distinguish active reproductive tract infection from latent systemic disease. Natural products including IOP, ginseng polysaccharide, *Copaifera* oleoresins, and prenylated chalcones should advance to Phase I/II clinical trials, while nanocarrier platforms require biodistribution studies specifically evaluating penetration of the blood–testis and placental barriers. Wildlife conservation efforts must identify environmental transmission routes and evaluate vaccination strategies for endangered species like Hector’s dolphins. Co-infection and host genetic studies should examine synergistic effects with other reproductive pathogens and polymorphisms in *TLR11*, MyD88, cytokine, and hormone receptor genes. Elucidation of parasite steroidogenic mechanisms through targeted gene disruption and inhibitor development against *TgCYP450mt*, *TgMAPR*, and *Tg-HSD* offers novel therapeutic avenues. Finally, vaccine development must prioritize advancing mRNA and VLP candidates into non-human primate models with pregnancy outcomes as endpoints, developing transmission-blocking vaccines targeting feline oocyst shedding, and establishing correlates of protective immunity against reproductive disease.

## Figures and Tables

**Table 1:** Immune Dysregulation at the Maternal–Fetal Interface.

Cell Population	Normal Function	*T. gondii*-Induced Changes	Molecular Mechanisms	Functional Consequences	Ref.
Uterine Innate Lymphoid Cells (uILCs)	Pregnancy-supportive (GATA-3^+^, *RORγt*^+^)	Shift to Th1-like phenotype (*T-bet*^+^)	Transcriptional reprogramming	↑ IFN-γ, TNF-α; ↓ IL-5, IL-17	[60]
Decidual NK Cells (dNK)	Angiogenesis, trophoblast invasion	Cytotoxic skewing; expansion of *CD56dimCD16*^+^ subset	↑ *NKG2D*; ↓ *KIR2DL4*, *ILT-2*	↑ IFN-γ secretion, ↑ killing capacity	[37]
		*Tim-3* checkpoint downregulation	*PI3K*/*AKT*, *JAK*-*STAT* activation	↑ Granzyme B, perforin	[41]
		s*HLA-G* dysregulation	Caspase-mediated apoptosis	dNK apoptosis; potential immune evasion	[42,43]
		trNK cell functional deficiency	Unknown	Fetal growth restriction (rescued by adoptive transfer)	[45]
		*PD-1*/*PD-L1* upregulation	Altered NK cell subsets	Suppressed NK cell function	[47]
Decidual Macrophages (dMφ)	Immunosuppressive M2 phenotype	Polarization to pro-inflammatory M1	↓ *CD206*, arginase-1; ↑ *iNOS*, *CD80*, *CD86*, TNF-α, IL-12	Maternal–fetal tolerance disruption	[48]
		*TgROP18*-mediated *CD73* downregulation	*LSD1*/*SNAIL1* pathway →↓ adenosine/*A2AR*/*PKA*/*p-CREB*/*C/EBPβ*	↓ *Arg-1*, IL-10	[51,52,53,54]
		*TgROP18*-mediated *Gal-9* suppression	*JNK*/*FOXO1* pathway	dNK dysfunction via *Gal-9*/*Tim-3*	[51,53]
		*Trem2* downregulation	Impaired *Trem2*-*Syk*-*PI3K*, *Trem2*-*PPARγ*-*STAT6*	Impaired trophoblast proliferation/migration/invasion	[53,56]
Decidual Dendritic Cells	Antigen presentation	IL-12 production	Unknown	Amplifies dNK IFN-γ, *NKG2D*, cytotoxicity	[37,60]
Regulatory T Cells (Tregs)	Fetal tolerance	Apoptosis	↑ *Caspase-3*, *Caspase-8*	↓ Immunotolerance	[57,58]
		*PD-1* upregulation on surviving cells	Unknown	Net reduction in suppressive capacity	[58,59]
Myeloid-Derived Suppressor Cells (MDSCs)	Immune regulation	↓ IDO expression	*SOCS3*-mediated degradation →↓ *Kyn*/*AhR*/*SP1*	↓ TGF-β, IL-10; impaired dNK suppression	[53,59]
Single-Cell Transcriptomics	N/A	17 immune cell clusters with proportion changes	279 (dNK), 312 (dMφ), 380 (dT cells) differentially expressed genes	21 genes with altered expression	[16]

↑ = increased (expression); ↓ = decreased (expression); → = proceed to.

**Table 2:** Comparison of *T. gondii*-Induced Hypothalamic–Pituitary–Gonadal Axis Dysfunction in Female Mice and Male Humans.

Feature	Mice (Females) [6,7]	Humans (Males) [126]
Infection stage	Chronic	Acute
Observed defect	Absent LH surge, absent FSH-driven ovarian compensation	Low FSH, LH, and testosterone (hypogonadotropic hypogonadism)
Site of defect	Hypothalamus (inhibited pulsatile GnRH)	Hypothalamus (suppressed GnRH)
Proposed mediator	Cytokines reaching the hypothalamus	IL-1β (negative correlation with gonadotropins)
Conclusion	GnRH inhibition leads to pituitary-ovarian axis impairment	IL-1β suppresses GnRH, causing temporary hypogonadotropic hypogonadism

**Table 3:** *Toxoplasma gondii* shedding duration and transmission evidence in different speices.

Species	Shedding Duration	Transmission Evidence	Ref.
Sheep	Brief (14–26 days)	Documented	[131,132]
Goats	Up to 52 days	Limited replication	[139]
Rams (field)	High PCR positivity (51%)	Significance unclear	[145]
Cats	No detectable shedding	Negative	[144]
Mice	Parasite in semen	Transmission not successful	[140]
Rabbits	Up to 29 days	Uniformly unsuccessful	[142]
Humans	Cysts in latent infection	Oral transmission not demonstrated	[156,157]

**Table 4:** Tissue Tropism and Parasite Localization in the Reproductive Tract.

Tissue	Species	Detection Method	Key Findings	Ref.
Female Reproductive Tract				
Endometrial biopsies	Human	Microscopy, culture	Tachyzoites in 5/6 seronegative women with habitual abortion; cleared with treatment	[5]
Placenta	Human	PCR (*B1* gene)	86.7% positive in acute infection; 60% positive in IgG+/IgM− women	[34,35]
Uterine tissue	Cow, ewe	PCR, genotyping	Type II (92.8%) and III (7.1%) strains; distribution throughout the genital tract	[27,28,29]
Uterus	Red deer	PCR	Detected across all trimesters; associated with abortion	[33]
Uterus	Hector’s dolphin	Histology, PCR	Suppurative metritis with intra-epithelial tachyzoites	[32]
Cervical smears, menstrual blood	Human	Microscopy	Active replication within the reproductive tract	[5]
Abortus material	Human	Isolation	Early isolation from abortus specimens	[24,25]
Male Reproductive Tract				
Testis	Mouse	Bioluminescence imaging	Intense signal days 4–5 post-infection (R = 0.81 with viable counts)	[12]
Testis	Human (AIDS)	Histology, IHC	3.7–39% of autopsy cases; pseudocysts within necroses	[146,147]
Testis	Human	PCR	Detected in testicular cancer tissue	[158]
Testis, epididymis	Mouse, rat	Histology	Inflammatory infiltration, architectural disruption	[129,130]
Semen	Ram	PCR, mouse inoculation	12.5% positive at 15 weeks post-infection; prolonged shedding	[137,139]
Semen	Human	Microscopy, PCR	Cysts visualized in immunocompetent men; bradyzoite gene expression confirmed	[157]
Semen	Goat	Mouse inoculation	Shedding days 7–52 post-inoculation	[139]
Semen	Dog	PCR	8.33% positive in breeding dogs with reproductive disorders	[143]
Semen	Ram (Tunisia)	PCR	51% prevalence in farmed rams	[145]
Testicular cell culture	Lamb	Culture	Successful in vitro cultivation	[138]
Negative/Negligible Findings				
Semen	Human	Mouse inoculation	No isolation from 285 ejaculates, 23 biopsies	[142]
Semen	Rabbit	Isolation	Infective organisms only up to day 29 post-infection	[142]
Semen	Cat	PCR, histology	Not detected despite disseminated infection	[144]
Testis	Sheep (slaughterhouse)	Isolation	Only 1 isolate from 50 testicles	[142]
Reproductive tissues	Cat, dog	PCR	No DNA detected despite 22.6% seroprevalence	[159]

**Table 5:** Testicular Immunopathology and Spermatogenic Failure.

Pathway/Mechanism	Key Findings	Molecular Mediators	Functional Consequences	Ref.
Innate Immune Recognition				
MyD88 signaling	*MyD88^−^/^−^* mice: parasite loads ↑ 100×	TIR signaling via *TLR*s (not *IL-1R*/*IL-18R*)	Uncontrolled testicular dissemination	[12]
*TLR11*	Germ cell expression	Recognizes profilin	Inflammatory cytokine production	[160]
*TLR4-P2X7R/NLRP3*	Activation in infected testes	Inflammasome pathway	Inflammatory cascade	[161]
*JNK2* MAP kinase	Required for neutrophil IL-12	*MAPK* signaling	Host defense	[162]
Cytokine Milieu				
Th1 polarization	↑ IFN-γ, TNF-α, IL-6	T-cell responses	Bystander damage to germ cells	[163,164]
Pro-apoptotic shift	↑ *Bax* in spermatogenic cells	Correlates with sustained damage	Germ cell depletion	[163]
Sperm Quality Parameters				
Sperm viability	41.33–48.55% vs. 75.71% controls	Membrane integrity loss	Reduced fertilizing capacity	[137]
Sperm motility	Significant decrease days 10–60 p.i.	Mitochondrial dysfunction	Impaired sperm function	[129]
Sperm concentration	Reduced days 10, 30, 40, 60 p.i.	Impaired spermatogenesis	Oligozoospermia	[129]
Morphological abnormalities	↑ Bent tail, loss of hook, detached heads (peak 30% at days 30–40)	Structural disruption	Teratozoospermia	[129,169]
DNA damage	↑ Acridine orange, aniline blue, and toluidine blue	Chromatin abnormalities	Genetic integrity compromised	[168]
Histopathological Changes				
Seminiferous tubules	Reduced diameter	Epithelial disruption	Impaired spermatogenesis	[166,167]
Germ cell populations	Depletion of spermatocytes, spermatids	Apoptosis	Reduced sperm output	[113]
Blood–testis barrier	Altered gene expression (e.g., *PTGDS*)	250 differentially expressed genes	Compromised immune privilege	[172]
Direct Sperm Damage (In Vitro)				
Mitochondrial dysfunction	Loss of mitochondrial membrane potential	No ROS modulation	Headless sperm, twisted tails	[8]
Soluble antigen exposure	Impaired motility, mitochondrial activity, and membrane integrity	No effect on fertilization rate	Negative impact on embryonic development	[10]

↑ = increased (expression).

**Table 6:** Cell Death Pathways in Reproductive Tissues.

Pathway	Female (Trophoblast/Placenta)	Male (Germ Cells/Leydig Cells)	Shared Mechanisms	Ref.
Apoptosis				
Caspase-dependent	↑ *Caspase-3*, *Caspase-8*; IFN-γ-mediated (r = 0.7163 with apoptosis)	↑ *Caspase-3*, *Bax*; ↓ *Bcl-2*	Mitochondrial pathway involvement	[40,170,176]
Death receptor	Fas/CD95 modulation (strain-specific)	Unknown	Differential regulation by strain	[177]
*Bcl-2* family	*Bax*/*Bcl-2* dysregulation in recurrent miscarriage	↑ *Bax*, *CHOP*, *P53*	Pro-apoptotic shift	[170,178]
Pyroptosis				
Inflammasome activation	*NLRP1*, *NLRP3*, *NLRC4*, *AIM2* in BeWo, HTR-8/SVneo, WISH cells	*TLR4-P2X7R/NLRP3* activation	Caspase-1-mediated; gasdermin D cleavage	[63,161]
ROS generation	↑ ROS → CatB release into cytosol	Oxidative stress in the testis	Initiates inflammasome signaling	[63,174]
IL-1β maturation	↑ *ASC*, cleaved caspase-1, mature IL-1β	Inflammatory cytokine production	Pro-inflammatory cell death	[63]
Endoplasmic Reticulum Stress				
*PERK*/*eIF2α*/*ATF4*/*CHOP*	*GRA15II*-induced in trophoblasts	↑ *CHOP*, *P53* in Leydig cells	Conserved pathway across sexes	[161,170,179]
*IRE1α*-*JNK*	Activated by *GRA15II*	Unknown	Apoptosis induction	[179]
Oxidative Stress				
Antioxidant depletion	Mitochondrial dysfunction	↓ SOD, catalase, GSH; ↑ MDA	Compromised cellular defense	
NADPH oxidase	ROS production	Unknown	Mitochondrial dysfunction	[174,180]
Nrf2 pathway	Protective role	↑ SOD, GSH, HO-1, NQO-1 with IOP treatment	Therapeutic target	[171]
Bystander Effects				
Paracrine killing	Protection of parasitized cells; killing of bystanders	Inflammatory-mediated damage	Indirect pathology	[181]
Strain-specific modulation	*RH* strain inhibits apoptosis; *ME49* promotes it	Differential virulence factors	Virulence-dependent outcomes	[177,182]

↑ = increased (expression); ↓ = decreased (expression); → = proceed to.

**Table 7:** Hormonal Disruption and Endocrine Interactions.

Hormone/Pathway	Female Reproductive Effects	Male Reproductive Effects	Parasite-Directed Mechanisms	Ref.
Estrogen/Estradiol				
Hormone levels	Fluctuate with the gestational stage	↑ in latently infected men (meta-analysis: +0.73 units)	*Tg-HSD* transforms estrone → estradiol	[1,71]
Immune modulation	*ERK*/*AKT* activation in infected macrophages; modulates *PRLR*, *ERα*, *GPER*	↑ parasite burden in neural cells at 100–250 nM	Receptor modulation; tamoxifen antagonizes	[9,14,74]
Treg regulation	Protects Tregs from apoptosis via *ERα*; ↑ *PD-1* expression	N/A	Gestational age-dependent effects	[79]
Pharmacological effects	High-dose estrogens ↑ brain cysts	N/A	Antagonized by tamoxifen	[74]
Progesterone				
Hormone levels	↓ in infected pregnant mice; elevated in infected female rats	No significant in vitro effects	Direct parasite interaction via *TgPGRMC*	[94,100,103]
Parasite inhibition	Inhibits tachyzoite invasion/proliferation	No effect on replication in RAW cells	*TgPGRMC* knockout confers resistance	[94,107]
Immune modulation	↓ NO via glucocorticoid receptor; ↓ IL-12	N/A	Receptor-specific effects	[97]
Testosterone				
Serum levels	↓ in infected women	Variable: ↑ in human meta-analysis; ↓ in most rodent models	Leydig cell apoptosis; ↓ *StAR*, *P450scc*, *17β-HSD*	[1,21,22,161,171]
Intratesticular	N/A	Consistently ↓ in chronic infection	Steroidogenic enzyme downregulation	[166,187]
Behavioral effects	↑ dominance perception in men; ↑ attractiveness to females	↑ risk tolerance; ↓ fear of cat odor	Manipulation strategy for transmission	[183,192,197]
HPG Axis				
LH	N/A	↓ in chronic infection	Cytokine-mediated GnRH suppression	[166]
FSH	N/A	↑ in chronic infection	Compensatory response to testicular failure	[113,166]
GnRH	Hypothalamic dysfunction	Probable suppression	Cytokine-mediated	[6,7,31]
Parasite Steroidogenic Machinery				
*TgCYP450mt*	Mitochondrial *P450* enzyme	Mitochondrial *P450* enzyme	Endogenous hormone synthesis	[19]
*TgMAPR*	Membrane-associated progesterone receptor	Membrane-associated progesterone receptor	Supports steroidogenesis; interacts with *TgCYP450mt*	[19]
*Tg-HSD*	Hydroxysteroid dehydrogenase	Hydroxysteroid dehydrogenase	Transforms estrone → estradiol	[71]
*TgPGRMC*	Progesterone membrane receptor	Progesterone membrane receptor	Mediates progesterone effects	[94]
Sex-Specific Differences				
2D:4D digit ratio	Altered in infected women	Altered in infected men	Reflects prenatal hormone exposure	[21,22,23]
Behavioral changes	Less risk-averse; influenced by the estrus cycle	↓ cat odor aversion (AVP-dependent)	Different neuroendocrine mechanisms	[103,104]
Mortality/susceptibility	Females die earlier post-infection	Protected by testosterone	Testosterone reduces intestinal pathology	[165]

↑ = increased (expression); ↓ = decreased (expression); → = proceed to.

**Table 8:** Vertical Transmission Dynamics is different for different species.

Parameter	Findings	Species	Key Factors	Ref.
Timing of Transmission				
First trimester	The decidualsite with extravillous trophoblasts is most vulnerable	Human (explant)	Greater vulnerability than the villous region	[11]
Early pregnancy	Blastocyst-endometrial relationship stage	*Calomys callosus*	p30-containing trails in the ECM	[203]
Days 6–11 post-coitum	60.6% congenital transmission	Mouse (acute)	Minocycline reduces to 3.6%	[211]
Days 11–15 post-inoculation	Parasite reaches fetal tissues	Goat	Preferential isolation: muscle, heart, lung, brain	[204,205]
All trimesters	Associated with abortion	Red deer	Detected in the uterus, cotyledons, and fetal brains	[33]
Placental Infection				
Without fetal infection	*B1* gene in 86.7% (acute), 60% (chronic) placenta	Human	IgM negative in cord blood	[34,35]
Necrotizing placentitis	Tachyzoites in the trophoblasts lining areolae	Sow	Non-suppurative encephalomyelitis in fetuses	[69]
Granulomatous placentitis	Mature immune reaction	Human	Cause of first-trimester abortion	[70]
Fetal Outcomes				
Complete abortion	38% of cases with bad obstetric history	Human	Associated with toxoplasmosis	[213]
Stillbirth	6% of cases	Human	Fetal death precedes expulsion by 1–12 days	[85,213]
Premature delivery	16% of cases	Human	Varies by gestational age	[213]
Congenital anomalies	6% of cases	Human	Severe sequelae possible	[213]
Fetal mummification	Variable interval to expulsion	Goat	Fresh to mummified appearance	[85]
Endocrine Changes During Pregnancy				
Progesterone	↓ in infected pregnant mice	Mouse	Correlates with adverse outcomes	[100]
15-ketodihydro-PGF2α	↑ from day 40 onward	Goat, sheep	Indicates inflammation, luteolysis	[85,101]
Oestrone sulphate	Failure to rise normally	Goat, sheep	Reflects placental dysfunction	[85,101]
Protective Immunity				
IFN-γ	Essential for protection; absence ↑ of uterine/placental loads	Mouse (KO)	Fetal infection only in IFN-γ KO *C57BL/6*	[212]
Genetic background	*C57BL/6* (H2b): 90% abortion; *BALB/c*: 50%	Mouse	Higher TNF-α, inflammatory foci in *C57BL/6*	[64,65]
*FOXP3* expression	↓ at the maternal–fetal interface; greater ↓ in *C57BL/6*	Mouse	Correlates with susceptibility	[62]
Prior immunity	Reinfection with a different strain can transmit	*Calomys callosus*	Brazilian strains more severe	[207,208]

↑ = increased (expression); ↓ = decreased (expression).

**Table 9:** Evidence for and Against Sexual Transmission.

Evidence Type	Supporting Evidence	Contradictory/Negative Evidence	Species	Ref.
Parasite in Semen				
Direct visualization	Cysts in the semen of immunocompetent men	Not observed in husbands of infected women	Human	[5,157]
DNA detection	51% in ram semen; 8.33% in dog semen	Not detected in cat semen	Ram, Dog, Cat	[143,144,145]
Infective parasites	Isolated from goat semen days 7–52 p.i.	Only transient in ram semen (days 14–26 p.i.)	Goat, Ram	[131,139]
Artificial Insemination Studies				
Sheep	Successful transmission with contaminated semen	N/A	Sheep	[133,134]
Goats	Seroconversion, embryonic reabsorption	N/A	Goat	[216]
Rabbits	Successful transmission	N/A	Rabbit	[220]
Dogs	Seroconversion, fetal reabsorption, cysts in offspring	N/A	Dog	[218]
Natural Mating Studies				
Sheep	Ewes seroconverted; vertical transmission	Latent infection: no reactivation with immunosuppression	Sheep	[132,135]
Rabbits	No transmission to *T. gondii*-free females	Attempts uniformly unsuccessful	Rabbit	[142]
Epidemiological Evidence				
Congenital toxoplasmosis	Cases without identified risk factors	N/A	Human	[214]
Serodiscordant couples	Hypothesized transmission route	Direct evidence lacking	Human	[214]
Reproductive Tissue Presence				
Testicular infection	Confirmed in multiple species	Not always detected despite seropositivity	Various	Multiple Ref.
Genital tract distribution	Throughout the male and female tracts	Intermittent shedding common	Various	Multiple Ref.
Current Consensus	Biologically plausible; less efficient than the oral–fecal route; clinical significance may be limited to specific contexts (serodiscordant couples, artificial insemination)			

**Table 10:** Therapeutic Strategies for Reproductive Toxoplasmosis.

Therapeutic Category	Agent/Approach	Mechanism of Action	Efficacy/Findings	Sex Affected	Ref.
Conventional Antiparasitic					
Folate antagonist + sulfonamide	Pyrimethamine + Sulfadiazine	Inhibit folate synthesis	Parasite suppression; poor reproductive sanctuary penetration; bone marrow suppression	Both	[221]
Sulfonamide	Sulphadimidine	Inhibit folate synthesis	Failed to prevent sperm deterioration in rams	Male	[137]
Nitrofuran	Nitrofurantoin ± Spiramycin	DNA damage	↓ Parasite burden, uterine inflammation	Female	[222]
Triazine + naphthoquinone	Diclazuril + Atovaquone	Mitochondrial inhibition	Synergistic pregnancy protection	Female	[223]
Ionophore	Monensin	Cation ionophore	↓ Lamb losses (16.7% vs. 55.2%); heavier lambs	Female (ewe)	[224]
Immunomodulatory					
Cytokine therapy	TGF-β1	↓ dNK cytotoxicity via *NKG2D*/DAP10	Improved pregnancy outcomes	Female	[305]
	IL-10 (recombinant)	↓ Treg apoptosis; ↓ *caspase-3*, -8; ↑ *c-FLIP*	Improved pregnancy outcomes; regulates *HLA-G*	Female	[57,176]
Checkpoint modulation	*Tim-3* pathway	Restores immune balance	Protective in animal models	Female	[41]
	*Trem2* agonism	*Syk*-*PI3K*, *PPARγ*-*STAT6* signaling	Protects against adverse pregnancy outcomes	Female	
Natural Products					
Fungal polysaccharide	*Inonotus obliquus* polysaccharide	*TLR4/NF-κB*; *Th17/Treg*; Nrf2; *PI3K*/*AKT*/*mTOR*	↓ Abortion rates; ↑ progesterone, estriol; antioxidant; restores testosterone, LH, FSH; ↑ *StAR*, *P450scc*, *17β-HSD*	Both	[53,56,80,171]
Herbal polysaccharide	Ginseng polysaccharide	*TLR4-P2X7R/NLRP3*; *PERK*/*eIF2α*	↓ Inflammation; ↓ ER stress apoptosis; restores hormones	Male	[161]
Resin	*Copaifera* oleoresins	Immunomodulation; direct antiparasitic; cell cycle arrest (S/M phase)	Irreversible antiparasitic action in trophoblasts	Female	[227]
Flavonoids	Prenylated chalcones	Ultrastructural damage; ↑ IL-8; ↓ MIF, ROS, TNF-α	Impaired parasite invasion	Female	[228]
Amino acid derivative	S-methylcysteine + Spiramycin	Hormonal restoration	Tissue recovery in the uterus andovary	Female	[88]
Bee product + oil	Propolis + Wheat germ oil	↓ Parasite load; histopathological restoration	Restored brain, uterus, and kidney damage	Female	[225,226]
Nanomedicine					
Biogenic nanoparticles	AgNp-Bio (silver)	Autophagic vacuole accumulation; ↓ intracellular tachyzoites	No Leydig cell cytotoxicity at low doses	Male	[221]
Vaccination					
Cross-species	*Hammondiahammondi* oocysts	Cross-protective immunity	4/5 goats gave birth to healthy kids; but *T. gondii* still isolated	Female (goat)	[204,205]
Iscom vaccine	Immunostimulating complexes	↓ Parasite dissemination; preserves endocrine profiles	Normal progesterone, oestrone sulphate in challenged ewes	Female (sheep)	[101,292]
Drug Safety Concerns					
SERM	Tamoxifen	Estrogen antagonist	Exacerbates chronic infection; severe endometrial necrosis	Female	[302]
Antifolate	Pyrimethamine	Folate antagonist	Testicular developmental delay in neonates; partially mitigated by folic acid	Male (neonatal)	[303]
NSAID	Flunixin meglumine	COX inhibitor (PGF2α suppression)	Could not prevent abortion; infectious process unaffected	Female (sheep)	[304]

↑ = increased (expression); ↓ = decreased (expression).

**Table 11:** Molecular and Genomic Changes in Reproductive Tissues.

Tissue/Condition	Analysis Type	Key Findings	Differentially Expressed Genes/Proteins	Pathways Affected	Ref.
Uterus					
Pre-pregnancy infection	Transcriptomics	4561 differentially expressed genes	Anatomical structure development		[200]
Post-pregnancy infection	Transcriptomics	2345 differentially expressed genes	Hormone biosynthesis		
Post-implantation infection	Transcriptomics	2997 differentially expressed genes	Cytokine–cytokine receptor interactions		
Testis					
Chronic infection	Transcriptomics	250 differentially expressed genes	*PTGDS* (blood–testis barrier)	Spermatogenic microenvironment	[172]
Cross-Tissue Comparison					
Testis and uterus	Transcriptomics	Shared host response genes	*Nlrp5*, *Insc*, *Gbp7*	Conserved reproductive damage mechanisms	[42]
		Shared defense genes	*Gbp2b*, *Ifit3*	Host defense upregulation	
Decidual Immune Cells					
dNK, dMφ, dT cells	Single-cell RNA-seq	17 immune cell clusters with proportion changes	279 (dNK), 312 (dMφ), 380 (dT cells) genes	21 novel molecules identified	[16]
Decidual immune cells	Proteomics	181 dysregulated proteins	11 verified proteins	Trophoblast invasion, placental development, and immune tolerance	[90]
Parasite-Directed Mechanisms					
PV formation	Protein recruitment	*MOSPD2* recruited to the PV membrane (strain-dependent)	*MOSPD2*	Functional significance under investigation	[13]
Virulence factors	Functional analysis	*TgROP18*	*CD73*, *Gal-9* regulation	Immune modulation	[53,55]
Steroidogenesis	Lipidomics, genetics	Endogenous hormone synthesis	*TgCYP450mt*, *TgMAPR*	Mitochondrial integrity, virulence	[19]
	Functional analysis	Estrogen synthesis	*Tg-HSD*	Pathogenicity enhancement	[71]
	Functional analysis	Progesterone response	*TgPGRMC*	Progesterone sensitivity	[94]

**Table 12:** Clinical and Epidemiological Implications.

Category	Finding	Population/Species	Significance	Ref.
Pregnancy Outcomes				
Spontaneous abortion	15–20% of clinical pregnancies	Human	*T. gondii* as a contributing factor; requires endometrial proof	[109]
Recurrent pregnancy loss (RPL)	Affects ~5% of couples; 50% idiopathic	Human	Environmental triggers include *T. gondii*; genetic polymorphisms (*NOS2 rs2779249*); immune dysfunction (Treg, *PD-1*/*PD-L1*, *Tim-3*)	[306]
Bad obstetric history	38% complete abortion; 6% stillbirth; 16% premature delivery; 6% congenital anomalies	Human	Toxoplasmosis-associated outcomes	[213]
Male Infertility				
Male factor infertility	~50% of couples’ infertility	Human	Substantial portion idiopathic	[8,141]
Azoospermia	Germinal epithelial damage grading	Human (371 biopsies)	Potential infectious causes	[308]
Livestock and Food Safety				
Seropositivity in food animals	26.8% in ewes; 21.2% in she-goats	Egyptian small ruminants	Type II (92.8%) and III (7.1%) genotypes; public health importance	[29]
Tissue cysts in edible organs	Brain, thigh muscle	Turkey	Risk from undercooked meat	[310]
High seropositivity	Variable rates	Sheep	Foodborne transmission risk	[309]
Human Seroprevalence				
Blood donors	42.5%	Ankara, Turkey	Significant background exposure	[311]
Immunocompromised Hosts				
AIDS patients	Testicular involvement in 3.7–39%	Human	Disseminated disease; often only extracerebral manifestation	[146,147]
Wildlife and Conservation				
Endangered Hector’s dolphins	7/28 (25%) died from disseminated toxoplasmosis; 10 additional DNA-positive	New Zealand	First evidence that infectious agents contribute to population decline; atypical Type II genotype; suppurative metritis with tachyzoites	[32]
Differential Diagnosis and Co-infections				
Caprine reproductive disease	62.96% uterine pathology	Goat	Co-pathogens: *Campylobacter* spp., B. melitensis, *Chlamydophila* spp.	[313]
Uterine gas gangrene	Co-infection with *C. perfringens*	Human	Severe presentation post-term pregnancy	[314]
Systemic Effects				
Schizophrenia	Latent toxoplasmosis as a factor	Human (FACE-SZ study)	Low-grade peripheral inflammation in a subset	[315]
Ocular toxoplasmosis	Up to 25% of infected individuals	Human	Congenital and post-natal infection	[316]
DHEAS levels	No significant difference in ocular toxoplasmosis	Human	Age- and sex-controlled	[312]
Hepatic Effects				
Drug-metabolizing enzymes	Altered cytochrome *P450* expression	Mouse	Implications for drug efficacy/toxicity in infected individuals	[201]

## Data Availability

No new data were created or analyzed in this study. Data sharing is not applicable to this article.
